# Expansion and Functional Divergence of Inositol Polyphosphate 5-Phosphatases in Angiosperms

**DOI:** 10.3390/genes10050393

**Published:** 2019-05-22

**Authors:** Zaibao Zhang, Yuting Li, Zhaoyi Luo, Shuwei Kong, Yilin Zhao, Chi Zhang, Wei Zhang, Hongyu Yuan, Lin Cheng

**Affiliations:** 1 Henan Key Laboratory of Tea Plant Biology, Xinyang Normal University, Xinyang 464000, China; zaibaozhang79@163.com; 2 College of Life Science, Xinyang Normal University, Xinyang 464000, China; Liyuting0721@163.com (Y.L.); zhaoyiluo413@163.com (Z.L.); Kongshuwi@163.com (S.K.); yilinzhao10@163.com (Y.Z.); zhangchi6666666@163.com (C.Z.); chawenhua2009@163.com (W.Z.)

**Keywords:** gene duplication, gene fate, inositol polyphosphate 5-phosphatase, phosphatidylinositol signaling

## Abstract

Inositol polyphosphate 5-phosphatase (5PTase), a key enzyme that hydrolyzes the 5′ position of the inositol ring, has essential functions in growth, development, and stress responses in plants, yeasts, and animals. However, the evolutionary history and patterns of 5PTases have not been examined systematically. Here, we report a comprehensive molecular evolutionary analysis of the *5PTase* gene family and define four groups. These four groups are different from former classifications, which were based on in vitro substrate specificity. Most orthologous groups appear to be conserved as single or low-copy genes in all lineages in Groups II–IV, whereas *5PTase* genes in Group I underwent several duplication events in angiosperm, resulting in multiple gene copies. Whole-genome duplication (WGD) was the main mechanism for *5PTase* duplications in angiosperm. Plant 5PTases have more members than that of animals, and most plant *5PTase* genes appear to have evolved under strong purifying selection. The paralogs have diverged in substrate specificity and expression pattern, showing evidence of selection pressure. Meanwhile, the increase in 5PTases and divergences in sequence, expression, and substrate might have contributed to the divergent functions of *5PTase* genes, allowing the angiosperms to successfully adapt to a great number of ecological niches.

## 1. Introduction

**Figure 1:**
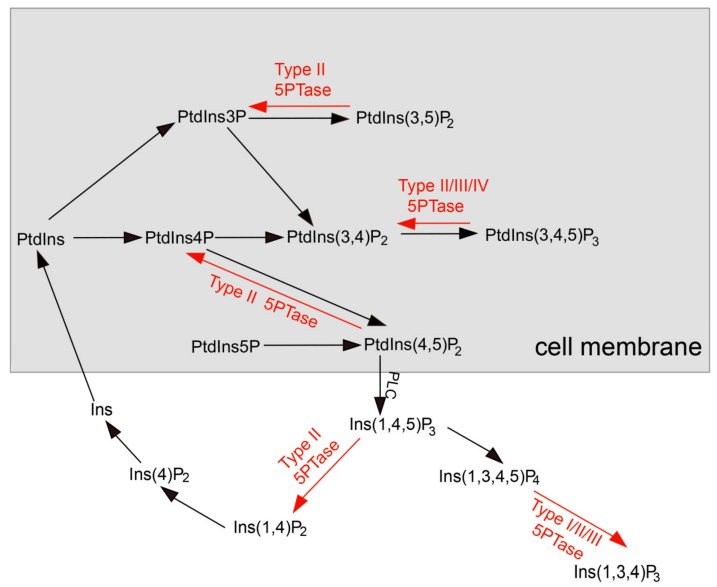
The phosphoinositide metabolic pathway in plants. The free hydroxyl groups of the phosphatidylinositols (PtdIns) can be phosphorylated at positions 3, 4, and/or 5 by distinct kinases (black arrows) to generate various phosphoinositides. Phospholipase C (PLC) can convert substrate PtdIns(4,5)P_2_ into the second messenger Ins(1,4,5)P_3_. The action of inositol polyphosphate 5-phosphatases (red arrows) modulates the signaling functions of these molecules.

Phosphoinositides are phospholipids that are present ubiquitously in all eukaryotic cell membranes which regulate numerous cellular processes, including vesicular trafficking, cytoskeletal dynamics, proliferation, and survival [1,2,3,4]. The spatial and temporal localization of phosphoinositide signals is tightly regulated by phosphoinositide kinases and phosphatases, including the inositol polyphosphate 5-phosphatases (5PTases) (Figure 1). 5PTases, one of the key enzymes in the phosphatidylinositol (PI) pathway, are found in all kingdoms of life, and can dephosphorylate the second messenger molecules, inositol 1,4,5-trisphosphate (Ins(1,4,5)P_3_) and inositol 1,3,4,5-tetrakisphosphate (Ins(1,3,4,5)P_4_) [2]. The 5PTases contain a highly conserved catalytic 5-phosphatase domain with a length of approximately 350 amino acids. Previous studies have demonstrated the structure of the 5-phosphatase catalytic domain of *Schizosaccharomyces pombe* synaptojanin (SP synaptojanin), which is similar to those of Mg^2+^-dependent nucleases [5]. However, the catalytic mechanism of 5PTases remains unclear at present.

5Ptases regulate a range of cellular processes involved in growth, development, and stress responses in plants, yeasts, and mammals [6,7,8,9,10]. 5PTases exhibit different substrate specificities, and hydrolyze the 5′ position phosphate in the inositol ring of water-soluble inositol polyphosphates (Ins(1,4,5)P_3_, Ins(1,3,4,5)P_4_), and/or phosphoinositides (PtdIns(4,5)P_2_, PtdIns(3,4,5)P_3_, PtdIns(3,5)P_2_) (Figure 1) [2]. Based on their substrate specificity, mammalian 5PTases can be further divided into four types [2]. Type I 5PTases hydrolyze phosphate from Ins(1,4,5)P_3_ and Ins(1,3,4,5)P_4_. Type II 5PTases hydrolyze phosphate from both water-soluble inositol polyphosphates and phosphoinositides. Type III 5PTases hydrolyze phosphate from PI(3,4,5)P_3_ and Ins(1,3,4,5)P_4_, and type IV 5PTases specifically dephosphorylate PI(3,4,5)P_3_. 

Eukaryotes have a family of diverse 5PTases, with four members in yeast, ten in mice, ten in humans, 15 in *Arabidopsis*, and 21 in rice [1,9,11,12,13,14,15,16]. All of the 5PTases are characterized by a highly conserved phosphatase domain of about 350 residues, and some plant 5PTases also contain several WD40 domains that function in protein–protein interactions [17,18,19]. The yeast *5PTase* genes, *INP51*, *INP52*, *INP53*, and *INP54*, show different cell localization: INP52 localizes to plasma-membrane-derived endocytic vesicles, INP53 localizes to the Golgi, endosomal, or vesicular membranes, and INP54 localizes to the endoplasmic reticulum (ER) [20,21,22]. Loss of any single yeast 5PTase has little phenotypic effect, while the loss of any two 5PTases results in marked phenotypic changes, suggesting a degree of redundancy [23]. In mammals, 5PTases play important roles in the regulation of hematopoietic stem cell proliferation, synaptic vesicle recycling, insulin signaling, endocytosis, vesicular trafficking, and actin polymerization. Loss of 5PTase function in mammals results in a broad spectrum of diseases and disorders [7,24]. For example, mutations in *OCRL1* results in the X-linked OculoCerebroRenal syndrome of Lowe and Type-2 Dent disease [25,26]. Loss of function of *SHIP1* results in myeloproliferative syndrome [27,28]. *SHIP2* is implicated in obesity, insulin resistance, and hypertension [29,30,31]. In addition, mutations in *INPP5E* can cause Lowe cerebrorenal syndromes [32]. These results reveal the functional differences in mammal 5PTases. 

Compared with the huge number of reports on mammal 5PTases, knowledge about the functions of plant 5PTases is rather limited. The *Arabidopsis* genome contains more 5PTases (At5PTases) than any yeasts or mammals [11]. Based on their protein structure, *Arabidopsis* 5PTases can be divided into two types, with 11 genes encoding type I 5PTases and four genes encoding type II 5PTases, which contain several WD40 domains [11]. Similar to mammal 5PTases, *Arabidopsis* 5PTases also display both overlapping and unique substrate preferences, suggesting the presence of different types of At5PTase with distinct biological functions. At5PTase1 and At5PTase2 both hydrolyze Ins(1,4,5)P_3_ and Ins(1,3,4,5)P_4_, and *5ptase1* and *5ptase2* single mutants show similar phenotypes, with faster seed germination and increased hypocotyl elongation when grown in the dark [33]. However, At5PTase1 and At5PTase2 are not functionally redundant, as the double mutant does not result in an additive effect [33]. The *FRAGILE FIBER3* (*RFA3*), *root-hair morphogenesis3* (*MRH3*; *At5PTase5*), *cotyledon vascular pattern2* (*CVP2*; *At5PTase6*), and *CVP2 LIKE 1* (*CVL1*; *At5PTase4*) genes are required for fiber cell development [34], root hair initiation [35], and cotyledon vascular patterning [36,37], respectively. Three type II At5PTase genes, *At5PTase12*, *At5PTase13*, and *At5PTase14* are differentially expressed in *Arabidopsis* organs. In addition, the proteins display different substrate specificity along with At5PTase12 and At5PTase13, which display phosphatase activity toward Ins(1,4,5)P_3_, while At5PTase14 hydrolyzes PI(4,5)P_2_, PI(3,4,5)P_3_, and Ins(1,4,5)P_3_, with the highest substrate affinity being towards PI(4,5)P_2_ [18]. The *At5PTase13* mutant results in defects in cotyledon vein development and root gravitropism [38,39]. In addition, mutations in *At5PTase7* and its paralog *At5PTase9* lead to a similar increased salt sensitivity phenotype with reduced production of reactive oxygen species (ROS) and decreased expression of stress-responsive genes in both mutants [40,41]. However, these two paralogs exhibit different substrate preferences, with At5PTase7 having a substrate preference for membrane-bound phosphoinositides (PtdIns(4,5)P_2_ and PtdIns(3,4,5)P_3_), whereas At5PTase9 dephosphorylates PtdIns(4,5)P_2_, PtdIns(3,5)P_2_, and PtdIns(3,4,5)P_3_) [40,41]. These results also indicate that At5PTase7 and At5PTase9 are non-redundant, similar to At5PTase1 and At5PTase2. Although the substrate preferences of many mammal and plant 5PTases have been reported, the comparative substrate specificity between mammal and plant 5PTase is not clear. Recently, maize *BREVIS PLANT1* (*BV1*), an inositol polyphosphate 5-phosphatase with a WD40 domain, was reported to be involved in internode elongation [42]. Despite these important functions, information on the origins and evolution of 5PTases remains fundamentally unexplored.

The evolutionary study of plant and mammal *5PTase* genes is very limited. Previous reports have focused on a single species or a few species only, and there has been no systematic studies of the *5PTase* family in plants or mammals. Large-scale sequenced genome data provides deeper sampling to gain insights into the evolution of the 5PTase family during the history of angiosperms. In this study, an evolutionary analysis of the *5PTase* genes in major plant lineages is presented with phylogenetic analyses. Phylogenetic analyses were performed to delineate the evolutionary history of the 5PTase family in major angiosperm lineages, and exon/intron structure analyses were performed to gain insight into the possible mechanisms of the structural diversity of the *5PTase* gene family. The tissue specificity and inducibility of *5PTase* gene expression in plants were characterized by examining publicly available microarray data. The results obtained here will broaden our understanding of the roles and evolution of 5PTases and provide a framework for further functional investigations of these genes in plants.

## 2. Materials and Methods

### 2.1. Data Sources and Homolog Searches

**Figure 2:**
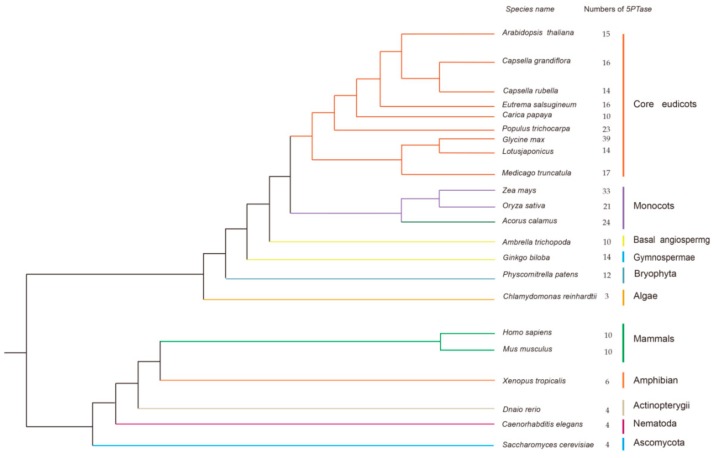
Phylogenetic relationships of species that were used in this study. The species tree was constructed based on the phylogeny of conserved nuclear genes [56,57,58]. The total number of 5PTase proteins found in the genome of each species is indicated.

Several datasets (Ensembl Genome, JGI Genome, Phytozome) and multiple steps were used to search for 5PTase sequences. We selected representative species from animals, plants, and fungi, including 16 plants, five animals, and one fungus. Proteomic data from animals and fungi were downloaded from the ENSEMBL (release 91, http://www.ensembl.org) and JGI (http://genome.jgi.doe.gov/) databases, respectively. Plant sequences were obtained from Phytozome v12.1 (http://www.phytozome.net/). The sequences for *Amborella trichopoda* were retrieved from the Amborella Genome Database (http://www.Amborella.org/). The number of *5PTase* genes in different organisms can be found in Figure 2.

The hidden Markov model (HMM) program (version 3.0) [43] was employed with the Hidden Markov model to retrieve all eukaryotic *5PTase* homologs. The Hidden Markov model profile of the 5PTase protein domain (PF03372 in Pfam database) was downloaded and used in local searches of the datasets [44]. These sequences were verified using the protein families (PFAM) database (http://pfam.xfam.org/search), the Conserved Domains Database (CDD) (http://www.ncbi.nlm.nih.gov/Structure/cdd/wrpsb.cgi), available from the National Center for Biotechnology Information, and the SMART (a Simple Modular Architecture Research Tool) database (http://smart.embl-heidelberg.de/), with a threshold *e*-value of less than 1*e*–10 [44,45,46]. 

### 2.2. Phylogenetic Analyses

Phylogenetic analyses were conducted using two methods: neighbor-joining (NJ), and maximum likelihood (ML). NJ trees were constructed using MEGA 5.0 with Poisson correction, pairwise deletion, and bootstrap (1000 replicates) [47]. Multiple sequence alignments were performed using ClustalW with default parameters in MEGA 5.0 (Table S1, Figure S2). PhyML 3.0 and RaxML v7.0.4 were used to construct ML trees, with the Jones, Taylor, and Thorton (JTT) model and gamma distribution option, and rooted using phosphoinositide-specific phospholipase C AT1G13680 and AT1G49740 as outgroups, as these two phospholipases belong to families other than the 5PTases [48,49]. 

### 2.3. Motif and Synteny Analyses

All 5PTase amino acid sequences were used to search against the PFAM, CDD, and SMART databases to uncover other known domains or motifs apart from the 5PTase domain. Multiple Em for Motif Elicitation (MEME) software (v4.9.0) was used to discover novel conserved motifs that might not be recorded in public databases [50]. Duplicate gene pairs were searched for any evidence of synteny using the Plant Genome Duplication Database (http://chibba.agtec.uga.edu/duplication) [51,52].

### 2.4. Expression Analysis

For the expression profile analysis of *5PTase* genes in *Arabidopsis* and rice, ATH1 22k and Os 51k microarray data from the Genevestigator V3 database were used, and then the heat maps were constructed using the obtained gene expression datasets, respectively [53]. Transcriptome data of soybean 5PTase homologs in representative tissues were analyzed [54]. For heat, cold, and drought stress analysis, two-week-old soybean was exposed to 42 °C, 4 °C, and PEG400 for 1 h, respectively. Each treatment consisted of three biological replicates, and each biological replicate consisted of pools of six plants. Total RNA was isolated, and RNA sequencing (RNA-seq) was performed on the Illumina HiSeqTM2000 platform.

### 2.5. Calculation of Ka/Ks-Values

The ratio of non-synonymous to synonymous substitutions (Ka/Ks) was estimated to investigate the molecular evolution of *5PTase* genes. The duplicated *5PTase* coding sequences (CDS) were aligned using ClustalW (http://www.genome.jp/tools/clustalw/) to predict the divergence periods. Based on previous research, a Ka/Ks ratio of <1 indicates purifying selection, a ratio =1 indicates neutral evolution, and a ratio >1 indicates positive selection [55].

## 3. Results

### 3.1. Identification of 5PTase Genes in Plants, Animals, and Fungi

Using the Hidden Markov model (HMM) algorithm, the complete set of *5PTases* genes were identified from a comprehensive dataset that contained selected plants, animals, and fungi. In total, 318 sequences were retrieved from 16 plants, five animals, and one fungus (Figure 2). *5PTase* genes were determined based on whether the corresponding protein contained the 5PTase domain. The 5PTase proteins that were identified ranged in size from 169 to 1916 amino acids. Among the major lineages of green plants, *5PTase* genes are present in algae, bryophyta, gymnosperms, and angiosperms (Figure 2). Further investigation revealed that the copy number of the *5PTase* genes varied considerably among plants, ranging from three in the green algae *Chlamydomonas reinhardtii* to 12 in *Physcomitrella patens* (bryophyta), 21 in *Oryza sativa* (monocot), and 15 in *Arabidopsis thaliana* (eudicot), with the highest copy number being 39 in *Glycine max* (eudicot). Land plants contained higher copy numbers than algae, indicating that duplications of *5PTase* genes likely occurred after land plants diverged from green algae. The *5PTase* genes are also widespread among animals, from simple invertebrates, such as the *Caenorhabditis elegans*, to mammals, such as humans, with the gene copy number ranging from four to ten (Figure 2). 

To standardize the gene names, we adopted a common nomenclature system from previous studies based on the names of *Arabidopsis*, human, and yeast genes [11,16]. For yeast, human, and *Arabidopsis* genes with known functions or previous research, the published gene names were retained and used as a reference. Rice genes that were found to be orthologous to *Arabidopsis* were named after the *Arabidopsis* genes. Finally, recent paralogs were distinguished using a lower-case letter after the number.

### 3.2. Phylogenetic Classification of 5PTase Genes into Four Groups

**Figure 3:**
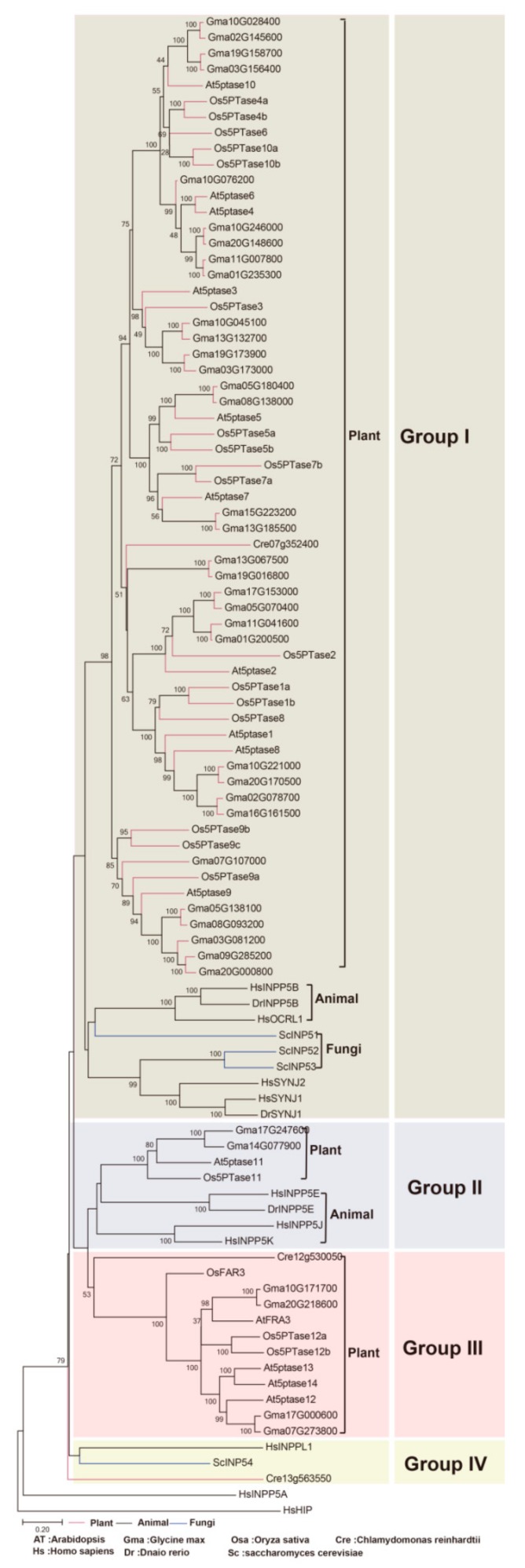
Phylogeny of representative *5PTase* genes from plants, animals, and fungi. Tree topology generated via maximum-likelihood (ML) using MAFFT (MSA tool that uses Fast Fourier Transforms) alignment is shown here. The protein sequences containing the conserved 5PTase domain were used for the alignment. At, *Arabidopsis*; Os, rice; Pp, *Physcomitrella patens*; Cre, *Chlamydomonas reinhardtii*; Hs, *Homo sapiens*; Dr, *Danio rerio*; Ce, *Caenorhabditis elegans*; Sc, *Saccharomyces cerevisiae.*

The overall sequence identity between different *5PTases* was very low (approx. 10%). In order to explore the evolutionary relationships of eukaryotic *5PTase* genes, we conducted phylogenetic analyses with amino acids containing the conserved 5PTase domain from representative species (Figure 3, Table S1, and Figure S2). Based on the phylogenetic analyses, the eukaryotic *5PTase* genes can be divided into four major groups. Notably, these four groups are inconsistent with the four types of animal 5PTases reported by Majerus et al. [2]. Among these, Group I contains members from fungi, plants, and animals; Group II contains genes from plants and animals; whereas Group III only contains genes from plants (Figure 3).

Group I contains genes from fungi, animals, and plants, indicating an early origin from the most recent common ancestor (MRCA) of the three kingdoms. More angiosperm *5PTase* genes were identified in Group I than in fungi and animal *5PTases*, indicating that this group of genes was expanded in angiosperms. Group II contains plant and animal *5PTase* genes, indicating that they originated before the separation of plants and animals. Four *Arabidopsis 5PTase* genes (*At5PTase12/13/14*, and *AtFAR3*) were identified in Group III, all of which contained several WD40 domains in their N-terminus [11]. Group IV also contains genes from fungi, animals, and plants, but their homologs were lost in angiosperm (Figure 3). In addition, angiosperm in Groups I and III had higher gene copy numbers compared with fungi and animals, suggesting the expansion and functional divergence of *5PTase* genes in angiosperms. 

### 3.3. Domain Features of 5PTase Proteins

**Figure 4:**
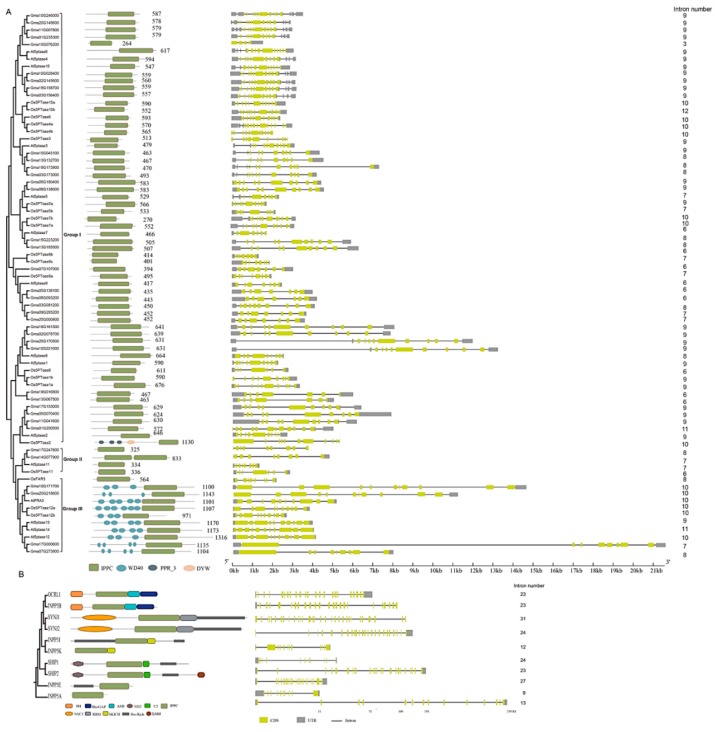
Phylogenetic relationships, exon/intron structure, and domain organization of *5PTase* genes in *Arabidopsis*, rice, soybean (**A**), and humans (**B**). The motif architecture is demonstrated as colored boxes. IPPC: inositol polyphosphate phosphatase catalytic domain; WD40: WD40/beta-transducin repeats domain; PPR_3: pentatricopeptide repeat domain; DYW: DYW_deaminase domain. The yellow boxes represent exons, the black lines represent introns, and the gray boxes refer to untranslated regions (UTRs).

To better comprehend the evolutionary relationship of *5PTase* genes, we analyzed the protein domain features of plant (Arabidopsis, rice, and soybean) and human 5PTases (Figure 4). The most closely related 5PTases display common motifs in plants and humans, respectively (Figure 4A,B). Four *Arabidopsis* 5PTase proteins (At5PTase12/13/14, FRA3), two rice 5PTase proteins (Os5PTase12a/12b), and four soybean 5PTase proteins (Gma10G171700, Gma20G218600, Gma17G000600, Gma07G273800) have multiple WD40 domains in their N-termini, indicating that these 5PTases might function by interacting with other proteins (Figure 4A). Interestingly, human 5PTases exhibited various combinations of different conserved motifs, including pleckstrin homology (PH), SH2, RRM, SAC1, SAM, and RhoGTPase-activating protein (RhoGAP), which determine protein subcellular localization and/or protein–protein interactions. These different motifs determine unique cellular and subcellular distributions and specialized functions of human 5PTases. For example, Synaptojanin 1 (SYNJ1) is a major neuronal 5-phosphatase [55,59], whereas OCRL1 is a major 5-phosphatase in fibroblasts [60,61].

### 3.4. Structural Analysis of 5PTase Family Genes

Intron loss and gain have relative importance in eukaryotic evolution [62]. The exon/intron organizations of different *5PTase* genes were examined for available genome sequences. Most plant members exhibit a similar exon/intron organization in terms of exon length and intron number, whereas there was a considerably more diverse number and length of introns in human *5PTase* genes (Figure 4). For instance, *5PTase* genes in plants have 6–12 introns; 6% (27/75) of the genes have nine introns, 20% (15/75) have ten, 2.6% (2/75) have 11, and 2.6% (2/75) have 12 introns (Figure 4A). Whereas human *5PTase* genes have 9–31 introns and are strikingly distinct in intron arrangement and number between different genes (Figure 4B). The great differences in exon/intron organization between plant and human 5PTases suggest that intron gain or loss may play an important role in the structural evolution of the 5PTase family.

### 3.5. Identification of Multiple Duplication Events in Land Plant 5PTases

**Figure 5:**
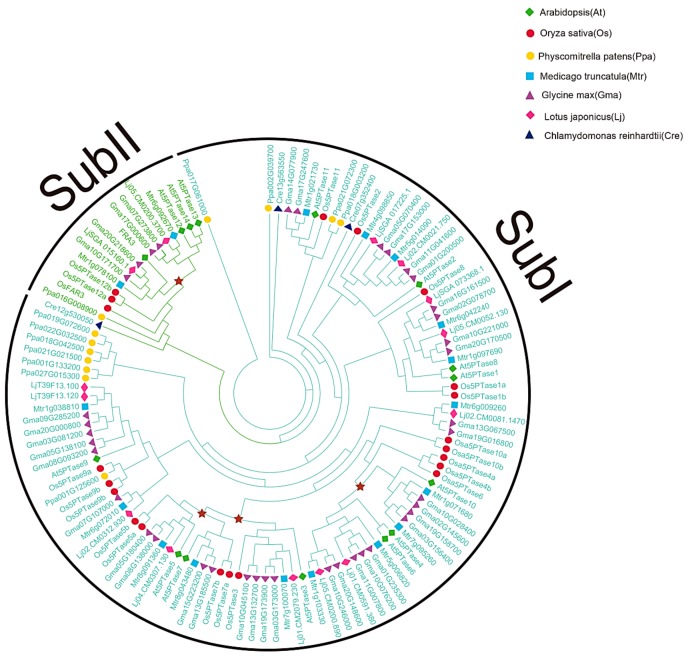
Phylogenetic analysis of plant *5PTase* genes. The tree topology of 5PTase protein sequences was constructed by Maximum Likelihood (ML) using the Jones, Taylor, and Thorton model among *Arabidospis*, rice, moss, *Medicago truncatula*, soybean, *Lotus japonicas,* and *Chalamydomonas reinhardtii*. The 5PTase protein sequences were aligned using MAFFT (MSA tool that uses Fast Fourier Transforms). The red stars represent major duplication events.

To further investigate the evolution and duplication events of the *5PTase* gene family, we conducted phylogenetic analyses in plants (Figure 5). *5PTase* genes were divided into two subgroups, with the corresponding proteins of most members in Subgroup II containing several WD40 domains (Figure 4). Within Subgroup I, there were three independent duplications in angiosperm and eudicots, suggesting that the duplication events likely occurred in the ancestor of a corresponding lineage (Figure 5). The duplication of eudicot *At5PTase10/6* occurred in the common ancestor of core eudicots, and the two duplications of *At5PTase3/5/7* occurred before the divergence of eudicots and monocots. Within Subgroup II, there was one independent duplication in core eudicots, suggesting that the duplication events likely occurred in the ancestor of core eudicots (Figure 5). In addition, 39 *5PTase* genes were identified in *Glycine max,* along with 16 soybean leaf pairs (Figure 5), demonstrating that more gene duplications occurred in soybean *5PTase*.

**Figure 6:**
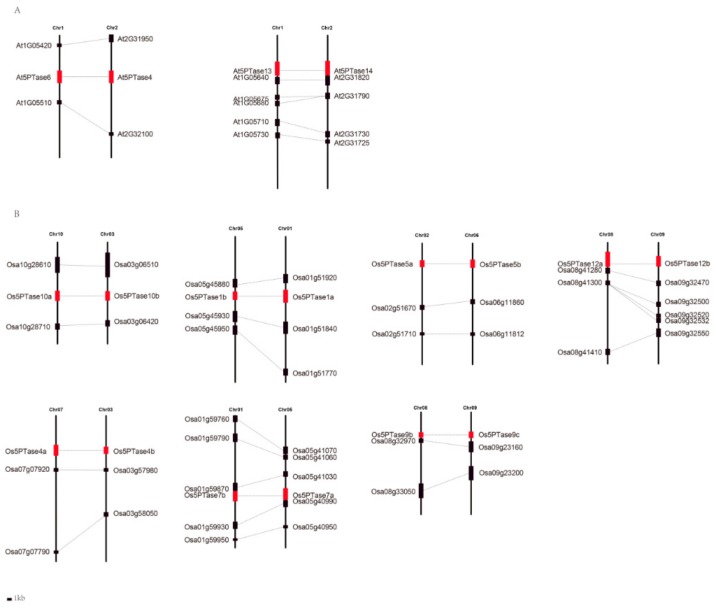
Collinearity analysis of duplicated *5PTase* gene pairs. At, *Arabidopsis thaliana*; Os, *Oryza sativa*. The syntenic paralogous genes are connected by lines.

WGDs are common in angiosperms, so in order to explore whether plant *5PTase* genes were caused by genome duplication, we searched for possible synteny in genomic regions containing the *5PTase* genes. Two and seven pairs of *Arabidopsis* and rice *5PTases*, respectively, were found in syntenic genomic regions, indicating that these multiple gene copies are the result of whole genome or segmental duplications (Figure 6, Table 1). To investigate the molecular evolution of *5PTase* genes, the ratio of non-synonymous to synonymous substitutions (Ka/Ks) was estimated (Table 1), which revealed that the Ka/Ks ratios varied from 0.06 to 1.87 among three differential species (Table 1). Most of the *5PTase* duplicates displayed lower Ka/Ks ratios (less than 1), indicating that these duplicates experienced strong purifying selection. The Ka/Ks ratio of *Os5PTase9b/9c* was 1.01, suggesting that these two *5PTase* genes were constrained by neutral evolution in rice, whereas the Ka/Ks ratio of *Os5PTase1a/1b* was 1.87, indicating that its evolution was driven by positive selection, thus allowing it to neo-functionalize. Soybean contains multiple gene duplicates and Ka/Ks ratios that range from 0.07 to 0.39, with none being greater than 1 (Table 1). 

### 3.6. Expression and Functional Divergence of 5PTase

**Figure 7:**
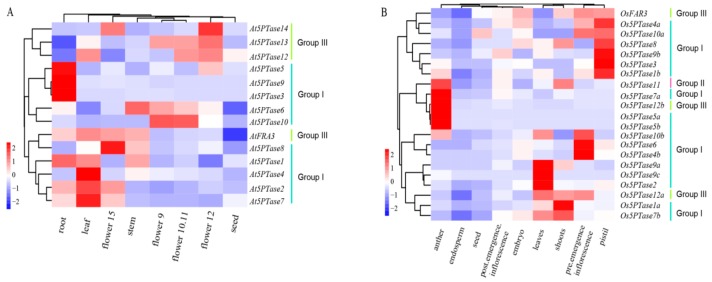
Developmental expression patterns of 5PTase family genes in *Arabidopsis* and rice. Expression profiles of (**A**) *At5PTases* and (**B**) *Os5PTase* in different developmental stages obtained from microarray data reported in the Genevestigator. Results are shown as heat maps in blue/white/red (low to high) that reflect the percentage of expression.

To investigate differences in the expression of *5PTase* genes, we analyzed the expression profiles of *At5PTases* and *Os5PTases* during plant development, based on the microarray data reported in the Genevestigator (Figure 7). Overall, all of the *At5PTase* and *Os5PTase* genes are expressed during the vegetative and reproductive development stages, and display strong organ specificity. In *Arabidopsis*, most Group I *5PTases* are mainly expressed in vegetative organs: *At5PTase3*, *At5PTase5*, and *At5PTase9* are expressed the highest in the root; *At5PTase2*, *At5PTase4*, and *At5PTase7* are mainly expressed in the leaf; whereas most of the Group III *5PTases* are mainly expressed in the reproductive organs (Figure 7A). In addition, differential expression patterns also exist among *5PTases* in the same group. For example, *At5PTase8*, one *5PTase* gene in Group I, is mainly expressed in old flowers but not in the vegetative organs. In rice, most *Os5PTases* are highly expressed in the reproductive organs, and other *Os5PTases* are mainly expressed in the leaves and shoots. Consistent with *At5PTase* expression, rice *5PTases* also display both similar and differential expression patterns among the same groups (Figure 7B). Apparently, *Os5PTase6*, *Os5PTase4b*, and *Os5PTase10b* are mainly expressed in early inflorescence; *Os5PTase11*, *Os5PTase7a*, *Os5PTase5a*, *Os5PTase5b*, and *Os5PTase12b* are only highly expressed in anthers; whereas, *Os5PTase1b*, *Os5PTase3*, *Os5PTase9b*, *Os5PTase8*, *Os5PTase4a*, and *Os5PTase10a* are only highly expressed in pistil. All of these results indicate both the conservation and diversification of expression among *5PTase* genes in *Arabidopsis* and rice.

The Ka/Ks ratios were less than 1 in soybean *5PTase* paralogs, suggesting that soybean *5PTase* genes have undergone strong negative selection pressure (Table 1). To gain insight into the potential developmental roles of *Gm5PTase* genes, the expression profiles of *Gm5PTase* genes were analyzed in 28 developmental stages/tissues, based on previous transcriptome data [54]. Overall, many genes displayed a distinct tissue-specific expression pattern, suggesting specific roles in particular stages of development (Figure S3). For example, seven *Gm5PTase* genes (*Gma03G081200*, *Gma20G000800*, *Gma09G285200*, *Gma08G093200*, *Gma19G173900*, *Gma05G070400*, and *Gma13G185500*) are mainly expressed in the root, and seven *Gm5PTase* genes (*Gma17G153000*, *Gma10G045100*, *Gma03G173000*, *Gma07G107000*, *Gma13G132700*, *Gma05G180400*, and *Gma08G138000*) are mainly expressed in flowers. These tissue-specific expression patterns are consistent with the expression patterns of *At5PTases* and *Os5PTases* (Figure 7). 

To further investigate the functional roles of *Gm5PTase* genes, we analyzed the transcriptomic datasets of soybean *5PTases* under different abiotic stresses, such as drought, cold, and high temperature (Li, Wang, and Yuan, unpublished data) (Figure S3). Many *Gm5PTase* genes showed specific induction under the cold, heat, and drought. For example, seven, nine, and seven *Gm5PTase* genes were induced under cold, drought, and heat stress, respectively (Figure S4), indicating that soybean *5PTase* genes might play important roles in stress responses. 

## 4. Discussion

### 4.1. 5PTases Genes Have Been Conserved during Speciation

Multiple *5PTase* genes were found in each of the plant, animal, and fungal genomes analyzed, including dicots, monocots, bryophytes, vertebrates, and invertebrates (Figure 2). This indicates that *5PTase* genes were present prior to the diversification of plants, animals, and fungi (Figure 3). In land plants, more *5PTase* genes were identified than in animals, indicating that the number of *5PTase* genes in plants underwent expansion. Based on the phylogenetic analyses, the *5PTases* diverged earlier during specification (Figure 3). In addition, the Ka/Ks ratio revealed that most plant 5PTase protein families underwent a process of purifying selection (Table 1), suggesting that the 5PTase protein families tended to be stable during the long evolutionary process.

Multiple gene copies were found for the plant *5PTases*, and many of them were localized in syntenic genomic regions, indicating that they resulted from whole genome duplications (WGDs) or segmental duplications, and in our study, 17 Gm5PTase paralogous pairs were identified in the soybean genome. In a previous study, the soybean genome was shown to have experienced two WGD events; one occurred 59 million years ago (mya) and the second occurred 13 mya [63,64]. The divergence period of *Gm5PTase* gene pairs ranged from 6.41 to 15.68 mya, indicating that these Gm5PTase pairs were formed by the most recent WGD, which also suggests that the WGD duplication was the main mechanism for the *5PTase gene* family expansion during the evolution of soybean. It has been previously reported that regulatory genes (i.e., protein kinases and transcription factors) and signaling genes are more likely to be retained after duplication events compared to the genome-wide average [65,66,67]. During cell growth, development, and stress responses, 5PTases are the key enzyme of the phosphatidylinositol (PI) pathway. They serve as molecular “hubs” during PI signaling and have, therefore, been conserved throughout evolution.

### 4.2. Functional Divergence in the Evolution of 5PTase Genes

Gene duplication provides the raw material for functional innovation [68]. 5PTase proteins are present in nearly all domains of life and display an ancient origin before the divergence of fungi, plants, and animals (Figure 3). This long evolutionary history has allowed a great deal of sequence divergence and resulted in low sequence identities between different subfamilies. Yet, the key functional domain of 5PTases remained constant in most members, with likely conservation of the original 5-phosphatases activity being the possible result of strong selection. 

**Figure 8:**
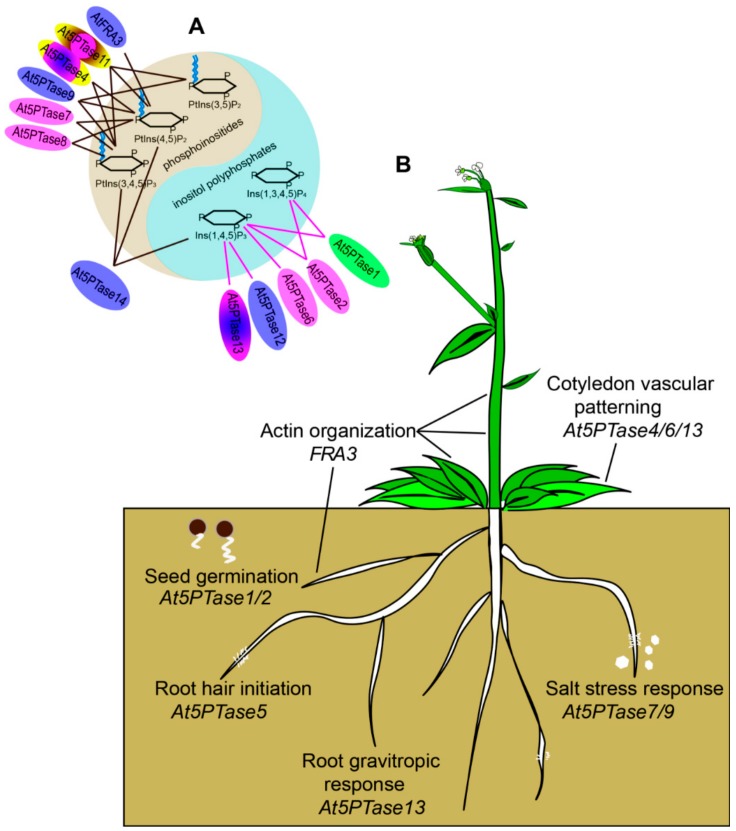
Functionally characterized *5PTase* genes from *Arabidopsis*. (**A**) Substrate specificity and protein localization of At5PTases. FRA3, 5PTase9, 5PTase12, and 5PTase14 were localized in the nucleus; 5PTase2, 5PTase6, 5PTase7, and 5PTase8 were localized in the cytoplasm; 5PTase1 was localized in chloroplasts; 5PTase13 was localized in the nucleus and cytoplasm; 5PTase11 was localized in the cytoplasm and plasma membrane; and 5PTase4 was localized in the nucleus, cytoplasm, and plasma membrane. Different localizations were displayed with different colors. 5PTase 1, 5PTase 2, 5PTase 6, 5PTase 12, and 5PTase 13 belong to Type I 5PTases and purple lines are used to connect them with the 5-phosphatase substrates. 5PTase 4, 5PTase 7, 5PTase 8, 5PTase 9, 5PTase 11, 5PTase14, and FRA3 belong to Type II 5PTases and black lines are used to connect them with 5-phosphatase substrates [2]. (**B**) 5PTases are crucial for multiple processes of plant growth, including seed germination [35], vascular patterning [36,37], root hair initiation [35], and salt stress [40,41].

Previous studies have shown that functional divergence could have occurred by changes in gene expression patterns or protein subcellular localizations [69,70,71]. Intron loss or gain is important in generating structural diversity and complexity, which further promotes variability [71,72]. Members of the *5PTase* pairs showed diversity in their transcription profile. As shown in Figure 7, AT5PTases and Os5PTases have shown diversity in different developmental stages: sub-cellular localization in the nucleus, chloroplast, cytoplasm, plasma membrane, and substrate specificity to inositol polyphosphates and phosphoinositides (Figure 8). In addition, the numbers of introns and the intron arrangement are strikingly distinct between plants and animals, which also contributes to the functional divergence and diversity of 5PTase family proteins during the evolutionary process in plants and animals. Therefore, the functional divergence of 5PTase has apparently arisen from protein characteristics (including sub-cellular localization, and substrate specificity), gene structural diversity (exon/intron structure), and expression patterns. These results indicate the functional divergence of 5PTases in plants and animals during their long-term evolutionary history.

## 5. Conclusions

Highly retained proportions of 5PTases were observed in plants and animals using phylogeny analysis. 5PTases in plants and animals play a complicated role in many growth and metabolic processes. Different 5PTases show complex and cross-linked substrate specificity (Figure 8). However, the function of each plant 5PTase and its molecular mechanisms are still unclear, and the interactions among 5PTases are still barely understood. Further studies will be carried out to explore the functions of *5PTase* genes in plants.

## Figures and Tables

**Table 1:** Divergence between *5PTase* gene pairs in plants.

Paralog Pair		Ka	Ks	Ka/Ks	Duplication Date (mya)	Duplication Type
*At5PTase4*	*At5PTase6*	0.06	1.01	0.06	82.75	WGD or segmental
*At5PTase13*	*At5PTase14*	0.16	0.70	0.22	57.08	WGD or segmental
*Os5PTase1a*	*Os5PTase1b*	0.41	0.22	1.87	18.03	WGD or segmental
*Os5PTase4a*	*Os5PTase4b*	0.13	1.06	0.12	87.07	WGD or segmental
*Os5PTase5a*	*Os5PTase5b*	0.21	2.17	0.10	178.04	WGD or segmental
*Os5PTase7a*	*Os5PTase7b*	0.20	2.10	0.09	172.52	WGD or segmental
*Os5PTase9b*	*Os5PTase9c*	0.32	0.32	1.01	26.23	WGD or segmental
*Os5PTase10a*	*Os5PTase10b*	0.12	1.16	0.11	95.09	WGD or segmental
*Os5PTase12a*	*Os5PTase12b*	0.15	0.80	0.19	65.88	WGD or segmental
*Gma11G041600*	*Gma01G200500*	0.03	0.17	0.20	14.25	WGD or segmental
*Gma17G153000*	*Gma05G070400*	0.03	0.12	0.27	9.97	WGD or segmental
*Gma02G078700*	*Gma16G161500*	0.03	0.08	0.34	6.41	WGD or segmental
*Gma10G221000*	*Gma20G170500*	0.02	0.13	0.19	10.76	WGD or segmental
*Gma20G148600*	*Gma10G246000*	0.01	0.07	0.14	6.11	WGD or segmental
*Gma11G007800*	*Gma10G076200*	0.01	0.13	0.07	10.46	WGD or segmental
*Gma10G028400*	*Gma02G145600*	0.02	0.12	0.15	9.95	WGD or segmental
*Gma19G158700*	*Gma03G156400*	0.02	0.11	0.17	9.20	WGD or segmental
*Gma15G223200*	*Gma13G185500*	0.02	0.09	0.18	7.57	WGD or segmental
*Gma05G180400*	*Gma08G138000*	0.02	0.08	0.22	6.49	WGD or segmental
*Gma19G173900*	*Gma03G173000*	0.04	0.13	0.30	10.85	WGD or segmental
*Gma10G045100*	*Gma13G132700*	0.03	0.14	0.21	11.49	WGD or segmental
*Gma20G000800*	*Gma03G081200*	0.08	0.19	0.39	15.68	WGD or segmental
*Gma05G138100*	*Gma08G093200*	0.02	0.08	0.28	6.42	WGD or segmental
*Gma13G067500*	*Gma19G016800*	0.01	0.12	0.11	9.68	WGD or segmental
*Gma17G000600*	*Gma07G273800*	0.03	0.09	0.29	7.70	WGD or segmental
*Gma10G171700*	*Gma20G218600*	0.02	0.09	0.21	7.18	WGD or segmental

## Supplementary Materials

The following are available online at https://www.mdpi.com/2073-4425/10/5/393/s1, Table S1. The amino acids of 5PTase genes, Figure S2. The sequence alignment of *5PTases*, Figure S3. Heatmap showing the expression of soybean *5PTases* in different tissues, Figure S4. Expression of soybean 5PTase genes under drought, cold, and heat treatment.
