# Supplementary material for: Expansion and Functional Divergence of Inositol Polyphosphate 5-Phosphatases in Angiosperms
Source: Genes (Basel). 2019 May 22;10(5):393. doi: 10.3390/genes10050393 (PMC6562803; doi:10.3390/genes10050393)
Supplement: Supplementary file 1 [file genes-10-00393-s001.zip › Table S1.docx]

Table S1

Figure 3 The amino acids of 5PTase genes..

| AT1G65580  （AtFAR3） | MEDRQNDQNDDVFSFFSPSFSAATPSTLFNRSAYSSSSSSGDDESQPSVDDSNKRIDYMIQFLDRRLSEDGNHDGIGDGNGSDSLPEFVGKCGESGIFKVPIRSAVHPNRPPSLDVRPHPLRETQIGRFLRTMTSTERQLWTGGEDGALRVWEFSELYGSGRGLEVEDTAPYKESLGNEFGSAAVVCMIGDEGSRVVWSGHRDGRIRCWRLRGDHGIEEALSWQAHRGPVLSIAISAYGDIWSGSEGGALKVWPWDGALGKSLSLKMEERHMAALAVERSYIDPRNMVSANGFANTLTSDVTFLVSDHTRARVWSASPLTFAIWDARTRDLIKVFNIDGQLENRPENSVYPDFGSEEEGKMKVTASKKEKAQSSLGFFQRSRNAIMGAADAVRRAATKGGFCDDSRKTEAIVISVDGMIWTGSSNGILMRWDGNGNCLQEFAYESSGILCMFTFCSRLWVGYSNGTVQVWDLEGKLLGGWVAHSGPVIKMAIGAGYLFTLANHGGIRGWNVTSPGPLDNVLRAELAGKEFLYSRIENLKILAGTWNVGEGRASDSLVSWLGCAATGVEIVVVGLQEVEMGAGVLAMSAAKETVGLEGSPLGQWWLDMIGKTLDEGSSFVRVGSRQLAGLLICVWVRHDLKPHVGDVDAAAVPCGFGRAIGNKGAVGVRLRMYDRVLCFVNCHFAAHLEAVNRRNADFDHVYRTMTFSRQSSSLNAGVAGASFGVTMPRGGNALGVNTIEARPELSEADMVIFLGDFNYRLDDITYDETRDFISQRCFDWLREKDQLHTEMEAGNVFQGMREAIIRFPPTYKFERHQAGLAGYDSGEKKRIPAWCDRILYRDNKKHLGAECSLDCPVVSSISQYDACMEVTDSDHKPVRCVFSVKIARVDESVRRQEYGNIINSNKKIKVLLGELSKVPETIVSTNNIILQNQDSTILRITNKSEKNIAFFKIICEGQSKIEEDGQAHDHRARGSFGFPQWLEVSPGTGTIKPNQIAEVSVHLEDFPTVEEFVDGVAQNSWCEDTRDKEVILVLVVHGRFSTETRKHRIRVRHCPRGGPAKNHFNDGTKTSGQINALHRSDYHQLSNTLDVVEQLKNLHSP |
| --- | --- |
| AT1G05630  (At5Pase13) | MDSLIIEEEDEEALATLVPVPPRRKTHSYSLQFDHKPHHQIRKHSLDEVPRSATLASEAVYFDSSDDEFSTGGNITENAADETNAGAEEYTIVNPPPNVGLGDDDTEPLPEFIGAGGGSGIFKVPVRAAVHPGRPPCLELRPHPLRETQTGRFLRNIACTETQLWAGQENGIRFWNLEDAYEAGCGIGGQVPRGDEDTAPFHESVTTSPTMCLVADQSNKLLWSGHKDGKIRAWKMDQSSVSHDDDDSDPFKERVSWLAHRGPVNSIVISSYGDMWSCSEGGVIKIWPWDTLEKSLLLKPEEKHMAALLVERSAIDLRSQVTVNGTCSISSSEVKFLLADSVRAKVWAVQSLSFSIWDARSKDLLKVLNVDGQVENRGDLPPIQDQQVDDEMKLKFFSASKREKPQGFLQRSRNAIMGAAGAVRRVATRSAGAFSEDTRKTEAIVLAVDGTIWTGSISGLIVQWDGNGNRLRDVNHHHRPVLCFCTFGDRIYVGYASGYIQVLDLDGKLISSWVSHNEPVIKLAAGGGFIFSLATHGGVRGWYVTSPGPLDNIIRTELSQKETLYARQDNVRILIGTWNVGQGRASHDALMSWLGSVTSDVGIVAVGLQEVEMGAGFLAMSAAKETVGLEGSAVGQWWIDAIGKALDEKNTFERMGSRQLAGLLISLWARKDIRTHVGDLDVAAVPCGFGRAIGNKGGVGLRIRVYDRIMCFVNCHLAAHLEAVNRRNADFNHIFRLMVFSRGQNLSNAAAGMVPYLFLSCSLGFSTYLFWLLYSSGLPWALSLAAGVSTSAYTTKSNTIPSTGAEEIKSDLAAADMVAFFGDFNYRLFGITYDEARDFISQRSFDWLRERDQLRAEMKVGKVFQGMREALITFPPTYKFERNRSGLGGYDSGEKKRIPAWCDRVIYRDTQSSPFSESNLQCPVVSSVIMYEACMDVTESDHKPVRCKFHATIAHVDKSVRRQELGKIIRSNEKILSIFEDLRFVPETSVSTNNIVLQSQDTVILTITNNSPTSQAIFNILCGGQAVVKDDGEDADYNPRGSFGLPRWLEVSPAAGIINPEGSVDVKVHHEDFYSMEEYVDGIPQNWWCEDTRDKEAILMVNIRGSCSTTLRSHSVKVRHCFSARVCLLENRPTNLTKNLGGSRRYPTDITRNGSTRPRTEDSVRRGKSR |
| AT1G05470  (At5PTase6) | MREEKSKTNKLAWSKKMVRKWFNIKSKTEEFQADDPSSAGIEVEHRSSFSAEKAPSTIKNTKTEKLSKNWEQQARQRRMNYENPRIIDVQNYSIFVATWNVAGRSPPSDLNLDEWLHSSAPADIYVLGFQEIVPLNAGNVLGAEDNGPAQKWLSLIRKTLNNRPGTSGTSGYHTPSPIPVPMAELDADFSGSTRQKNSTFFHRRSFQTPSSTWNDPSIPQPGLDRRFSVCDRVFFSHRPSDFDPSFRGSSSSHRPSDYSRRPSDYSRRPSDYSRRPSDYSRRPSDSRPSDYSRPSDYYSRPSDYSRPSDFSRSSDDDNGLGDSPSTVLYSPGSAANENGYRIPWNSSQYCLVASKQMVGVFLTIWVKSELREHVKNMKVSCVGRGLMGYLGNKGSISISMLLHQTSFCFVCTHLTSGQKEGDELKRNSDVMEILKKTRFPRVKSSEEEKSPENILQHDRVIWLGDLNYRIALSYRSAKALVEMQNWRALLENDQLRIEQKRGHVFKGWNEGKIYFPPTYKYSRNSDRYSGDDLHPKEKRRTPAWCDRILWFGEGLHQLSYVRGESRFSDHRPVYGIFCAEVESAHNRIKRTTSYSASRVQAEELLPYSRGYTELSFF |
| AT1G47510  (At5PTase11) | MPTMGNKNSMCGLKRFPNYKKSPIGSFAKNSSSHDGIKTIEAVNSCSFSRKADLCIRIITWNMNGNVSYEDLVELVGKERKFDLLVVGLQEAPKANVDQLLQTASSPTHELLGKAKLQSVQLYLFGPKNSHTLVKELKAERYSVGGCGGLIGRKKGAVAIRINYDDIKMVFISCHLSAHAKKVDQRNTELRHIANSLLPRDKRKRDLTVWLGDLNYRIQDVSNHPVRSLIQNHLQSVLVSKDQLLQEAERGEIFKGYSEGTLGFKPTYKYNVGSSDYDTSHKIRVPAWTDRILFKIQDTDNIQATLHSYDSIDQVYGSDHKPVKADLCLKWVNS |
| AT1G71710  (At5PTase8) | MSPVEPAGIMKKSHRQKSQRLWAKLVMRKWLNISGRDPEYGADTDNESENEDAREDNDDSSSDEEGGSGSRGRESKVYENAEDAIAAASAVVDAAAAAAEFISNDAPMKLRRRNSETLRAQYINNKEIRVCVGTWNVGGISPPSDLDIDDWIEINQPADIYVLGLQEIVPLNAGNILGAEDDRPVAKWEEVIREALNRVRPKLSGVKSYSDPPSPGRFKPFEETHDIIEEEVAFESDSDAGVEIHPIDEEEEEETDRLWALKHDGGVIGEVKTLVDPNTGLPVVEIKRQFSIPKKLDRQLCLRADSFKGISDDDSTQTGMKTINRMLSGKERIGLSWPEPPLNMLGPCVLDRQPSIKTVKSLKTAKSFKAYSSFKSVAGNNNGIPPEVLALAEMDLKLLMERKRRPAYVRLVSKQMVGILLTIWVKRSLRKHIQNVRVSTVGVGVMGYIGNKGAVSVSMSINQTFFCFINTHLTAGEREVDQIKRNADVHEIHKRTVFHSVSALGLPKLIYDHERIIWLGDLNYRLSSSYEKTRDLISKREWSKLLEYDQLVKEYRKGRAFDGWSEGTLHFPPTYKYQANSDEYTANDGKAPKRTPAWCDRVLSYGKGMRLVHYRRTEQKFSDHRPVTAIYMAEVEVFSARKLQRALTFTDAEIEDEGLVAVLV |
| AT1G34120  (At5PTase1) | MAEVRSRSRRTESNWATICCSAFSCLQLYWARIVLRKWFNVSASESDYSADSDDDYEDRSQEFDPISSGVTNPRVDTDGNVIYRPKLRRRNSETFRMQYIDTKAIRICAGTWNVGGRVPSSDLDIDGWLDTLEPADIYVLGLQEIVPLNAGNIFGMEDDQPALEWENLIRDALNRVQPRKLKIKSHSDPPSPSKFKQPEEVPYSVEDMFVETSHDACDGISSMDNKLNSVESTDVPIVSEDSLTNIDVLGSTNDNASCLPIQEYLQRQFSTPNTPDRSLSMQINSDSKREERFSYTERVGLSWPEPPLRLLNQYVSERRGSFKSVNLTITNLRKPSYVRIVSKQMVGVFLTIWVRRNLRKHISNLCVSTVGVGIMGYIGNKGSVSVSMSIYQTPFCFLCTHLSSGEKDTDQEKRNDDVREIHRRTQFLPHSLNANELPRSICNHERIIWLGDLNYRINLSYEKTHELIARKEWQRLVEYDQLSREMTKGNLFEGWSEGTLDFAPTYKYEIDSENYIGDDPESGKRRPAWCDRIIWNGKGMKLFNYRRNEIKLSDHRPVTATFLAEVEVLSPRKLQHALTLTYAEIQGLDA |
| AT2G43900  (At5PTase12) | MDIINNNHRDENDDDEEEALSAMSSVPPPRKIHSYSHQLRATGQKGHHRQRQHSLDDIPKITEIVSGCGISGDSSDDEFYPYATTTNSSSFPFTGGDTGDSDDYLHQPEIGEDFQPLPEFVGSGGGVGMFKVPTRSPLHSARPPCLELRPHPLKETQVGRFLRNIACTETQLWAGQESGVRFWNFDDAFEPGCGLSGRVQRGDEDAAPFQESASTSPTTCLMVDNGNRLVWSGHKDGKIRSWKMDYVLDDGDDSPFKEGLAWQAHKGPVNSVIMSSYGDLWSCSEGGVIKIWTWESMEKSLSLRLEEKHMAALLVERSGIDLRAQVTVNGTCNISSSEVKCLLADNVRSKVWAAQLQTFSLWDGRTKELLKVFNSEGQTENRVDMPLGQDQPAAEDEMKAKIASTSKKEKPHGFLQRSRNAIMGAADAVRRVATRGGGAYEDAKRTEAMVLAGDGMIWTGCTNGLLIQWDGNGNRLQDFRHHQCAVLCFCTFGERIYIGYVSGHIQIIDLEGNLIAGWVAHNNAVIKMAAADGYIFSLATHGGIRGWPVISPGPLDGIIRSELAEKERTYAQTDSVRILTGSWNVGQGKASHDALMSWLGSVASDVGILVVGLQEVEMGAGFLAMSAAKESVGGNEGSTIGQYWIDTIGKTLDEKAVFERMGSRQLAGLLISLWVRKNLRTHVGDIDVAAVPCGFGRAIGNKGGVGLRIRVFDRIMCFINCHLAAHLEAVNRRNADFDHIYKTMSFTRSSNAHNAPAAGVSTGSHTTKSANNANVNTEETKQDLAEADMVVFFGDFNYRLFGISYDEARDFVSQRSFDWLREKDQLRAEMKAGRVFQGMREAIITFPPTYKFERHRPGLGGYDSGEKKRIPAWCDRVIFRDTRTSPESECSLDCPVVASIMLYDACMDVTESDHKPVRCKFHVKIEHVDRSVRRQEFGRIIKTNEKVRALLNDLRYVPETIVSSNSIVLQNQDTFVLRITNKCVKENAVFRILCEGQSTVREDEDTLELHPLGSFGFPRWLEVMPAAGTIKPDSSVEVSVHHEEFHTLEEFVDGIPQNWWCEDTRDKEAILVVNVQGGCSTETVCHRVHVRHCFSAKNLRIDSNPSNSKSQSLKKNEGDSNSKSSKKSDGDSNSKSSKKSDGDSNSKSSKKSDGDSNSKSSKKSDGDSNSKSSKKSDGDSNSKSSKKSDGDSNSKSSKKSDGDSCSKSQKKSDGDTNSKSQKKGDGDSSSKSHKKNDGDSSSKSHKKNDGDSSSKSHKKSDGDSSSKSHKKSEGDSSSKSHKKNDGDSSSSYKSQSGKKNSNSSTVEESRNNHNKR |
| AT2G37440  (At5PTase3) | MGKILKSKSSWPRTVVRKWLNLRSGAYEFHSDYPVKGMEPRRKSCSDMIVPENFQGWLGQGNGDLKHSTGEQHVTRVDDKLDLKMFVGTWNVGGKSPHEGLDLKDWLKSPADADIYVLGFQEIVPLNAGNVLGAEDNGPAAKWLSLIREALNNTNNLSPNELEHTKSSQQPRFSFSGLSDDTPIPCNSTPPRGYSLAASKQMVGIFLCVWVRDDLRKRITNLKVSCVGRGIMGYLGNKGSVSISMSLHETSLCFVCTHLTSGEKEGDELRRNLDVTEIFKRTRFSRSSKDSRPETIMDHDKVIWLGDLNYRLRASSDLHEQLRNHDWESLLEKDQLKIEQRAGRIFKGWEEGKIYFAPTYKYRINSDNYVVQTEKSKEKRRTPAWCDRILWKGDGMKQLWYVRGESKFSDHRPVQSLFSVHIDLKNQSNRKTKPVNQNHRPNPVLTYTCHGKVQAEEILLLTRAQSCIDTLPRLISSAS |
| AT2G32010  (At5PTase4) | MRDDKTKKSKLSWSKKMVRKWFNIKSKTEKFQADVSLPQGVEVEHRNSFSEREPCTIKKSKTEKLNKNWEQQARQRKMNYENPRIIDVQNHSIFVATWNVAGRSPPEDLNLDEWLHSSAPADIYVLGFQEIVPLNAGNVLGAEDNGPAKKWHSLIRKTLNNLPGASSACHTPSPIPVPIAEIDADFSGSSRQKNETFFNRRSFQTPSVWSMEENDPSISQPRLDRRFSVCDRVFFSHRPSDFDPSFRCGHRPSDYSRRPSDYSRPSDYYSRPSNYSRPSDVSRWGSSDDDNGPGDSPSTFLNSPGSFLGSAANENGYRTPWNSSQYCLVASKQMVGIFLTIWVKSELREHVKNMKVSCVGRGLMGYLGNKGSISISMLLHQTSFCFVCTHLTSGQKEGDELRRNSDVMEILKKTRFPRVQSSADEKSPENILQHDRVIWLGDLNYRIALSYRSAKALVEMQNWRALLENDQLRIEQKRGHVFKGWNEGKIYFPPTYKYSNNSDRYAGGDLHPKEKRRTPAWCDRILWHGEGLHQLSYVRGESRFSDHRPVYGIFSAEVESNHKRSKRTNSHSTARVEAEELLPYARGYTELTFF |
| AT2G01900  (At5PTase9) | MWPRLVANKILRKSLGSNNFVADFPPNTDQKLIEASGLADERSKSILHNQHKTTLLNYKVFVSTWNVGGIVPDDGLDMEDLLETHKTPCDIYVLGFQEVVPLRASNVLGSDNNKVSTKWNSLIRDALNKRARPHRDEDLSESKGINGISQDFRCIISKQMVGILITVWVRGDLWPYIRYPSVSCVGCGIMGCLGNKGSVSVRFQLHETTFCFVCSHLASGGRDRDERQRNSDVNEILARSSFPRGSSLDLPKKILDHDRVIFLGDLNYRISLPEEKTRLLVESKKWNILLENDQLRMEIMNGQIFRGWQEGIVKFAPTYKYVPNSDLYYGCITYKKDEKKRAPAWCDRIIWYGNGLKQHEYTRGETKISDHRPVKAIFTTEITVTRRGKKIRNFFFSDRFEERIGDIDSKDYSWIST |
| AT2G31830  (At5PTase14) | MDSVIIEPDEREALASLVPAHPLPPRKTHSYVEQCEQKPHHPIRKYSLDEGSRSVTSDSEAVYFDSSDGEFSTEGVAIVDGRTSGERGNGEECGFVTPPSKPASQGGGNDGGREDDIESLPEFIGAGGGLDVFKVPVRAAVNPGRPPCLELRPHPLRETQTGKFLRNIACTESQLWAGQENGVRFWNLEEAYEVGCGLGGQVRRGDEDTAPFHESVPTSPALCLLVDHGNRLVWTGHKDGKIRAWKMNQPNTTTADDSKPFKERLSWQAHRGPVNYIVISSYGDMWSCSDGGVIKIWTLDSLEKSLVLKLEEKHMAALLVERSGIDLRSQVTVNGTCSISSSDVKFLLVDTVKAKVWAVQHLSFSLWDAQNKELLKVFNIDGQVENRVDMPPTQGQQVEDTKAKFFSAPKKEKSQGFLQRSRHAIMGAAGAVRRAATRSAGAFAEDTRKVEAIAIAADGSIWTGSMNGVIAQWDGNGSRLREVNHHQQAVLCFCTFGDRIYVGYSSGYIQVLDLGGKLIASWVSHNEPVIKLAAGGGFIFSLATHGGVRGWYVTSPGPLDSLIRTELSQKEMAYARQDSVKILIGTWNVGEGRASRGALVSWLGSAVSDVGIVAIGLQEVDMGAGFLAMSTAKETVGVEGSAVGQWWLDAIGNALDERNTFERMGSRQLAGLLISLWVRKSIRTHVGDLDVAAVPCGFGRAIGNKGGVGLRIRVYDRIMCFVNCHLAAHLEAVTRRNADFNHIYRSMVFSKGQSVYTAAAAGASTSAQALKNNPNTNNSTEEEKSHLASADLVAFFGDFNYRLFGITYDEARDFISHRSFDWLREKDQLRQEMNEGKVFQGMREALITFPPTYKFEKNKPGLGGYDSGEKKRIPAWCDRVIYRDNQSISYTECSLKCPVVSSTIMYEACMDVTESDHKPVRCKLHANIAHTDKSVRRQELGKIVKSNEKLRAMFEELKSVPETSVSTNNILLHSQDTFIFTIRNTSNSSRAIFNIVCKGQTLVREDGEEPDNHSRGTFGLPRWLEVSPGAGIIKPDASLQVKVHHEDSHNSEEFIDGIQQNSLSEESSDKEVTLIIIVQGSCSTRTISHSIKVRHCSSAAKSLSLVHSKTTTMTKNLEGSTRYQTDANRDRRSSRTVKPSKEMHPVIVSSFSFLNSSDIRKGQIALRLFFRVQ |
| AT3G63240  (At5PTase10) | MGDGNLKKSKLSWPKTLVKKWLNIKSKSEDFHADDLDRGEGGGDWRNNVIEREEACSVRKSKTETRSKRNSGRARRNKLDVDPPLDHLRVFTATWNVAGKSPPSYLNLDDWLHTSPPSDIYVLGFQEIVPLNAGNVLGTEDNGPARKWVSLIRRTLNSLPGGSCQTPSPVPHPVAELDSDFEGDSAAGANSLFYHRSRSMRMDASASSLPQQFDRRFSVCDRFMLGDTPDDFYDQSFRYCSSEDEPADSPCHDHYSPVSRTGSFVADDRDKGRDKSKYCLVASKQMVGIFLTVWVKSDLRDSVNNLKVSCVGRGLMGYLGNKGSISISMSVHQTSFCFVCSHLTSGQKEGDELRRNSDVLEILRKTRFPRVNNAGDDKSPQMISEHDRVIWLGDLNYRIALSYRSAKALVEMRDWRALLEKDQLRIEQRKGCVFEGWKEGTIYFPPTYKYSNNSDIYAGDDRLPKAKRRTPAWCDRILWHGSGISQLSYVRGESRFSDHRPVYSLFSVEIESAYRNRIKKSSSYTSSRIEVEELLPQRYGYSELNPY |
| AT4G18010  (At5PTase2) | MKTRRGKRPERFWPSIVMNKWLNRKPKVYDFSEDEIDTEPESEDDVCSVKDVPNVHCVTDEDSHNGRRGSEADHGNNISDGGVSVRGGYQRKHRRGKSETLRAQYINTKDIKVTVATWNVAGKRPSDDLEIEDWLSTDNPSDIYIIGFQEVVPLNAGNVFGAEDRGPIPKWESIIRRTLNKSNKESVYDQSPSCNNNALHRSHSAPSSPILAQEANSIISHVMVENLVADHSLDLATNEFIDAATALPSLEPQRNPNMDWPELALDSNPQIVGSEGKLRRVFSSNATLGFKLPENPSGASRFASEARQLKRSRSFETLNLSWNDIKEEIDNRSSSSSEAEEAAKIMHDDSSDGDSSSQDEEDGDKIRNSYGLPEDLVEECRKVKDSQKYVRIVSKQMVGIYVSVWIRRRLRRHVNNLKVSPVGVGLMGYMGNKGSVSISMTLYQSRMCFVCSHLTSGHKDGAEQRRNADVYEIIRRTRFASVLDTDQPRTIPCHDQVFWFGDLNYRLNMSDGEVRKLVSQKRWDELKNSDQLIRELRRGHVFDGWREGPIKFPPTYKYEFDSDRYAGENLREPEKKRAPAWCDRILWLGKGIRQECYKRSEIRMSDHRPVTSIFNVGVEVFDHRKLQRALHVNNAAASAVHPEPSF |
| AT5G65090  (At5PTase5) | MNNRGNNDDLDHHYGVFNDFERRMTSRKKSVLDNTSPMIWKTVSERKSSPGIEGLNLSSFDRPMAPTTEIRELRVFLATWNVGGRTPNNDLNLEDFLLVEGTADLYICGFQEIVPLSAGNVLVVEDNEPAAKWLALISQALNKPKQESVYSNAAYSASRTTTCSSSSCGSEESRAPSSLSFFQRPNLKVLSRNYRVDSSLLKTCNCPVIDTSVGWEARRSKRFSDPSTDSSNNVEPENFRVHENFLFDDVPATTKMPGQMSYRLIASKQMVGLFLSVWARRELIPHISHLRLDSVGRGIMGRLGNKGCIAISMSLHQTSFCFVCSHLASGEKEGDELRRNADVAEILKHTQFPKLTKNPNCHAPERIIDHDRVLWLGDLNYRVALTYEETRVLLEDNDWDTLLERDQLNMERGAGRVFSGFQEGQIFFAPTYKYSQNSDAYAGEMTKSKKKRRTPAWCDRILWKGEGIEQLSYIRGESRFSDHRPVCAIFAVEVDVKSLNKGRFRKGYSCAAVRLVEDVAIPQRHSFYD |
| AT5G04980  (At5PTase7) | MSDPLYIFNRSQSEIVVESLLASSNLRSSMPTQQIQSLRVFVATWNVGGKSPHSGLNLDALLHVHSEFDVYVLGFQEIVPLNAGNVLVLGDNEPAAKWLAMINQSLNKSSSSSGGRLSPKTPSFGAGSMFFAKPSLKKISESFRTECRRKLKICNCSTFSEDIVRKYGRESCFRCPEGLVNQSGVLSDDEEDEDDDDDDEDEDEGGGKVASLVSNQMTMKYGLVASKQMVGIFLTVWMRKELIQHVSHLRISSVTRGIMGCLGNKGCIAVSLQLYKTSFCFICSHLASGEREGDERRRNLDVIEILKNTSFPRICRTSFTRVPDRITKHDRVIWLGDLNYRIALSYSETKTLLDKNAWDTLLNKDQLKIERDAGRVFKGWHEGKIFFAPTYKYSYNSDAYAGDTSKEKKNKRRTPAWCDRILWHGDGIRQLSYVRGESRFSDHRPVCSVFVVDVEVCEGKTGTRRQ |
| Osa10g28660  (Os5PTase10a) | MRDGAKKSKLSWSKSLVLKWFNIRGKSYDFHGDDAAAAFGRRGGGGEDEWRSSSFSRRESCTVKKSRTERASRRSHERSRRSKIDLDAAEATVTLDYRIFVATWNVGGRAPPGSLSLDDWLRTSPPADIYVLGFQEIVPLNAGNVLGAEDNGPARKWVSLVRRTLNSLAGTGGGGGGGGGGGMRTPSPAPDPVVEMDDDFEGSSSRQNNPAAFFHRRSFNAGLSRSLRMDGDILGGGGGAQPRLERRYSVNDRVMYGSRPSDYEANCRWGHPSDDGEIDDGGGESPSAVFSPMSYGYGAPPYMEESNGGAAHSRYCLVASKQMVGLFLMVWARREIKSDIRNLKVSCVGRGLMGYLGNKGSISVSMLLHQTSFCFVCSHLTSGQKDGDEHRRNSDVMEILRKTRFPMVYGQYERSPETILEHDRIIWLGDLNYRIALSYRSVKALVEMRNWKALLEKDQLRSEQRGGRVFPGWNEGRIYFPPTYKYSNNSDRYAGDDMNQKEKRRTPAWCDRILWYGRGLSQLSYVRGESRFSDHRPVYSMFSAEVESINHSRIQKMSCSSSQLDIEELLPYSYGYTDINPYGYTDLNFY* |
| Osa08g41270  (Os5PTase12a) | MDSDDEAAAAAMAARARETLRKSASSSSSSPYARSTDDGPVASASCDARLERCCREVGAAVAVVEEPERVVSGGGALPEFVGEGGGEGIYRVPLRAAMHPGRPPPLEVRPHPLRETQVGSFLRALACEPRRRQLWAGSESGVVWGLDDVFAAAGCGARRGDEESAPFRESVPVPPVLCVEADASNALVWTGHKDGRIMSWRMDLAAGSDDDDAPLFREALTWQAHSRTPVLSMVITSYGEIWSGSEGGVIKAWPWDVIAKSLSLMPEEKHVAALRIERSYIDLRNNAAAGNISSFPAADVKHMLADHSRAKVWCLTSMAFAVWDARTRELLKVFGMDGQIESARLEAPVMPEQFIEEEIKAKPVKKDKPQSSFNFFQKSRNALMGAAGAVRRVATKGTFVEDNRRTEAVVQAMNGTVWSGCTDGLIIMWDGNGNRLQEFQHHCSSVQCMKALGERVWVGYASGIIQVMDVEGNLLAEWTGHSCPVIQMAIGGSYVFTLAHHGGIRGWPLASPGPLDDILRTELSNRELSYRRLVNIKMLVGTWNVGQEKASYESLMSWLGRAFFDVDLVVVGLQEVEMGAGVLAMAAAKESVGLEGSANGQWWIDNIGRTLDEGISFHRVGSRQLAGLLIAAWARKDLKPHVGDVDAAAVPCGFGRAIGNKGGVGLRIRVYDRRICFVNNHFAAHLENVSRRNADFDHIYRTMTFNKPHGSAASATSVQLHKTVNANGNQVDEDIPEMAEADMVVFLGDFNYRLYGITYDEARDMVSQRSFDWLKERDQLQAEMRAGKVFQGMREGLIRFPPTYKFQRHLPGLAGYDSGEKKRIPAWCDRILYRDSRDVLTAECSLECPVVAKITSYEACMGVTDSDHKPVRCAFSVDIARVDEFTRRQEYGKILQSDKRLHSLLRESHFVPDTIISTNNIILENQEHVVLRITNDCQRNKAAFEILCESQSISKQDGTKSEFPPRASFGLPLWLEVEPSVGLIEPGQTMEVTVHHEDYYTQEVFVNGVLQNCWCEVTRDKEAVLLVNVTGSTSTETITHRINVRHCCSTISASPPINPPSITTPSVDVLSGEASTRSSKKNPLNYLQRSDFKPFGSSEVHDLCPL* |
| Osa08g32960  (Os5PTase9b) | MGDEKHPSSKLSEILRPGKLLHRRRRLVSEFADVGREDALHESDTVKYRVFAGTWNVAGVAPPDDLDLGDWLDAKADSYDIYVLGLQEIVPLNTRNVLGPTRSSAAMKWNSLIGDALNRFTTGRRRRDDDDDEGAQRHGQQQQPFRCVVSEQMVGIFVSVWARSGLRRHVRHAAASCVGAGVLGRLGNKGAVSVRFLLHGTSFCFVCCHLASGGKDGDAQLRNADAADILSRTTFRRRTTAAASPAPEELPLPRKILDHDRVVLLGDLNYRIAMDDAEARLLVRAGKWSMLLENDELLLELAEGGSFDGWREGLVTFSPTYKYHLNSDMFYWSIDAAAGGGDKQQQRAPAWCDRILWRGKGMRQASYERCGGYRISDHRPVRALFDAVCELAGGGVGVEHSAAGIASFGHVPLL* |
| Osa07g07950  (Os5PTase4a) | MREESAKKSKLSWSKSLVRKWFNIRTKAQDFHADSETTTQGRDGGGGAGGRASFSASSASTSSAKKSRTDRSSSKRSADRVRRGRNDFDLARLTEVQDYRIFAATWNVGGKSPPRGLNLDEWLHSSPPADIYVLGFQEIVPLNAGNVLGTEDNIPAKKWVSLIRRTLNKNPGASGSGVYHTPSPVLNPVVELEADFEASARRQENYSFFHRRSFHNLSRSLRMDADYMFPQPKLDRRFSVCDPVSLGGRPSDFDGNLRWLGSPDEENIDEELSNAAQCSPLPYSCNTTAPTEANDEQPNGSRYCLVASKQMVGIFLTVWVRNEIRDDVRNLKVSCVGRGLMGYLGNKGSISISMSLHQTSFCFICCHLTSGEKEGDELRRNSDVMEILRKTRFPRVRGANDVKSPETILEHDRIIWLGDLNYRIALSYCSARALVEMHNWKQLLEKDQLRIQQRYGRVFQGWKEGRIYFPPTYKYSFNSDRYAGEGMHPKEKRRTPAWCDRILWYGNGLNQLCYVRGESRFSDHRPVYSIFMAEVEIVHHRRKNMGYFSSRIEVEELLPHSQSYREINFY* |
| Osa01g08780  (Os5PTase8) | MVVQKNQRKPGEASWPKVVLKKWLNLKSKDSEFNADEEDDDDGSDVDEQENCGCDGGEERRRADGDLADENVEGGAPYRLRRRNSETLRAQYINTKELKLCVGTWNAAGKVPPGDLDIADWLGAGAGEPADVYVLGFQEVVPLNAGNVFGAEDARPAQAWEELIRSALRRARPPASSRPRYKCYSHPPSPSRGDATAAAAATDDDELFPGTDTDTDTNTDDDSLFSSPAESEQQNVAATPRRLTRLNHFTAAADAAAAMDDSGDEHQQRTLLKTLSRSDRVGLAWPEQPLDLLAKHATATASTTMPSSRSFRTYNSFRPSRAAAAADQSNDDLAMIADLVMDLAAARKRRSPYVRIVSKQMVGVFLTVWVRRGLRRCVHNVGVSTVGVGAMGYIGNKGAVSVSMSVYQTMFCFVCTHLAAGEKPADLHKRNADVQEIHRRTHFAGVGMPRNIYDHERIFWLGDLNYRIDVAYERAHELISTMDWTQLAEKDQLKRELRKGRAFDGWTEGVLEFAPTYKYELNSAKYIGDDQRGGRRTPAWCDRILSFGKGVKLMSYGRAELTMSDHRPVVATYAAEVEVFSSRKLQRALTLTDAEVEAGTVVAVPDHLAGF* |
| Osa01g59880  (Os5PTase7b) | MRLSHFLAQTPNNTTTEPVRIFVATWNVGGKAPTAELNLDDFLPPDDHSDIYVLGFQEIVPLNAGNVLVIEDNEPAARWLVLINQALNRPAETNANVFQNEPSPSVDSSVSRASSSLDTSFSDLAKTSSSSTIFQKSNLKSIRKSFMPVHRKRLKACNCPVEMAKSSYRDACFGCPKAYAYEIDSSEEDEREEKKGQSRDSNGSVRSEVISPPTARDELKYNLIACKQMVGIFVMVWVKKELVQHIGHLRTSCIGRGILGCLGNKVRSKA* |
| Osa01g51890  (Os5PTase1a) | MAGASSTSASARATPPARSLPPLGASGSQQEPAATASHHAAGAGASSRPMRRKGRKQKQLWPKTVLRKWLNIRSPESDFSADEGEATGDDDTDSEFEYEEMCHWERQLYDEERRLRGLGAETIDSQMEGAPYKLNRRRKSETLRAQYIDIKELRVCVGTWNVAGRLPPDDLDIQDWLDMEEPADIYVLGFQEIVPLNAGNIFGAEDNRPVAMWEHIIRETLNKISPDKPKYKCHSDPPSPSRFKPSDDVEDELVSESDSESGGEVHPWNEQDFTVDDDSVHSNKYEHSTSGPTETTVNGNNFSRVPSMKIFDRSHNLSFKDYVSSLEEPIHQKMLTKTLSYSERLGMIWPEQPLDILAQRLPDSTKPFISEKALRSCLSFKSAHGDSNAFPDDCLVHDFNIKSALVKTKRPYFVRIISKQMVGVFISIWVRRSLRKHIQNLKVSTVGVGAMGYIGNKGSIAVSMSIYQTLFCFICCHLTSGEKDGDELKRNADVQEIHRRTIFNPVSRVSMPKTIYDHERIIWLGDLNYRINLSYEKTHEFISMKDWNGLFQNDQLKREFKKGHLFDGWTEGVISFPPTYKYKVNSEKYTSDEPKSGRRTPAWCDRILSFGKGMRLQAYRTVDIRLSDHRPVTAVYTSDVEVFCPKKLQRALTFTDAEVEDQFSFEEESTSGIFSF* |
| Osa03g06460  (Os5PTase10b) | MRDGSNTTKKSKLSWSKSLVRKWFNIRSKAHDFHADDVAAIGRRGGDDEWRGSSFTRREPSTVKKSKTERSSRRSHERSRRGKIDLDAAEATVTLDYNRIFVATWNVGGRSPPNTMSLEDWLHAAPPADIYVLGFQEIVPLNAGNVLGTEDNGPARRWVSLVRRTLNNLPGTSGNGSFRTPSPAPDPVVEMDDDFEGLSSRQNNASFFHRRSFQAGLSRSLRMEGDILAPQPRLERRYSVCDRAIYGRRPSDYEATCRWGGSSDDENNTGESPSTVYSPMSYGYDIRDDIRNLKVSCVGRGLMGYLGNKGSISISMSLHQTSFCFVCSHLTSGQKDGDEMRRNSDVLEILRKTRFPMVYGQYERSPETILEHERIIWLGDLNYRIALSYRSVKALVEMRNWKALLEKDQLRIEQRGGRVFVGWNEGKIYFPPTYKYSNNSDKYAGDDMNQKEKKRTPAWCDRILWYGRGLSQLSYVRGESRFSDHRPVYSVFSAEAALLMEIRGPSEQVHIECSKIPEQHIAHLQKFTKHLNSSQDRFIVTKPRYGPCFTEP* |
| Osa03g13520  (Os5PTase9a) | MLENQRQAEVLWPRLVANKLFRKPSGSHAFVADFPMAVDDDFDGEAVPAAVESFDDDGCSPDADACRSVKRPRPRPQQRASNKTLKYRLFASTWNVGGVAPPDDLDLSDWLDTRNAAYDIYVLGFQEVVPLSARNVLGADKKRVGMRWNELVRAALNRSSPSAPNSSRDQREAKGTGGGAAAAAAGGGEIKQQAAQQKVHPVRGGIGGGGGELACRDYRCVVSKQMVGILLTVWVRADLARFVRRASVSCVGCGVMGCLGNKGAVSVRFWLHDTSFCVACCHLASGGRDGDEAHRNADATEILSRTTFPRGHSLNLPQKILDHDRVILLGDLNYRISLPEAKTRLLVERQDWKTLLENDQLRSEVESEGGAFHGWNEGAIAFSPTYKYYPNSDTYYGCASHGRKGEKRRAPAWCDRILWRGAGLKQKRYDRCESRLSDHRPVRALFEVEVGAPRRNLNSLRSFFLSERFDGGRSAAADLLREDGTASSARFGDTI* |
| Osa03g42810  (Os5PTase11) | MGNCASFTPKWGLSDLHCKGMVPIDEDETHEGIKTIRIQKACEFTTSSVLCVCIITWNMNGKMSVEDVTKLVSSNRKFDLLVFGLQEVPKCDVAQVLQETMAETHILLCQKTMQSLQMFLFGAKSSEKYIRELKVDKHAVGGCGGIIGRKKGAVAMYINFSGIRMVFVSCHLAAHENKVEKRNSECQHISHSLFSKNDIQYTKSADITVWLGDLNYRLQGISSIPARKLIEENRQSKLRGKDQLLQEAEKGEVFNGYCEGTLLFKPTYKYNIGSSNYDTSYKIRVPSWTDRILFKVDHTSGLDAVLNSYEALDCIRSSDHKPVRAHLCLKVHGDSA* |
| Osa03g46090  (Os5PTase6) | MRDENSIKTKKLSWSKTFVRKWFNIKTKAKDFHSDYAVEEETAIACLRPTERILFWRGGAKNLFLIISVGVQWRTSFSERDVCKSKKSRTERLPRKSVDRDSRVGNGFDRAYITNTQDYRVFVATWNVGGRSPSSHLNLEDWLHTSPAADIYVIGLQEIVPLNAGNVLLTEDNGPAKKWVALVRKTLNNIDQGSVVYNYHTPSPVPDPIVELNVDFERSSRRPRNSSFFHRRSFQSFNRSSRIDMMDPHSLVDRRFSVCDRISFGSRPSDVDTSMRYGGSSDDENIDEESPSGIYISPMPYGYGAPLCYDDNKRQLINTSRYCLVASKQMVGVFLMVWVRSDIREHVKNLKVSCVGRGLMGYLGNKGSISISMSLHQTSFCFVCTHLTSGQKDGDELRRNADVVEILRKTRFPHVHGVGDEKSPETILDHDRIIWLGDLNYRIALSYRSVKALVEMHNWKQLLEKDQLRIEQRYGRVFSGWKEGRIYFPPTYKYSYNSDRYAGDDMRPNEKRRTPAWCDRILWYGRGLNQLCYVRGESRFSDHRPVYSIFTAEVQIPSQTQFCSFARSTSLMGVDELPYPTYPRSYTDINFY* |
| Osa03g57950  (Os5PTase4b) | MREESNKKSKLSWSKSLVRKWFNIKSKANDFHADYDASQGRNGHGGEWRTSCSEREAGTAKKSRTDRMPKKNADCIRRGRTESDVSRLTEVQDYRIFASTWNVGGKSPSKGLDLDEWLHSSPPADIYILGFQEIVPLNAGNVLGTEDNVPAKKWVSLIRRTLNRNPGASSYGGYHTPSPVPDPVVELDADFEGSSRRHDNLSFFHRRSFQNLSQSLRVEGNYMSSQPRLDRRFSVCDPVSLGGRPSDFDGNFPCAGSPDDEYIEEDGSNGTYFSPFPYGYGTSIAMEENDEQPNTSRYCLVASKQMVGIFLTVWVRSELRNDVKNLKVSCVGRGLMGYLGNKGSISISMSLHHTSFCFICCHLTSGEKEGDELRRNSDVMEILRKTRFPRVRGAGDIKSPETILEHDRIIWLGDLNYRISLSYCSAKALVEMHNWKQLLEKDQLRIERRCGRVFQGWKEGRIYFPPTYKYSFNSDRYSGECVHSKEKRRTPAWCDRILWHGNGLIQLSYVRGESRFSDHRPVYSIFMAEVEIIRQRRRNMGCFNSRVEVEELLPYSYSFGDIKFN* |
| Osa02g27620  (OsFAR3) | MAVGGSYIFTMAGHGGVRGWNLSSPGPIDNIMRSTLIEAEPLYKQFEYMKVLVGSWNVGQEKASYESLRAWLKLPTPEVGLVVVGLQEVDMGAGFLAMSAAKETVGLEGSPNGDWWLDAIGQQLKGYSFERVGSRQMAGLLICVWVRTHLKQFIGDIDNAAVACGLGRAIGNKGAVGLRMRIHDRSICFVNCHFAAHMEAVSRRNEDFDHVFRTMTFATPSSGIMTTSVSSSTGQLLRGANGSRMPELSDTDMIVFLGDFNYRLYDISYDDAMGLVSRRCFDWLKNNDQLRAEMRSGRVFQGLREGDFKFPPTYKFEKHTAGLSGYDSSEKRRIPAWCDRILYRDSRVSSGNECSLDCPVVSSISLYDSCMEATDSDHKPIKSVFNLDIAYVDKQTMRQKYVELMSSNNKVVHLLQELEAFPGVNINNSNIILQDRNPSVVKLQNRTEVIACFEIIGQAPNLSSTHFSAFPAWLKVSPAVGIISPGQTVEVTLQHRDLHSQQNYNGTSLDILPGGATQQKAATVFAKITGVYSTVAKYYEIHVQHQNCRSTLPSRGYNLGDRFF* |
| Osa02g51600  (Os5PTase5a) | MSNHNSPCDIPKPASVDEFVKNGKKKKSFMSSIFRKKGRSGTGSSDKKLLSRRDIVFGLDEKCDDRSELLDSSPAVRKSFSDRHCATKIESLTLSCLDSPHRQFDTREYRVFVGTWNVAGKPPNSSLNLEDFLQIEGLPDIYVLGFQEIVPLNAGNVLVIEDNEPAAKWLGLIYQALNKPQDQSSGDELSPPETSDSRQGGGSGSRDSIPKSSSGGMLFFQKPSLKMLSKNYRVDSALVKTCTCLTDPSTMQRRAREMREFLYRIEASPPPSLASAAAAADEDGGPDAGGELARSSVNYCLIASKQMVGIFLSVWVRRELVQYIGHLRVDSVGRGIMGRLGNKGCIAMSMTLHQTSVCFVCSHLASGEKEGDEVRRNSDVAEIIKSTQFPRICKVPGQRIPDKILDHDRVIWLGDLNYRVALSYDETKTLMGENDWDTLLEKDQLMIERQAGRVFKGWKEGKIYFAPTYKYKQNSDSYAGETAKSKKKRRTPAWCDRILWHGQGIEQLQYIRGESRFSDHRPVCSVFVIEADVDNGSMIRKGYSTLDSRIHFESPIPQRHSFYDDF* |
| Osa09g39970  (Os5PTase2) | MPATATCPKALHAHLLRSGALFADPSAAGPLAAAASLASLPYALSILRAHPTTFSYNSAIRALARGPRPHLAISLYRSMLSHSRSHPNNYTYPPLLAACARLADSDSSSAAAAAAAGVALHASLFRRGLESPDRFIRASLLSLYAAAGDLPAARQVFDLSPPNHRDLPLWNSLLHAYLSRAHYVQVLRLFRTMRTADHVTLLALLSACAHLGALHTARWAHAYLATTCSFPITTNLATALLNMYMRCGDVQTACSLFHSTPTRHKDVHTWTVMIAGLALNGFSTDALHLFTHMKDHNIQPDSVTLTAVLSACTHAGMVDEGKRILRRMPLDYHLQPTIEHYGCTVDLLGRAGLLEEALALIRAVPFKADVALWGALLVACRCHRNFEMGQMVAMEILRLDPQHAGAWVFLSNVYAAAGKWDLVQEVRSSMKQHRIHKPPGSSVVELDGVVYEFLSGDHSHPQSDQIYAMLDEIGKTLSLKGHKPATKLVTFDIDEEDKEVCISQHSEKLAVAFGLINTRRGAVIRIVKNLRICEDCHSVMKECICLRLTVFYPTLWKTVIASHSQECENIIVLRKMLELRTGDIFGAVFIACLIALQIMGSQRGKQSEKSFWPLIVMKKWLNIKPKLNDFSEDEFDTDGGDEDFSDCAEDASDNFFEIHENNHTINRSSGDKIMPLRRLQRRKSESLRVNYISNKDMRFGLYEFIFNPQCIPCHASGDFIIVVTTRVMIGTWNVAGRAPSEDLDLDQWICSQEPADMYILGFQEVVPLSVGNVLGAEDSRTVPKWEGIIRRALNKSQQPKANCKSYSAPLSPLRVPIPSDDGHDDTKREYDKMTENLSPQQQCRDKQTSISKCSCDWLDGTSSLDWPECPLDIPAKISVSNRGLRRVMSMGLFNTDYLENAQGFDLHGVALQDGIRRSYRSSGNLGMSWSEQQEKVDVLSSVDYMSDWTSDDTTSVVGPDERATFAKGESLKPPGNYVRVVSKQMVGIYVSVWVSRKLRQHVNNLEVASVGVGLLGYMGNKVILPSVDCRSLHQIFWFGDLNYRIDMPDAEIRDLVSMKRWDDLLKSDQLTKELTNGNTFAGWKEGLINFPPTYKYETNSSVIAYYGWERVSSNYHIGAQIYHARQMYIQRDLNI* |
| Osa09g32440  (Os5PTase12b) | MPAPPALCVAVDRANRLLWTGHKDGRIRSWRMDLDAAATAPAPPPGGAGDGGGSVGGSNHGGPSNAPVFKEALTWQAYGRTPVLSMVVTSYGEIWSGSEGGVIKAWPYDAIAKSLSLSPEERHMAALLVERAYIDLRNHCTVGNVCSLPASDVKHMLADYSRAKVWTVTSMTFAIWDARTRELLKVFGMDGQVESARLETPVMPEQPIEEEVKVKPSKKDKSQGSLNFFQKSRNALIGAADAVRRVATKGTFVEDNRRTGAVAQAMDGTIWSGCTNGSIILWDGNGNRVQEFQHHTSSVQCIKALGERVWAGYASGIVQVMDVEGNLLAGWTGHSCPVIRMAIGGSYIYTLAHHGGIRGWPLTSPGPLDDILRTELTNKELSYTRMEKINIMVGSWNVAQGKASAESLKSWLGSVSSDVGLVVVGLQEVEMGAGFLAISAAKETVGLEGSANGQWWIDNIGKALDEGTSFHRVGSRQLAALLIAAWARKSLKPYVGDVEAAAVPCGFGRAIGNKGGVGLRIRVYDRKMCFVSNHFAAHLEAVSRRNADFDHIYRTMSFNKPHGSTASATSVQLHRGVNVNGNQVDEVRPDLAEADMIVFLGDFNYRLYGITYDEARDMVSQRSFDWLREKDQLRAEMKAGKVFQGMREGLIKFPPTYKFQKHAPGLGGYDSGEKKRIPAWCDRVLYRDSRPISVADCSLECPVVASITSYVACMDVTESDHKPVRCTFSVDIARVDELIRRQEYGEIIETNEKVRSMLEESSFVPDTTVSTSEIILENQENIVFRITNICETSKAAFEITCEGQSSKKEDATKSEILPRASFGFPLWLEVQPAVGLIKPGETAEITIHHEDFYTQEEFVDGIPQNWWCEDTRDKECVLTVNIRGSTSTETKSHAISIRHHCPATSAPPLIISNPLSSSAAPPINALASEGPPSKRSSKKRESNHHKREQQQQDYAQFGSSEVHDLCRMRCP* |
| Osa09g23140  (Os5PTase9c) | MGEKHIILPRLVPSKLSHRQQLCGHRSVSEISGVVDETLGKRPLDGQNDILRYRVFTSTWNVGGMTPSSDLDLEDWMDSTANSYDIYVLGFQEIVPLNARNVLGPRNSCISTKWNSLIGEALNKRRRRGAVLHQEITNSSATERSAQEEHFRCIMNKQMVGIFMSVWVRSNLRPYIHHLNVSCVGSGIMGYLGNKGSVSIRFVLHETSFCFVCCHLASGGKQGDVLLRNFDAADILVRTRFPGGATQELPKKILDHDQVVLLGDLNYRISLEEAETRLLVEDKNWSILLENDQLLIEFSTGRHFDGWQEGLITFSPTYKYHPNSDQYYWCFDGALGKKKRAPAWCDRILWRGKGLKQIQYDTCNYRLSDHRPVRAVFHAECVIRGDADCACGCIALSSSSE* |
| Osa06g11920  (Os5PTase5b) | MSSNMFGKKGWDSNGMDTSGSVCRSSSDINYINQRARLKSASLNCVGSPPRKNNNATQYRMFVATWNVGGRTPNKRLNLQDFLQVEESPDIYVLGFQEIVPLTAGNVLVLEDNEPAARWLALIHQALNMPQEPADGDEPSPLTPPPSSSTTTSESSNGARTRRRDAVSRSASGNLFFHTPSLKMLSNSYRVDSALVKTCNCSPEHSSVRRRAAEVRESVYLADAPAPAGETAAPAADEDDAPTTEAQCEAGCGGGGGMSYCLIASKQMVGLFLSVWVRKELVEHVGHLRVDCVGRGIMGWLGNKGCIAISMTLHHTSLCFVCSHLASGEKEGDELRRNADVAEILKSAHFPRACRPAPAAARRVPERILDHDRMIWLGDLNYRMSLSYDETRTLLEDNDWDALLEKDQLLIEREAGRVFRGWNEGKICFAPTYKYTHNSDAYAGETAKSKKKRRTPAWCDRILWQGDGIEQLQYLRGESRFSDHRPVCGVFAVEVDGGDGDGGGAAGKIMKGYYSLNARIGGDRSQCHQGDVS* |
| Osa05g41000  (Os5PTase7a) | MAFPDDDEKMKGCRPKLFGTKDKKVVKRADYQSCSAVKSGPSSSKSQSSSPFRTLTEVRSIRLSHLLGHSSSTTKTEPFRIFVSTWNVGGNTPTAELNLDDFLPADDNSDIYVLGFQEIVPLNAGNVLVVEDNEPAARWLALINRTLNKPVDSNADIFQHKPSSSLDSTSSLSSSNLDASFSSRTRTASGSSAIFQKSSLKSIRKPYMPTQRKLLKLCNCSVEMTRKSYKDACFGCPQAYANETDSSEDDTDDRSNDPCGYIVDGMNSAASASRDQLKYNLVSCKRMVGIFITVWAKKELVHHIGHVRTSCIGRGIMGYLGNKGCISVSMTVHQTSFCFICSHLASGEKEGDELRRNLDVLEILRLTQFQRICRAGRRIPEKILDHDRVIWLGDLNYRISLSYEDTKKLLTENNWDALFEKDQLNIERKSGRVFKGWSEEKIYFAPTYKYSSNSDSYAGETATSKKKRRTPAWCDRILWHGDGIVQLSYFRGESKFSDHRPVCGTFIVDVEIQESRSKRRSSNTNIRIGAEELLPTSKSKANKNKGNKGSGT* |
| Osa05g02350  (Os5PTase3) | MRKGNARFPKSSSWPRTKTVVKKWLNLKNEEFHSDCINESFAQGRQERRKSCSDKDGSLLTGRDLSGGWLVESSENLRPPARMFVGTWNVGGRAPDQGLDISSWLLDQQPASSPAHIYVLGFQEIVPLNAGNVLGAEDKGPTYKWLDLIRRALNPSSSERSHSFPSNYPYATEASPERPKNDRVSFSDLLAMEDRLSMVSELDDDSEPSTSNPESSSEEETTDVATRYLRSAGQGYRLAASKQMVGIFLCVWVLADLMPCITSLRVSCVGRGIMGYMGNKGSISISLTVQGSTTMCFVCTHLASGEKDGDEVRRNSDVVEILKRTRFTRRRRLSAPAAAVPSPETILEHDKIIWLGDLNYRLTGSGSGDTQELLDKNDWQALLQKDQLRVEQRAGRVFGGWEEGQISFPPTYKYLADSDTYAAAAAFTSSASKKRTPAWCDRILWRGSGMEQVRYARGESRFSDHRPVNSLFSVQLAAGGNNAKSDHLHLLLLRPRAAGPEAAAGIGLRSSRF* |
| Osa05g45900  (Os5PTase1b) | MMLCCGVDCWCGALRLWRRVVLRKWLNVGSGSGDSDFSADECDASDGELDGEDRDNESYGEGTSLDGLGAGTIGRADWLPYVQHKHLFDKTDMAGDEIKSMPYRLRRRKSETLRAQYIDIRELRICVGTWNLAGKFPPSDLDIQDWLDKEEQADIYVLGFQEIVPLNAGNIFGSEDNSPIAVWEHIIRETLNKICPDKPQYKCHSDPPSPSRFNPSDYVMVMKDELLSESDSDNYGELHPLIKQNDDIAIDNDVVHDKTYENFSAASNGRVHKGKDFSRMDSVKTSDQSPNLSYEKDRSKLEETTKLLYHPERLGMIWPEQPLDMMAQCLRASTSLKALATPASLKSTVNFPNDDLSHQVNSDNGVIKSKRPCFLRIGSISVSMSIHQTHFCFVCCHLTSGEKDGDELKRNADVEEILRRTVFNPLPGLSTPKGILGHERIIWFGDLNYRINLSYERAHELISKQDWDGLFENDQLKRELSKGHTFDGWIEGDISFPPTYKYEFDSEKYVSDEPKSGRRTPAWCDRILSRGKGIRLISYRRGELKLSDHRPVTAVFMADVEVLCHRKLQKALTFTDAEVEYHLVTEEDRT* |
| Gma10G171700 | MDPSSPPPPLLRQNDVASFDRETSSRIYLHSSSSDDDVSPSNSIQSTNRRLDYMLQFLDRKLSADHGHRRHSSGSRAAPLPEFVAKGGGAGIFRLPARGAVHPARPPSLELRPHPLRETQIGRFLRNIVSSQSQLWAASECGVRFWNFKDLYASWCGVGGEEVVARSGDEESAPFRESVWTSPALCLVADEGNRLVWSGHKDGKIRCWKMDDDDDNNDNCDWSNRFTESLSWHAHRGPVLSLTFTSYGDLWSGSEGGGIKIWPWEAVEKSIHLTKEERHSAVIFVERSYVDLRSQLSTNGFSNMLTSDVKYLVSDNLRAKVWSAGYFSFALWDARTRELLKVFNSEGQIENRLDVSSIQDFSVELVSSSRKDKTQSSIGFFQRSRNAIMGAADAVRRVAAKGGFGDDHRRIEALVVTIDGMIWTGCTSGLLVQWDGNGNRIQDFLYHSSAIQCFCTFGMQIWVGYVSGTVQVLDLKGNLIGGWVAHGSPIVKMTVGAGYVFALANHGGIRGWNITSPGPLDSILRSELGGKEFLYTKIENIKILSGTWNVGQGKASLDSLTSWLGSVVSDVSLVVVGLQEVEMGAGFLAMSAAKETVGLEGSSVGQWWLDMIGKTLDEGSTFERIGSRQLAGLVIAVWVKTNIRFHVGDVEVAAVPCGFGRAIGNKGAVGLRIRVYDRIMCFVNCHFAAHLDAVGRRNADFDHVYRTMSFSRPTNLLNTTAAGTSSSVPTFRGTNSAEGMPELSEADMVVFLGDFNYRLDDISYDEARDFVSQRCFDWLRERDQLRAEMEAGNVFQGMREAVITFPPTYKFERHQAGLAGYDSGEKKRIPAWCDRILYRDSCTSLVSECSLECPIVSSVLQYEACMDVTDSDHKPVRCIFSTDIARVDEPIRRQEFGEILESNEKIKYLLKELCKIPETIISTNNIILQNQDTLILRITNKCAEGNALFEIICEGQSTVTGDQKATNHQLRGSFGFPRWLEVSPATGIIRPDQIVEVSVHHEEFQTLEEFVDGVVQNSWCEDSRDKEAILVVKVHGNYTIQPRNHRVRVHHCYSSQKKSLIDSQPDGSRHIQGTVLHRSDFQPFSSSYDVVDQLQKLHSP* |
| Gma10G045100 | MRTELKKKISKSSWPKFNVRKWLNIRSNDDNFHSDYSLPEGWLMDSTNELKHSASVMEAPPVNDTDTLNLRMFVGTWNVGGKSPNEGLNLRNWLMLPSPADIYVIGYIVLSFQEIIPLNAGNVLGPEDSGPASTWLNLIHQALNSNTSSSSGENSPTCSPSEHEQQLYYCLAASKQMVGIFLCLWVRADLYKHVSNLKVSCVGRGIMGYLGNKGSISISMTLYHTTFCFVCTHLASGEKDGDEVRRNLDVSEILKKTKFSQSFKALGQSLPPESILEHDKIIWLGDLNYRLTAGYDDTLELLKKNDWKALLEKDQLRIEQRAGRVFKEWKEGKIYFAPTYKYLFGSDQYVAQTNKSKEKRRTPAWCDRILWKGEGVEQLWYVRGESKFSDHRPVYSLFSVNVDFTSNKLSPSIATSNSSSIISRSCSSRPLTNAALSSSCFAKVQAEEQLLLLTRAHRHRVLT* |
| Gma10G076200 | MFSAVTCICISISHDYSRASDYSRPSDYSRWGSSDDDNGLGDSPSTVSPLSYGGPASTEDGYGMPGRSRYCLLASKQMVGIFLTIWVRSELKDHVRNMKVSCVGRGLMGYLGNKGSISISMSLHETSFCFICSHLTSGQKEGDELRRNSDVMEILKKTRFLRVHDADNEKSPETILEHDRIIWLGDLNYRIALSYRFAKALVEMQNWRALLENDHLRIEQKRGRAFVGWNEGKVYFPPTYKYSTNSDRYAGDDMHPKEKRRTPAW* |
| Gma10G221000 | MKQGSANNQQLFWARVVMRKWLNMASNEPDYTADPDDDNEDDPESDSDNEELGKRTRFGDSREEQAPIESNEFLPRLRRQKSLTSRSQYINKKELRVCVGTWNVGGKLPSDDLDIDDWLGINEPADIYVLGLQEIVPLNPGNIFGAEDTRPVPKWENIIRDTLNRVRPKAPKMKSFSDPPSPSKFKPSDDAPDIEEEILLESDGDIGEEVHPLDEEHNVYDGGADKPTTYEEASNTNFQASDAADIANTEEPIGNDLKRQFSDGKRLSRLNCFRDENLPKKTETSSSQQASKLSRMISSSDRIGLSWPEPPLHLLSQGPLDRPTSFKSVRSFSASKSFRTCQTFKKTIDDIGLLAEIDLEALMKRKRRSSYVRIVSKQMVGIFITIWVRRSLRKHIQNLKVSTVGVGVMGYIGNKGSISISMSIYQTLFCFICTHLTAGEKEGDEHKRNADVREIHQRTHFYSLADIGVPRNILDHERIIWLGDLNYRINLSYEKTRDFISKKQWSKLIEKDQLSKELEKGVFGGWSEGKLNFPPTYKYENNSDKYYGEDPKVGRRTPSWCDRILSYGMGMRLLRYGRTELRFSDHRPVTATYMAEVEVFSPRKLQKALTFTDAEIENEEVMATLGTLYEF* |
| Gma10G246000 | MRDENSKRSKLSWSKKMVRKFFNIKSKTEDSQANGVAYGGGDMEYRGRNSFSEREPCTIKKSKTEKFSRSTDQVRRAKMNLDHPRIIDVQNYSIFVATWNVAGRSPPSTLNLDDWLHSSSPADIYVLGFQEIVPLNAGNILGAEDNGPAKKWLALIRKALNNLPGTSGSSGCYTPSPIPQPVVELNADFEGSARQKNSSFFHRRSFQTTSSGWGMDNDPSVVQPRLDRRYSVCDRVIFGHRPSDFDPSFRWGYRPSDYSRASDYSRPSDYSRWGSSDDDNGLGDSPSTVLFSPMSCGGGGGAGPAFNEDGYAMPGHSRYCLVASKQMVGIYLTIWVRSELKDHVQNMKVSCVGRGLMGYLGNKGSISISMSVHETSFCFICSHLTSGQKEGDELRRNSDVMEILKKTRFPRVQGVDNEKSPQTILEHDRIIWLGDLNYRIALSYRSAKALVEMQNWRALLENDQLRIEQKRGRAFVGWNEGKIYFPPTYKYSTNSDRYAGDDMHPKEKRRTPAWCDRILWYGEGLHQLSYVRGESKFSDHRPVYGIFWAEVESAHGRLKKTMSCSRSRIEVEELLPYSGGYTELSFF* |
| Gma10G028400 | MRDDNSKKSKLSWPKTLVKKWFNIKSKNEDFQADDVLYAGVNQEWRNCSQREEDTIKRSKTERAKRRHSDRMRRGRIDRDAAPVTDVHNYRIFAATWNVAGKSPPSYLSLEDWLHSSPPADIYVLGFQEIVPLNAGNVLGTEDNGPARKWLSLIRKTLNSLPGTSGECHTTSPLPDPIVELDADFEGSMRQKTTSFFHRRSFQSLSHSMRMDNDMSLPQACLDRRLSVCDRMMFGHRPSDYDPCYRWASSDDENGPGDSPVVTHYSPMTYRGCFSMEDRFRQTGQSRYCLVASKQMVGIFLTVWVKSDIRDDVHNMKVSCVGRGLMGYLGNKGSISISMSLHQTSFCFICSHLTSGQKEGDELRRNSDVMEILRKTRFPRVQDMGDESSPQTILDHDRIIWLGDLNYRIALSYRAAKALVEMHNWKVLLENDQLHIERRQGRVFEGWNEGKIYFPPTYKYSNNSDRYAGDERQSKQKRRTPAWCDRILWYGRGLRQLSYVRGESRFSDHRPVYSMFLAEVESVSRNRIKKCSSCSSSRIEVEELLPHSHGYSYTDLSFF* |
| Gma17G153000 | MKTRRGKRSEAFWPSLVMKKWLNIKPKVYDFSEDEVDTETESEDDACSLKDSRLGVREDNRPLRTQSIFPSQISDTPCKGYNTKHRRGKSETLRVQYINTKELRVTIGTWNVAGRAPSKDLDIEDWLCTNEPADIYIIGFQEVVPLSAGNVLGAEDNTPIRKWEAIIRRTLNKSSEPESKHKSYSAPHSPVQKTSSSASVNALADSVDVNPLDMMNEEYLGTFDNDDLEQEEVKSSIFGIGKNLQLRKIHDIDLQTILDWPERPLDATPHTDSSPKLRRVLSSSERTGFSWTDTASKYSNAMKRSHHSSGNLGLLWKEQKVMPEEVIDIIDDLSDMLSDEEDDDDYFEVANDKEVNGISKVKSHRKYVRIVSKQMVGIYVSVWVQRRLRRHINNLKVSPVGVGLMGYMGNKGSVSVSMSLFQSRLCFVCSHLTSGQKDGAEIRRNADVHEILRRTCFSSVFDTDQPQTIPSHDQIFWFGDLNYRINMMDGEVRKLVALKNWDELMNYDQLSNELRSGHVFDGWKEGLINFPPTYKYDFNSDKYIGENPKEGEKKRSPAWCDRILWLGKGIKQLQYRRSENKLSDHRPVNSIFAVDVEVFDHRKLQRALNFTNAAVHPEVFLKEDGDWSY* |
| Gma17G247600 | MGNQLSKRRKSRSKTRPMGFIHNQAHIGIRTVGVEKACNFSTNSDLCIPSYLEYEWPELRNSHFNFAMFIYIYFDLLAVGLQEAPPCPRNKVATLLSAALDESHTLIGKVIMQSLQVNLFGPKDAGPFINGEINLHQSFSYRQIIGRKKGTVAIRINYKGFRMVFISCHLSGTNYVPHARKVEERNSQCKHISHFFFSKFWNPYFRPSHITIWLGDLNYRLQGIDTYPARSLIEQDLHPVSQLLQEAGRGQIFNGFCEGTLTFKPTYKYNKGSSNYDTSHKVMPAPTDRILFRTEDENNMEATLHSYESMDEIYGSDHKPVNKYS* |
| Gma17G000600 | MDDRIDEDEKEKEKEESLAGLTSLPPHRKAHSYSQQLRGTSTHKRHHHVRKHSLDDSRISSSIEASFYDPSDDDDIFSRSSSTNNPGAEEEYNEGADSTTQYQPLQEFIGSGGGTGVFKPPFRASVHPGRPPFLELRPHPLRETQVGKFLRNIACTETQLWAGQESGVRVWEIQNAYEPGNGLGGKVRRGDEDAAPFFESLDTSPTLCLAVDNGNRLVWSGHKDGKIRSWKMDQRFATPFKEGLSWQAHRGPVLAIVFSSYGDLWSGSEGGIIKIWPWESVAKSLSLSPEERHMAALLVERSFIDLRAQVTVNGVCSISSQEVKSLLCDHVRGRVWCAGPLSFSLWDAHTKELLKVFNIEGQVENRVDMSSVQQQDQAVEDEMKVKFVSTSKKEKSQGTSFLQRSRNAIMGAADAVRRVATKGAGAFVEDTKRTEALVQTGDGMIWSGCSNGLLVQWDGTGTRVQDFNRHPCAVQCFCTFGTRLYVGYVSGIIQVLDLEGNLVAAWVAHNGPVIKLAVGCDYVFSLATHGGLRGWIIASPGPVDNIIRSELATKEFIYTRLHNVRILIGTWNVGQGRASQGSLSSWLGSIASDVGIIVVGLQEVEMGAGFLAMSAAKETVGLEGSAMGQWWLDTIGKALQEGKAFERMGSRQLAGLLVSLWVRKNLRTHVGDIDAGAVPCGFGRAIGNKGGVGLRIRVYDRIMCFVNCHLAAHLEAVNRRNADFDHIYRNMVFTRSSNLLNTAAGMVPYLFLLCSLAFSTYLFWLLYSSGLPLVLSVTAGVSTSVHVLRGTNVMGVISEEPKPDLSEADMVVFFGDFNYRLFGISYDEARDFVSQRCFDWLREKDQLRAEMKAGKVFQGMREALIKFPPTYKFERHQPGLGGYDSGEKKRIPAWCDRIIYRDTRSAPVSECNLDCPVVSSILQYDACMDVTDSDHKPVRCKFNVKISHVDRSVRRKEFGVVMTSSEKIRSILEDLCYVPEATVSPNSLVLQNLDTSMLLITNRSTKDKAIYKITCEGQSIVKNDGQAPDYSPRGGFGFPRWLEVTPAAGIIKPEQSVEVSVRHEDLHPSEESANGIPQNWWNEDTRDKEVILVVHVQGSSSVQTSCQQIHVRHCISAKTVQIDSKSNGARRNQIS* |
| Gma09G285200 | MTGKLAEFMWPALVANKILNKRLGSSNFVADYPSNTEPLLGHDQSSLSSKTILNDHKDTQKYKIFVSTWNVGGIFPDEGLNMEDLLETCNNSCDIYLLGFQEIVPLKASNVLGYENNKISTKWNSIIRKALNKSTHHSFKDQLGDDKREDVKKNICCNNKEGENNNHPGQLCESPQDFECIISKQMVGILISVWAKRDLRPFIQHPSVSCVGCGIMGCLGNKGSVSVRFVLHETSFCFVCAHLASGGRGGDEKLRNSNVAEIFSRTSFPRGPMLDLPRKILDHEHVILLGDLNYRISLPEETTRLLVENEDWDYLLEYDQLTMELMRGNMLKEWHEGAITFAPTYKYCPNSDMYYGCCYQGKKAGKKRAPAWCDRIIWFGDGLKQMQYARCESRLSDHRPVNALFIAQVRVSATLKSFQSLFLSERFEQIKTHFGFPHNDEFVCKKQLSFRL* |
| Gma07G273800 | MDDRIDEDEKDKEEEESLAGLTSLPPHRKAHSYSQQLRGTSTHKRHHQVRKHSLDDSRISSNIVEAASFYDCGEDDDILSRSSSTNNPAADEVYSEGADSTTTQYQPLQEFIGSGGGTGVFKPPFRASVHPGRPPCLELRPHPLRETQVGKFLRNIACTKTQLWAGQEGGVRVWEIKNAYDPGKGLGGTVRRGDEDAAPFCESSDTSPTLCLVVDHGNRLVWSGHKDGKIRSWRMDQRFATPFKEGLSWQAHRGPVLSIVLSSYGDLWSGSEGGIIKIWPWESVEKSLSLSPEERHMAALLVERSFIDLRAQVTVNGVCSISSQEVKCLLCDHVRGRVWCAGPLSFSLWDARTKELLKVFNIDGQVENRVDISSVQQQDQAVEDEMKVKFVSTSKKEKSQGTSFLQRSRNAIMGAADAVRRVATKGAGAFVEDTKRTEALVQTGDGMIWSGCTNGLLVQWDGTGTRVQDFNRHPCAVQCFCTFGTRLYVGYVSGIIQVLDLEGNLIAAWVAHNGPVIKLAVGCDYVFSLATHGGLRGWIIASPGPVDNMIRSELAAKELIYTRLHNVRILIGTWNVGQGRASQDSLSSWLGSIASDVGIVVVGLQEVEMGAGFLAMSAAKETVGLEGSAMGQWWLDTIGRALEEGKAFERMGSRQLAGLLVSLWVRKNLRTHVGDIDAGAVPCGFGRAIGNKGGVGLRIRVYDRIICFVNCHLAAHLEAVNRRNADFDHIYRNMVFTRTSSLLNTAAAGVSTAVHVLRGANATGVSSEEPKADLSEADMVVFFGDFNYRLFGISYDEARDFVSQRCFDWLREKDQLREEMKAGKVFQGMREALIKFPPTYKFERHKPGLGGYDSGEKKRIPAWCDRIIYRDTRSAPVSECNLDCPVVSSILQYDACMDVTDSDHKPVRCKFNVKISHVDRSIRRKEFGVVMTSNEKIRSILEDLCDVPEATVSPNSLVLQNLDTSLLLITNRSTKDKAIYKITCEGQSIVKNDGQAPDYSPRGGFGFPRWLEVTPAAGIIKPEQSVEVSVRLEDLHTSEESANGIPQNWWSEDTRDKEVILVVHVQGSSSVQTSCQQIHVRHCMSAKTVRIDSKSNSARRNQIS* |
| Gma07G107000 | MQGFNLKENKIMRKIFSSDNFKGENQNSSEAKKESPSLNQASARCFYHQTKKIFVGSWNIGGITPPKNLDMEDWLDTQNNSADIYVLGFQEIVPLNAANVLGPQNRKVSMKWNSLIGAALNNRTPTKVVEENKTAEPQKIYPLKEHIYAEGEHGQDFQCIISRQMVGMFITIWVRCDLYQTIRHLSILSVGCGIMGCLGNKGSISIRFYLHETSFCFICSHLASGGKEVDRRHRNVNAAHILSRTIFPSGPLHDMPQKIIDHDRVVWLGDLNYRIYMPDSTTKSLIKRGEWETLLKHDQLKMELTEGHVFQGWHEGAIEFPPTYKYRLNSVDYLGCDQQHVSRKRRSPAWCDRIIWFGKGMKQIQYNRSESKLSDHRPVRAMFTADIRVAGTCK* |
| Gma15G223200 | MEIEFEDRQEETVSDMIPSNQQRKKQSFLWKVLAMRERNGRTMERGSNASLDPLSDSSFDNRGSEPSMSSSEAIQNFRVFAATWNVGGQCPTGNLDLNDFLQVRNEPDMYVLGFQEIVPLNAGNVLVLEDNEPAAKWLALINQSLNGSSDLASKGLKLTASFGGPLFSQKPSLKKIKKTFKKLNGKRLKSCNCVLEMERKAAKDFCFRCQESNFNSDDSSTEEEDENFTIPVALATSQMKYSLVACKQMVGIFVSVWMRRELVQYVGHLRICCTSRGIMGCLGNKGCISVSMSFYQTSFCFICSHLASGEKEGDELRRNLDVIEILKNTQFPRICKTPHSRMPDKILDHDRIIWFGDLNYRISLSHDDAKRLVEKRDWPALFNKDQLKMEREAGRVFKGWKEGKIYFAPTYKYAFNSDTYYVEGVKVSKNKRRTPAWCDRILWHGRGIQQLLYVRREFKFSDHRPVCATFNVEVEVMFRGQKKKVSTCNFQNIDDLVSTRSPYFS* |
| Gma13G067500 | MEVENQKQRRYQKLRNWFNSKQKEDRPSSFSLNEIQDGAEDESDDYEGNLSLRSLELDPCISTNKLRVFVGTWNVAGRSPVGSLAVDLDEWLNLKNAADVYVLGFQEIVPLKTLTVIGAEDPAVATSWNQLIGKTLNAKFGCPWMTPMLNCSSCDDDDNNYQYVENPNTKGGNNSNNDKYTLVASKKMVGVFISVWMREEVLRKHCVSNVRVCSVACGVMGYLGNKGSVAVSMSIEGTSFCFVAAHLASGEKKGDEGRRNHQVAEIFRRTSFSRTTKDHHHFPLTILGHDRIFWFGDLNYRLYLEDNFARHLIRKQDWKALQEFDQLQKELEEGGVFEGWKEGDIEFAPTYKYSSSTTNRYCGSLPSRSGEKQRTPAWCDRILWYGKGVEQLHYFRSESKFSDHRPVSALFSTQIEIKSSNRGLMGLHNIPPTMLNPKNGMNKGDEDGKSSLLSSLTKNVQGC* |
| Gma13G132700 | MRTELKKKISKSSWPKFNVRKWLNIRSNDDKFHSDASYYSLPEGWLMDSTNELKHSASVMEAPSVIDIDTLNLRMFVGTWNVGGKSPNEGLNLRDWLMLPSQADIYVIGFQEIIPLNAGNVLGPEDSGPASKWLNLIRQALNSNTSSSGENSPTSSFNSRRQCCPNEQHYYCLAASKQMVGIFLCVWVRADLYKHVSKLKVSCVGRGIMGYLGNKGSISISMTLYHTTFCFVCTHLASGEKDGDEVRRNLDVSEILKKTKFSHSFKALGQPLPPESILEHDKIIWLGDLNYRLAAAYDDTLGLLKKNDWQALLEKDQLRIEQRAGRVFKEWKEGKIYFAPTYKYLFDSDQYVAQTNKSKEKRRTPAWCDRILWRGEGVEQLWYVRGESKFSDHRPVYSLFSVDVDLTCNKLSSPSIAASDSSSIISTRSCSSRPLTNAALSSSCFAKVQAEEQLLLLTRVHRHRVLT* |
| Gma13G185500 | MQMDIEFEDRQEETVSDMIPSNRQRKKQSFLWKVLAMRERNGRTIERSSNDSLDPLSVSSFDNRGSEPSMPSNEAIQNFRVFAATWNVGGQCPTGNLDLSDFLQVRNEPDMYVLGFQEIVPLNAGNVLVLEDNEPAAKWLALINQSLNGSSDLASKGLKPTASFGGPLFSQKPSLKKIKKTFKKLNGKRLRSCNCVLEMERKAGKDFCFRCQESNFNSDDSSTEEEDENFPIPVALATSQMKYSLVTCKQMVGIFVSVWMRRELVQYVGHLRICCISRGIMGCLGNKGCISVSMSFYQTSFCFICSHLASGEKEGDELRRNLDVIEILKNTQFPRICKTPHSRMPDKILDHDRIIWFGDLNYRISLSHDDAKRLVEKRDWPALFNKDQLKMEREAGRVFKGWKEGKIYFAPTYKYAFNSDTYYVEGVKVSKNKRRTPAWCDRILWHGGGIQQLSYVRREFKFSDHRPVCATFNVEVEVMFRGQKKKVSTCNFQNIHDLVSTRSPYYS* |
| Gma02G078700 | MKHHHRSPHHQQRSWAEICCFGWSCIQLFWARVVMRKWLNMGSYESDYSADPVDDDDDSESGSDNEEWGRRSRFANEDEASSESTEFLPKLRRQKSSTYRSQYINKKELRVCVGTWNVGGKLPPDDLDIDDWLGVNEPADIYVLGLQEIVPLNPGNIFGAEDTRPVPKWENIIRETLNRVRPEMPKIKSFSDPPSPSKFKPSDDIPDIEEEILLESDSDIGEEVHPLDEENNICDGTFMGETVNTNLLASDAADIANSGLPVKTDLQRQFSFPKMFDKQHSFSENMVTPFAHQATKLTRMLSGSERMGLSWPEPPLHLLSQRVLDRPTSFKSLKSFKSSKSFKTFNSFKSIMDEMPGIVGLPEIDLEALIKRKRRSPYVRIVSKQMVGIFITVWVRRSLRKQIQNLKVSTVGVGVMGYIGNKGSISVSMSIHQTFFCFICTHLTSGEKEGDELKRNADVHDILRRTHFHSLSYIGLPKKILDHERIIWFGDLNYRINLSNVVTKDLISKKQWSKLVEKDQLIRELKNGVFGGWSEGVLNFPPTYKYEVNSDKYYGEDPKVGKRSPAWCDRILSYGKGMRLLSYKRAELKLSDHRPVTATYMVEVEIFSPRKLQRALTFTDAEIENEQVITNLSNWNLAA* |
| Gma02G145600 | MRDENSKKSKLSWPKTLVMKWFNIKSKNEDFQADDVLYAGVNEEWRNNCSQRDEDAIKRTKTERAKKRHSDRMRRGKIDRDAAQVTDVHNYRIFAATWNVAGKSPPSYLSLEDWLHSSAPADIYVLGFQEIVPLNAGNILGTEDNGPARKWLALIRKTLNSLPGTSGECHTTSPLPDPIVELDADFEGSMRQKTTSFLHRRSFQSLSHSMRMDNEMSLPQACLDRRLSVCDRMMFGHRTSDYDPSYRWASSDDENGPSDSPVVTHYSPMTYRCCFSMEDRDRQTGKSRYCLVASKQMVGIFLTVWVKSDIRDDVLNMKVSCVGRGLMGYLGNKGSISISMSLHQTSFCFICSHLTSGQKEGDELRRNSDVMEILRKTRFPRVQGMGDESSPQTILDHDRIIWLGDLNYRIALSYRAAKALVEMHNWKVLLENDQLHIERRQGRVFEGWNEGKIYFPPTYKYSNNSDRYAGDERQSKQKRRTPAWCDRILWYGRGLRQLSYVRGESRFSDHRPVYSMFLAEVESISRNRIKKCSSCSSSRIEVEELLPHSHGYSYTDLSFF* |
| Gma19G158700 | MRDENSKKSKLSWSKSLVKKWFNIKNKAEDFHSDEVLSQGVDEECSSNYSEREACTIRKSKSERSSRWYSDRMRRGRGKNDLDEAQVTDVYNYRIFAATWNVAGKSPPCYLSLEDWLHTSPPADIYVLGFQEIVPLNAGNVLGTEDNGPARKWLALIRRTLNSLPGTNGGCHTPSPLPNPIVELDADFEGSMRQKATSFFQRRSFQSLSRSMRIDNDMLMPQACLDRRLSVCDRVMFGHRTSDYDPNYRWGSSDEENGTGDSPITAQYSPMSYGGCFSTEDSDRHTGHSRYCLVASKQMVGVFLTVWVKSDIRDDVHNMKVSCVGRGLMGYLGNKGSISISMSLHQTSFCFICSHLTSGQKDGDELRRNSDVMEILRKTRFPPVLDIGGEYSPQTILEHDRIIWLGDLNYRIAISYRAAKALVEMHDWKTLLENDQLCIEQRQGRVFEGWNEGKIYFPPTYKYSNNSDRYAGDDRHSKQKRRTPAWCDRILWYGRGLHQLSYVRGESRFSDHRPVYSMFLAEVESVSCNQIKKSSSSRIEVEELFPHSHGYGYTDLNYF* |
| Gma19G173900 | MRTESKKISKSSWPKLAVRKWLNIKSSAERFHSDYDATTAAIAKERRRSCSDRDRYVVVPDDLSEGWVMDSTSGMKKKSAPGPGTGGPDLRMFVGTWNVGGKSPNEGFNLRNWLTCPSPADIYIIGFQEIVPLNAGNVLGPEDSGPAAKWLGLIREALNSNEELDNTGQNSPKSSPRYCLAASKQMVGIFLSVWVRADLCNHVTNLKVSCVGRGIMGYLGNKGSTSISMTLYNTTFCFVCTHLTSGEKFGDELRRNLDVSEILKKTKFYHSFKSLAHPLPPESILEHDNIIWLGDLNYRLASGYDDTHELLKKNNWQALLEKDQLRIEQKAGRVFKGWNEGNIYFAPTYKYLTNSDHYVAQSSKSKIKRRTPAWCDRILWKGEGLNQMCYVRGESKFSDHRPVYSLFSVQVDMKSKNIAPSTATMPRCCPLKPLTNSSLSSTCCTAKVQAEEQLLLLTRAQSCIDTVPRF* |
| Gma19G016800 | MEVENDQKPRRYHQRLRNWFNSKQKEDRPSSFSLNEIQDGVEDESDDYEGNLSLRSLELDPCISTNKLRVFVGTWNVAGRSPVGSLAVDLDEWLNLKNAADIYVLGFQEIVPLKTLTVIGAEDPAVATSWNQLIGKTLNAKFGCPWMTPMQNSSSCDDDDNNYQYVENPNTKGGNNNNRNNDKYTLVASKKMVGVFISVWMREEVLRKHSVSNVRVCSVACGVMGYLGNKGSVAVSMSIEGTSFCFVAAHLASGEKKGDEGRRNHQVAEIFRRTSFSRTTKDHNHFPLTILGHDRIFWFGDLNYRLYLEDNFARHLIRKQDWKALQEFDQLQKELEEGGVFEGWKEGDIEFAPTYKYSSSTNRYCGSLPSRSGEKQRTPAWCDRILWYGKGVEQLHYFRSESKFSDHRPVSALFSTQIEIKSSNRGLMELHNIPPTMLNPKNGMNRGDEDGKSSLLSLLTKNLQGF* |
| Gma03G173000 | MRTESKKTSKSSWPKLAKWLNITSSAEKFRSDYDAATIAATAKERRKSCSDQDRYVDVPDDLSEGWMMDSTSGMKKKSAPGTGGLELRMFVGTWNVGGKSPNEGLNLRNWLTCPSPADIYVIGFQEIVPLNAGNVLGPEDSGPAAKWLALIREALNTNKCDHEMSHYYTSKKCRQNFSEFLSLDEELDNNGENYPKSLRRYCLAASKQMVGIFLSVWVRADLCNHVTNLKVSSVGRGIMGYLGNKGSTSISMTLYNTTFCFVCTHLASGEKFGDELRRNLDVSEILKKTKFCHSFKSLVHPLSPESILEHDNIIWLGDLNYRLAAGYDDTHELLKKNNWQALLEKDQLRIEQKAGRVFNGWNEGNIYFAPTYKYLTNSDHYVAQSSQSKEKRRTPAWCDRILWKGEGLNQMWYVRGESRFSDHRPVYSLFSVQVDMKSKNIAPSTATLPICCPLKPLTNSSLSSTCCATKVQAEEQLLLLTRAQSCIDTIPRF* |
| Gma03G156400 | MRDENSKKSKLSWPKSLVKKWFNIKSKAEDFHADDVLSQGVDEECSSNYSEREACTIRKSKSERSSRWYSDRMRRGKNDLDEAQVTDVYNYRIFAATWNVAGKSPPSYLNLEDWLHTSPPADIYVLGFQEIVPLNAGNVLGTEDNGPARKWLALIRRTLNSLPGTSGGCHTPSPLPNPIVELDADFEGSMRQKATSFFQRRSFQSLSRSMRIDNDMIKPQACLERRLSVCDRVMFGHRTSDYDPNYRWGSSDEENGTGGSPITTQYSPMSYGGCFTTEDSDRQTGHSRYSLVASKQMVGVFLTVWVKSDIRDDVHNMKVSCVGRGLMGYLGNKGSISISMSLHQTSFCFICSHLTSGQKDGDELRRNSDVMEILRKTRFPPVHDIGDEYSPQTILEHDRIIWLGDLNYRIALSYRAAKALVEMHDWKTLLENDQLCIEQRQGRVFEGWNEGKIYFPPTYKYSNNSDRYAGDDRRSKQKRRTPAWCDRILWYGRGLHQLSYVRGESRFSDHRPVYSMFLAEVESVSCNQIKKSASSRIEVVELFPHSHCYSYTDMNYF* |
| Gma03G081200 | MIHSPLFSFITKSSINHYTMTGKLAQFTWPALVANEILNKRLGSSNFIADFPSNTEPLLSHDQSSLSSKTMLNDQKDTQKNKIFVSTWNVGGIAPDEGLNMEDLLETRNNSYDIYVLGFQEIVPLKASNVLGYQNSKISTKWNSIIREALNKNNTHVLHSFKLDEEGLQGPQDFECIISKQMVGILISVWAKRDLRPFIQHPSVCCVGCGIMGCLGNKGSVSVRFVLHETSFCFVCGHLASGGREGDEKHRNSNVAEIFSRTSFPRRGPMLDLPRKILDHEHVILLGDLNYRISLPEETTRLLVENEDWDSLLEYDQLMMELMRGNMLKGWHEGAIKFAPTYKYCPNSDLYYGCCYHGKKAAKKRAPAWCDRIIWFGNGLKQIQYARCESKLSDHRPVKALFIAQVRVSSAALRSFQNLFLSERFEQIETPFEVSPTYEFVCKKQSSFRL* |
| Gma20G000800 | MTGKLVEFMWPALVANKILNKRLGSSNFIADYPSNNTDIPLLGHDQSSLSSKSILNDHKDTDKYKIFVSTWNVGGIAPDEGLNMEDLLETSNNSCDIYVLGFQEIVPLKASNVLGYENNKISTKWNSIIGKALNKSTHHSFRDDKKEEDVKNNICCNNKEAGNNNNNPGQQCEAPQDFECIISKQMVGILISVWAKRELRPFIQHSSVSRVGCGIMGCLGNKGSVSVRFVLHETSFCFVCCHLASGGREGDEKHRNSNVAEIFSRSSFPRGPMLDLPRKILDHEHVILLGDLNYRISLPEETTRLVVENEDWDSLLEYDQLTMELMRGNMLKGWHEGAIKFAPTYKYCPNSDMYYGCCYQGKNAAKKRAPAWCDRIIWFGNGLKQIQYARCESKLSDHRPVKTLFIAQVRVSSALKCFQSLFLSERFEQIKTHFGLLSNDEFVCKKQLSFRL* |
| Gma20G218600 | MIVRIVVIGTGVRCLISAGILLGLSVRLCFHSLTSHTFPNFDSMDPSSSPLRQNDVASFDRQTSSRIYLHSSSSDDDVSPSHSIQSTNRRLDYMLQFLDRKLSSDHAHRRHSSGSRAAQLPEFVAKGGGAGIFRLPARGAVHPARPPSLELRPHPLRETQIGRFLRNIVSTESQLWAASECGVRFWNFKDLYASWCGVGEEEGVVARNGDEESAPFRESVWTSPTLCLVADEGNRLVWSGHKDGKIRCWKMDDDDDDNNNNNCDWSNRFTESLSWHAHRGPVLSLTFTSYGDLWSGSEGGGIKIWPLEAVEKSIHLTKEERHSAAIFVERSYVDLRSQLSTNGFSNMLTSDVKYLVSDNSRAKVWSAGYFSFALWDARTRELLKVFNSDGQIENRLDVSSIQDFSVELISSSRKDKTQSSIGFFQRSRNAIMGAADAVRRVAAKGGFGDDNRRTEALVVTIDGMIWTGCTSGLLVQWDGNGNRIQDFLYHSSSIQCFCTFGMQIWVGYVSGTVQVLDLKGSLIGGWVAHGSPIVKMTVGAGYVFALANHGGIRGWNITSPGPLDSILRSELGGKEFLYTKIENIKILSGTWNVGQGKASLDSLTSWLGSVASDVSLVVVGLQEVEMGAGFLAMSAAKETVGLEGSSVGQWWLDMIDKTLDEGSTFERIGSRQLAGLVIAVWVKTNIRFHVGDVEVAAVPCGFGRAIGNKGAVGLRIRVYDRIMCFVNCHFAAHLDAVGRRNADFDHVYRTMSFSRPTNLLNTTAAGTSSSVPTFRGTNSAEGMPELSEADMVVFLGDFNYRLDDISYDEARDFVSQRCFDWLRERDQLRAEMEAGNVFQGMREAIITFPPTYKFERHQVGLAGYDSGEKKRIPAWCDRILYRDSCTSLLSDCSLECPIVSSVLQYEACMDVTDSDHKPVRCIFSIDIARVDEPIRRQEFGEILESNEKIKYLLKELCKIPETIISTNNIILQNQDTLILRITNKCAEGNALFEIICEGQSTVTGDQKATNHQLRGSFGFPRWLEVSPATGIIRPDQIVEVSVHHEEFQTLEEFVDGVVQNSWCEDSRDKEAILVVKVHGNYTIQPRNHRVRVHHCYSSKKKPMIDSQPDSSRHIQGTVLHRSDFQPFSSSCDVVDQLQKLHSP* |
| Gma20G148600 | MRDENSKKSKLSWSKKMVRKFFNIKSKTEDSQENGVAYGGGDTEYRGRNSFSEREPCTIKKSKTEKFGRSADQVRRARMNLDHPRIIDVQNYSIFVATWNVAGRSPPSTLNLDDWLHSSPPADIYVLGFQEIVPLNAGNILGAEDNGPAKKWLALIRKTLNNLPGTSGSSGCYTPSPIPQPVVELNADFEGSARQKNSSFFHRRSFQTTSSGWGMDNDPSVVQPRLDRRYSVCDRVIFGHRPSDFDPSFRWGYRPSDYSRASDYSRWGSSDDDNGLGDSPSTVLFSPMSCGGAGPAFNEDGYAIPGHSRYCLVASKQMVGIYLTIWVRSELKDQVQNMKVSCVGRGLMGYLGNKGSISISMSVHETSFCFICSHLTSGQKEGDELRRNSDVMEILKKTRFPRVQGVDNENSPQTILEHDRIIWLGDLNYRIALSYRSAKALVEMQNWRALLENDQLRIEQKRGRAFVGWNEGKIYFPPTYKYSTNSDRYAGDDMHPKEKRRTPAWCDRILWYGEGLHQLSYVRGESKFSDHRPVYGIFCAEVESTHGRLKKTMSCSRSRIEVEELLPYSGGYTELSFF* |
| Gma20G170500 | MKQGSANNQQLLWARVVMRKWLNMASNEPDYTADPDDDNEEDPESDSDNEEWGKRTRFGDSREELAPIESNEFLPRLRRQKSLTSRSQYINKKELRVCVGTWNVGGKLPPDDLDIDDWLGINEPADIYVLGLQEIVPLNPGNIFGAEDTRPVLKWENIIRDTLNRARPKAPKMKSFSDPPSPSKFKPSDDAPDIEQEILLESDGDIGEEVHPLDEEYNVYEGGADKPITDEEALNTNLQASVAADIANTGEPVGNDLQRQFSDGKRLNRLNCFRDENSTEKTDTSSSQQASKLSRMISSSDRIGLSWPEPPLHLLSQRPLDRPTSFKSVRSFSASKSFRTCQTFKQTIDDIGLLAEIDLEALMKRKRRSSYVRIVSKQMVGIFITIWVRRCLRKHIQNLKVSTVGVGVMGYIGNKGSISISMSIYQTLFCFICTHLTAGEKEGDEHKRNADVREIHQRTHFYSLADIGVPRKILDHERIIWLGDLNYRINLSYEKTRDFISKKQWSKLIEKDQLTKELEKGVFDGWSEGKLNFPPTYKYEINSEKYYGEDPKVGRRTPSWCDRILSYGTGMRLLRYGRTELRFSDHRPVTATYMAEVEVFSPRKLQKALTFTDAEIENEEVMATLGPLYEL* |
| Gma05G180400 | MSSFTGARSKANANSEMTKANDTNLCPINTSTITSTSPDTSAKNDKKKKSILPKIFGSKRNGRGSDEETLKSSSAEEGDGVTLDLENKIETRRKAFLEAAPIMRKSFSERETSPGIEGLNLSNFERPMMTPETELQSFRIFVATWNVGGKSPNYDLNLQDFLLVEGSADIYVLGFQEIVPLSAGNVLVIEDNEPAAKWLALISQALNGPRNEYSDSSDSGTGSKTHSSSRELKSPASLNFFQKPSLKVISKSFRAEGSSLLKACNCPVESPSRERRRMRKFSDPMSKLDSELRGDDTVEELLSIAEIPSSPSQSRYSLISTKQMVGIFLTIWTKKELVPHIGHLRADSVGRGIMGCLGNKGCISMSMSLHQTSFCFVCSHLASGEKEGDELKRNSDVAEILKSTQFPRICKNPCRRAPEKIVDHDRIIWLGDLNYRMALSYEETRVLLEDNDWDTLLAKDQLNMERDAGRVFNGFKEGRVVFAPTYKYSHNSDSYAGETVKSKKKRRTPAWCDRILWRGNGIEQLSYIRGESRFSDHRPVCAVFSVDVEVRCRNNRFRKGYSYTSPRPEYEDFIPQRHSFYDY* |
| Gma05G138100 | MWPTLVANKIFKKRLGSSNFIADFPSYKEPLLGIVDIDQNSKTILNDHKDTHKYKVFVSTWNVGGIAPDEDLNIDDLFETFNNSCDIYILGFQEIVPLRASNVLGSENNEISMKWNSKIREALNKKTHQRGKDAKKQELKKNFPNKKENPAKCCEAPHDFQCIISKQMVGLFISVWIRRDLCPFIRHPSVSCVGCGIMGCLGNKGSVSVRFQLHETSFCFVCSHLASGGSEGDEKYRNSNVAEIFSRTSFPRGPLLDLPRTILDHDHVIFLGDLNYRISLPEETTRLLVEKRDWDSLLENDQLIMELMTGNMLRGWNEGAIKFGPTYKYCPNSDIYYGCCYHGKKAEKRRAPAWCDRIVWYGEGLKQLQYTRIESKLSDHRPVKAMFMAEVMVLPELMKNLQSLFLSERYEQIKTPLEVSTTDDFVNRKRSSFRL* |
| Gma05G070400 | MKTRRGKRSEAFWPSLVMKKWLNIKPKVYDFSEDEVDTETESEDDACSLKDSILGVREDNPLRTQSIFPSQTSDAPCKGYKTRHKRGKSETLRVQYINTKEVRVTIGTWNVAGRAPSKDLDIEDWLCTNEPADIYIIGFQEVVPLSAGNVLGAEDNTPIRKWEAIIRRTLNKSSEPESKHKSYSAPHSPVLRTSASADVLADSVDVNSLDMMNEEYLGTFDSDDLEQEEVKSTSGIGKNLQLRKRHDIDLQTILDWPERPLDATPHTDSSPKLRRVLSSSDRTGFSWTDNASKYAGVMKRSHHSSGNLGLLWKEQKVMPEEVIDTIDDLSDVLLDEEDDDYFEVPNDKEVNGIGMVKSHRKYLRIVSKQMVGIYVSAWVQRRLRRHINNLKVSPVGVGLMGYMGNKGSVSVSMSLFQSRLCFVCSHLTSGQKDGAEIRRNSDVHEIIRRTCFSSVFDTDQPQTIPSHDQIFWFGDLNYRINMMDEEVRKLVALKKWDELMNCDQLSNELRSGHVFDGWKEGLINFPPTYKYEFNSDTYIGENQKEGEKRRSPAWCDRILWLGKGIKQLEYRRSENKLSDHRPVSSIFSVDVEVFDHRKLQRALNFTNAAVHHEIFLKEDSDWSY* |
| Gma11G041600 | MKARRGKRSEAFWPSIVMKKWLNIKPKVNDFSEDEVDTETESEDDACSIRGSRMCVREDNPHPLRTEGVQSIFSSLTSDASSPCKGRKTRHRRGKSETLRAQYINTKDVRVTIGSWNVAGRHPCEDLEMDDWLCTEDPADIYIIGFQEVVPLNAGNVLGAEDNTPIPKWEAIIRRCLNKSSEPDSKHKSYSAPPSPVLRTSSAADLLADTIDADNPIPIDMTIEEYVATVDNNEVEQQEVKSIIDIENNLQLRRVFGIDIDWPERSLDAIPQIVDSNSKLRRVLSSSARIGFNRTESSLVYGVGLKRSHRSSGNLGLLWQQQQVIPEVVDSLEDVSDVLSAEGGDTFIVPNDEDEDEFGTTESCPSTRYVRIVSKQMVGIYVSVWVQRRLRRHINNLKVSPVGVGLMGYMGNKGSVSISMSLFQSRMCFVCSHLTSGQKEGAEHRRNSDVHEILRRTCFSSSVFDADQPQTIPSHDQIFWFGDLNYRINMLDAEVRKLVALRKWDELKNYDQLSKELRMGHVFDGWKEGLINFPPTYKYEFNSDRYVGESPKEGEKRRSPAWCDRILWLGKGIKQLQYGRAEIKLSDHRPVSSAFLVEVEVFDHRKLKRALNFTRAAVHPEIFLDEDGEI* |
| Gma11G007800 | MRDENSKKSKLSWSKRMVRKFFNVKSKAEDSYQSNGVAYGGGDVEYRSRNSSFSEREIKKSKTDKFSRNTEQVRRGRVSLDHPRIIDVHNYSIFVASWNVAGRSPPSNLSIDDWLHASPPADIYVLGFQEIVPLNAGNILGAEDNGPAKKWLALIGKTLNNLPGTSGGGGYYTPSPIPQPVVEINADFEGSARQKNSSFFHRRSFQTTSSGWGLDNDSSTMQPRLDRRFSVCDRVIFGHRKSDFDPSYRWGYRPSDYSRASDYSRPSDYSRWGSSDDDNGLGDSPSTVSPLSYGGPASAEDGYGMPGRSRYCLVASKQMVGIFLTIWVRSELKDHVRNMKVSCVGRGLMGYLGNKGSISISMSLHETSFCFICSHLTSGQKEGDELRRNSDVMEILKKTRFPRVHDADNEKSPETILEHDRIIWLGDLNYRIALSYRSAKALVEMQNWRALLENDQLRIEQKRGRAFVGWNEGKIYFPPTYKYSTNSDRYAGDDMHPKEKRRTPAWCDRILWYGEGLHQLSYVRGESRFSDHRPVYGIFWAEVESSHGRLKKSMSCSRNRIEVEELLPYSHGYTELSFF* |
| Gma14G077900 | MVQIITRSIILKSFHLPLSLKSDFSLKIACIYLEIPVFHFANHVTHNMGNQLCKGSKTRSKRRPMGFIHNQASPSHIGIRTVGVEKACSFSIDSDLCISIVTWNMNGQVTFEDFAEMVGSNREFDLLAVGLQEAPPCPRNKVATLLSAALDESHTLIGKVIMQSLQLYLFGLKDAGSFINELKVDKQSVGGCGGIIGRKKGAVAIRINYKGIRMVFISCHLSAHARNVEERNSQCRHISHSLFSKFWNPYSRPSHITIWLGDLNYRLQGIDTYPARSLIEQNLHRRLHGKDQLLQEAGRGQIFNGFCEGTLNFKPTYKYNKGSSNYDTSHKIRVPAWTDRILFRIEDENKMEATLHSYESMDEIYGSDHKPVKAHLCLRLRQIPTN* |
| Gma08G093200 | MWPTLVANKIFKKRLGSSNFIADYPSYKEPLLGIVDIDQNSKTILNDHKDSHKYKVFVSTWNVGGIAPDEDLNIDDLLETCNNSCDIYILGYCQNYFRFQEIVPLKASNVLGSENNEISMKWNSIIREALNKKITHQRDKDAKKQELKNNFPNKKENPAKCCDAPHDFQCIISKQMVGLFISVWIRRDLCPFIRHPSVSCVGCGIMGCLGNKGSISVRFQLHETSFCFVCSHLASGGREGDEKHRNSNVAEIFSRTSFPRGPLLDLPRTILDHDHVILLGDLNYRISLPEETTRLLVEKRDWDSLLANDQLIMELMSGNMLRGWHEGAIKFAPTYKYCPNSDIYYGCCYHGKKAEKRRAPAWCDRIVWCGEGLKQLQYTTIESKLSDHRPVKAMFIAEVRVLPELMKNLQSLFLSERYEQIKTPFEVSTTEDFVNRKRSSFRL* |
| Gma08G138000 | MSSFTGARSKANANSEMTKANEINLCPVNTSTITSTSPDTSAKNEKKKKSILPKIFGSKRNGRGSDEETLKTSSAEEGDGVTLDLENKIETRRKAFLEAAPIMRKSFSERESSPGIEGLNLSNFERPMITMETELQSFRIFVATWNVGGKSPSYDLNLQDFLLVEGSADIYVLGFQEIVPLSAGNVLVIEDNEPAAKWLALISQALNKPRNEYSDSSDSGTGSKTQSSSKESKSPASLNFFQKPSLKVISKNFRAEGSSLLKACNCPVESPSRERRRMRKFSDPMSKLDPELRGDDTVEELLSIAEIPSSASQSRYSLISTKQMVGIFLTIWTKKELVPHIGHLRADSVGRGIMGCLGNKGCISISMSLHQTSFCFVCSHLASGEKEGDELKRNSDVAEILKGTQFPRICKNPCRRAPEKIVDHDRIIWLGDLNYRVALSYEETRVLLEDNDWDTLLAKDQLNMERDAGRVFNGFKEGRVVFAPTYKYSHNSDSYAGETVKSKKKRRTPAWCDRILWRGNGIEQLSYIRGESRFSDHRPVCAVFSVDVEVRSRNNRFRKGYSYTSPRPEYEDFIPQRHSFYDY* |
| Gma01G235300 | MTDGNSKKSKLSWSKRMVRKFFNIKSKAEDSYQSNGVAYGGDDVEYRSRNSSFSEREIKKSKTEKFSRNTEQVRRGRVSLDHPRIIDVHNYSIFVATWNVAGRSPPSNLSIDDWLHASPPADIYVLGFQEIVPLNAGNILGAEDNGPAKKWLALIGKTLNNLPGTSGGGGYYTPSPIPQPVVEINADFEGSARQKNSSFFHRRSFQTTSSGWGMDNDTSTMQPRLDRRFSVCDRVIFGHRKSDFDPSFRWGYRPSDYSRASDYSRPSDYSRWGSSDDDNGLGDSPSTVSPLSYGGPASAEDGYGMPGRSRYCLVASKQMVGIFLTIWVRSELKDHVRNMKVSCVGRGLMGYLGNKGSISISMSLHETSFCFICSHLTSGQKEGDELRRNSDVMEILKKTRFPRVHGADNEKSPETILEHDRIIWLGDLNYRIALSYRSAKALVEMQNWRALLENDQLRIEQKRGRAFVGWNEGKIYFPPTYKYSTNSDRYAGDDMHPKEKRRTPAWCDRILWYGEGLHQLSYVRGESRFSDHRPVYGIFWAEVESSHGRLKKSMSCSRNRIEVEELLPYSHGYTELSFF* |
| Gma01G200500 | MKARRGKRSEAFWPSIVMKKWLNIKPKVNDFSEDEVDTETESEDDACSIRGSRMCVREDNPHPLRTEGVQSIFPSVTSDASPCKGRKTRHRRGKSETLRAQYINTKEMRVTIGTWNVAGRHPCEDLEIDDWLCTEDPADIYIIGFQEVVPLNAGNVLGAEDNTPIPKWEAIIRRSLNKSSEPDSKHKSYSAPPSPVLRTSSAADLLADTIDADNPIPIDMMIEEYVATVDNNEMEQQEVKSIIDIENNLHCESTKQLSQNDLAELRVFVYVDSLEDVSDVLSAEDGDTFIVPNNEDDDEFGTTESCPSPRYVRIVSKQMVGIYVSVWVQRRLRRHINNLKVSPVGVGLMGYMGNKGSVSISMSLFQSRMCFVCSHLTSGQKEGAEHRRNSDVHEILRRTCFSSSVFDADQPQTIPSHDQIFWFGDLNYRINMLDAEVRKLVALKKWDELKNYDQLSKELRMGHVFDGWKEGLINFPPTYKYEINSDRYVGERPKEGEKRRSPAWCDRILWLGKGIKQLQYGRAEIKLSDHRPVSSAFLVEVEVFDHRKLKRALNFTRAAVHPEIFLDEDGEI* |
| Gma16G161500 | MKRHHRSPHHQQRSWAEICCFGWSCLQLFWARVVMRKWFNMGSYESDYSADPVDDDSESGSDNEERGRQSQFADNRCNEDEASSESSEFLPKLRRQKSSTYRSQYINKNELRVCVGTWNVGGKLPPDDLDIDDWLGINEPADIYVLGLQEIVPLNPGNIFGAEDTRPVPKWENIIRETLNRVRPEMPKIKSFSDPPSPSKFKPSDDVPDIEEEILLESDSDIGEEVHPLDEENNICDGTFMGETVNTNLLASDAADIANTTLPVKTDLQRQFSFPKMFDRQKSFSENMDTSFAQQATKLTRMLSGSERVGLSWPEPPLHLLSQRVLDRPTSFKSLRSFKSSKSFKTYNSFKSIMDEMPGIVSLPEIDLEALIKRKRRSSYVRIVSKQMVGIFITIWVRRSLRKQIQNLKVSTVGVGVMGYIGNKGSISVSMSIHQTLFCFICTHLTSGEKEGDELKRNADVYEILRRTHFHSLSYVGLPKNILDHERIIWFGDLNYRINLSNVETKALISKKQWSKLVEKDQLMLELKNGVFGGWSEGVLNFPPTYKYEVNSDKYYGEDPKVGKRSPAWCDRILSYGKGMRLLSYRRAELKLSDHRPVTAKYMVEVETFSPRKLQRALTFTDAEIENEQVMTNLSNWNLAA* |
| Cre07g352400 | MKKLFGQVARKAVALADDIASYNDGRHVPAKARPGGAPKGGDGGAGDGGLDPVLEHLLRNVRSRHVRQHFARTPATEYTRHRNLTIQVATYNVGGKKPHPGIRLDDWLGPGAAGAGAGARVPPQAGPAAAAAAPGGADVVAVGFQELVPLNAGSVMGVLANDNVDAWDRCLAAHLNGEEWAAQRYGTLGPAAPGTAAAAAAAAAMLDAKWQGGPPPRSQLEGGGGGGGAGGGGAEGGSGVEDSTYVQVASKQLVGVYLSVWVRRSLLSAVHGVQVTTVATGFGGYLGNKGAVAARLRLFDSSLVFVAAHLTAGEAEGDELKRNADVADILRRAAFASGLDGGGAVPMTASAAAAVSASLGPGHWPAWPCGITDHDLAIWVGDLNYRLAVAPAAAAAAAQPGSSNSGAGAAAAAPVAPKAAGGGGLLTDADARAAIRSGKLDALINVDQLHRERTAGRVFKGWHEGRITFAPTYKYKVGTNTYNGDDAPSASASSAALAPQPASQSQADLAVAEDADSASVTGPSAVVGPDAESHKRRTPAWCDRVLWWTRAGAHDQAAAAGSSGTAAATLKQLGYWRGELAFSDHRPVSSLFSAQVVSYDRPKIESLLEAALRAVDLMQQSMRPKVTVEPVVLEAGDWVAPGRHVRLRVTLSNTGPVEAIWHFIPPPNAGGGGGKGGGGKFGLDDETPPLPPWLTATPAEGVLAAGATCELDLSVLVAGGPGGAAAALSADVAGSGRGGGGSSAGGSLDCIVILRIEDSGDKFISIGGRYRRSFLGMPLAALLAPGRAGGVLPAAEAEQLAAGLGVGPLALTPAPAPEAGSEGEAAAVPREVAALLAALAADGGAALRTPGILVDSAPEAAARAGAAPESEPAAATAPAAAEAAEADAGVEAAAAAQQPASRAGQCALLRALEAVRLPLDGGCGVPPSAAPHDVAALLLLWCGQLPQPLISTAAADAAAAAPPVSPGDAAALLRRHCDAASRGVLAALLPVLRSALAPAAVAANGLSAARLAGALSAWWLPPLAAGASPDAGANRRRLLRMLLEPAAAGESLV* |
| Cre13g563550 | MTEQASQEQEALLEDAIALLDSLLVEIGATGGEGEPEQQHHRSNATAAHGDPQGDTALALPPAAASAGVAGDRQFVNAAPAACSAVRAGSGYLLHQPYGGAALSSSPSAAPAHVGLSALSSPAHCSQPQAHNPHQPHQLKVLSQLQQGDGHSTAAALQQGVSQQPHTPPHLAAPTRREPLSHALTEPAPGAAPDHHHHHNEQHHNLNHSNSSAAALTSGTGTGGGGAGVPAGGGAAVPSSAAPATAAPFAPTSITASTIANRGPVPSTDARAGGGAQSSTTAKAAARRSAGAGSPPPPPAASAAVARRSSGAAAYGVSAEADEKLARAMERAAQMPDPAKGSRLLDVADGGGGGSGRRVSALLMNGGGGGRGLTDGGGGGGGGAAAFNTALLNDLVDESLANRPGEGAGESGDSSPARSAPSGVLRGQGPGALRRGDTRAGSLLGSGGSGGGGGLLAPGGAGGLVGAGGLGTEQGLKLWAGNKSRVEKARLAVRLLAATGLSRSGAGGGGGGGGGGHHTTARDAAIDPFAVVSCEEVTHTSKALVRTQDPFWDEFFVFDVAQPAFAVLKIKVYDHRHCWRPAFVGQVRIPVHSIAEFPARFAPPSWHTLRSRAGRGIRGQVQMQLFYTAEWVHRPLRVFAGTWNVGNAQPAADLSPWLQGVSSLQHDLVAIGVQECAYKLGGGAGAGGGQAAEEGEEEDDDDGVILTLAEREGGGIMSDEMREEELLLARQLGIPAGAAAAAAAAAAGTAVGSGGPPGEWSGPEDSDTQSAAAAAGPSWRGGSGPQVGAEPSAGSISRRGPAPRVPPVAGAAATPSGPQVRLRAGRGGMYLGVTVPAGVGEEEGGRGGDEGTPLSPLSPLSPLSPGSGEVSREASVTATSPAAAGGAFGRQAAAARALLGAAGAGGGGGGGGRSHAHSSAMMPASAAAAILGRPGHDTPAGVGLLHSPLRGRHPPPPDTAGRMRRSSNLDCSAYAAASAATTPRSPAPSWAPAWAADASFTTVGGGGAATAAAAAAAVAQASWTAGSTGGGGGAYGPQVSGVSMSVHNAYATFGSVAGAAAAGGGGGGGAGGGGGGQAVKSRGGGVLSDVTAKVGVVGGGEFREVWEERIKEAVGPGYFKVAGVHMGQIRLLLFARNDIYAAVSDVRTGKQATGVAGVATNKGGVGVSLRVWDTTLAFINSHLAAHQDKTRARNAHYRDIVRGLKPLASDPGGVMDALTAPHHVVWMGDLNYRLDWGAQAHTPTESPAPGDFADLVREVRRGGFGRLVEADQLRKEVGAKRAFLGFTEGPIAFEPSFKVRRRRGHDYNPQRSPAYCDRVLYRSNLPLKQIRCVSYFSPADVASSDHKPVAAELLVPTVWRTTVDDSSDPAHHSHHHHHHHQHNTAQTQEEAWNPHGPGGGGQGQGQGQGQGQGVGVGVGGHLAGLLPGLHHFGGGGGVGTAAAAADADGDGGGGVRLRLVFRHLRARGLFMLRSRRRSAGAAGTPPSADEFPNPQLLLTAPCIYAGATVRSRVALRTRAPIFADDPAAAHECGDGDGGGGAADPVERSRRGAGGGQLQLQLQPQFSIKASATSRSLSSALTGLGGGAGGAGAGGGEGVAAAAAMHDGGGGRGVGPLVVDLKSAAVTEMAHFRLWVRVVDQRGTAAAAAAAAVAAGGSGAGGSAGASGGAGGGGGGGGFASRVKKKFRGGGGKGKTQSGGAATGPGGGGGGGAAGSASVLARGVLPLADAVTSLLQDAAAWAPFQVQLELHGLPAGTLEGHMRIEVVDRGRAPDKQAADRAPHGAGDNSSAAAAVAGAAGGKALRRSAMGIAGTDAGGGAAPVAATGAPEHGDSAAAAGAAAAPAPPQPHQQAAAATGGLRGPAGPSRLGPAPSGQPLQQRGAGASARTPPGRDSHGNSNAVSAGLFARLRASLRLLQG* |
| Cre12g530050 | MSQDPFAPLTGKVAGAAGFSSTPATGFNPLTAGLHQPAPAPGPGWVSFDDGPSVGAAPAFPAPQPQLAQAPMTSGMPGQVPGILPPAAGGLGTATSFSPLTTAANKSTFPTFAALHASIAQPQTAAATTQPAIHPFSGLPAAAPVVVKPAAVGGSGAAVHANPFAASANPQGATAAARSNDPFASLAMSQPPARPPPMGASSSRRHVTDPFAAAEAPPAFPTFAAIHGQAPPPPQPHSALTPQPASTGSPSPLPVFPAFAAAPHLPPQGAALAAGPPASNVQQPASVDAASFDAFVSSALRPVPAPLAPVGAAAAAVVTPPPPPPAYTAALSMSPAAGTGPAPPPPPTYDDAVGISTLPLPPPPAYTDALAMPEAPSLGVDGSGVLTPPLPPPGYDEARVMPSADSSGALPNDVSLTGLVQGRPLPGARAQGPSGLAMGSLGSAVVSSGAEENWASFGSEVHAAPHLTRQMTHATAAVAEVPLPRPPPTATFGPGGAALMHSGGVHLSHAVPPPPPPMAPPPLPKQVTIEVRLAPLKPKEAALPVRLVAGMGNVFAAPGGEGVSALQWMLWPLQPPAGAGGSGPHGPVVDGALPLAYQMPAGGKQARDDGGSARAGDKQAVEDWDSAPAAMLFVPPSCEAAVTCMLLDEQSGSLWTGHKDGKVARWSVLQGRVAQYQHHWKAHAYGKVTSLVLTPWGELWTASSSGSVRAWQYLAAIPATRPPIRMFECRRERLARGSTLRNAAPRPHSKVRLMALGPGGRVVWTAGRSGMALWGAYDGEFLGSLSPGTERETGAPGGTAGTASAGATGAVYPSGGGLARPGTMSNYAAAYAGETGAGGVLGREGRDAQQQMDINSRTGLEPVLIERKFERAPPPEDNDDGDGGTEADLGTQVFKGLAGAAKFAAKWGKKIAKNVGELQAAASGGGKDSAADDWAEGGKGAGYGAAGGGGGSSAGLAGRGKVVAMVPGLDGSMFVAFKNGVVDKYTEWGKLLWSRDFGREPQLCCAALAGTQLWLGGTDGCIRAVTAAAGELSRAWKAFDFPVVALAHDANPFRGLGLVYGLSEHGSVRAWPAALPDEATLVQWRDGLEPCLTRHQLRVLAGTWNVNETRPAPSSLTTWLRSGAATADIVAIALQEVEVGTSSVAMDAARNLLYKTMLERGNQNAQWWASELAVALTTASGGASWDRVGLRQMSGMLALVFCRAQLRPHVGEVATASVPCGVMGVGGNKGAVAVSMSVYRRRIMFVCSHFAAHQERVDERNDNYNKIVRQLHFENTSKAAARMASHQQQQQQQQQQPGAEPVLIRDDSDLDAAAAAVVQSSAEGGGADAGAAVAQAIAEAAADDGHGPGMADAAMLVWAG  DFNYRINGPYLAVVEAARAGRFAELYTMDQCREQMEKGNVFRGLREPLPLGHPLFVPTYKFDKGEPVRPGRDGRLELPYDTSDKQRVPAWTDRIFYRGSRPGSLDMAGEEVQVGVAQPLDYNCCLELNDSDHKPVYAILQVLLPGYKQEAKRLHSLAIAAVLHRAAVTSAIKSAAAVDASAVVGGPRPPPLPPPVHASTHSLQVRAGIETPSFVDVRNSSTAALLVHVSVQRPGPGAGTPGPLPTWLEVSPTNFILLPADAHAGPGTGEPGQGSLVRVFLRAASGASEGGRVPGPVRLHFSVRPVWAAPQTLAGVPGPVVSVSVIG* |
| HsSYNJ2 | MALSKGLRLLGRLGAEGDCSVLLEARGRDDCLLFEAGTVATLAPEEKEVIKGQYGKLTDAYGCLGELRLKSGGTSLSFLVLVTGCTSVGRIPDAEIYKITATDFYPLQEEAKEEERLIALKKILSSGVFYFSWPNDGSRFDLTVRTQKQGDDSSEWGNSFFWNQLLHVPLRQHQVSCCDWLLKIICGVVTIRTVYASHKQAKACLVSRVSCERTGTRFHTRGVNDDGHVSNFVETEQMIYMDDGVSSFVQIRGSVPLFWEQPGLQVGSHHLRLHRGLEANAPAFDRHMVLLKEQYGQQVVVNLLGSRGGEEVLNRAFKKLLWASCHAGDTPMINFDFHQFAKGGKLEKLETLLRPQLKLHWEDFDVFTKGENVSPRFQKGTLRMNCLDCLDRTNTVQSFIALEVLHLQLKTLGLSSKPIVDRFVESFKAMWSLNGHSLSKVFTGSRALEGKAKVGKLKDGARSMSRTIQSNFFDGVKQEAIKLLLVGDVYGEEVADKGGMLLDSTALLVTPRILKAMTERQSEFTNFKRIRIAMGTWNVNGGKQFRSNVLRTAELTDWLLDSPQLSGATDSQDDSSPADIFAVGFEEMVELSAGNIVNASTTNKKMWGEQLQKAISRSHRYILLTSAQLVGVCLYIFVRPYHVPFIRDVAIDTVKTGMGGKAGNKGAVGIRFQFHSTSFCFICSHLTAGQSQVKERNEDYKEITQKLCFPMGRNVFSHDYVFWCGDFNYRIDLTYEEVFYFVKRQDWKKLLEFDQLQLQKSSGKIFKDFHEGAINFGPTYKYDVGSAAYDTSDKCRTPAWTDRVLWWRKKHPFDKTAGELNLLDSDLDVDTKVRHTWSPGALQYYGRAELQASDHRPVLAIVEVEVQEVDVGARERVFQEVSSFQGPLDATVVVNLQSPTLEEKNEFPEDLRTELMQTLGSYGTIVLVRINQGQMLVTFADSHSALSVLDVDGMKVKGRAVKIRPKTKDWLKGLREEIIRKRDSMAPVSPTANSCLLEENFDFTSLDYESEGDILEDDEDYLVDEFNQPGVSDSELGGDDLSDVPGPTALAPPSKSPALTKKKQHPTYKDDADLVELKRELEAVGEFRHRSPSRSLSVPNRPRPPQPPQRPPPPTGLMVKKSASDASISSGTHGQYSILQTARLLPGAPQQPPKARTGISKPYNVKQIKTTNAQEAEAAIRCLLEARGGASEEALSAVAPRDLEASSEPEPTPGAAKPETPQAPPLLPRRPPPRVPAIKKPTLRRTGKPLSPEEQFEQQTVHFTIGPPETSVEAPPVVTAPRVPPVPKPRTFQPGKAAERPSHRKPASDEAPPGAGASVPPPLEAPPLVPKVPPRRKKSAPAAFHLQVLQSNSQLLQGLTYNSSDSPSGHPPAAGTVFPQGDFLSTSSATSPDSDGTKAMKPEAAPLLGDYQDPFWNLLHHPKLLNNTWLSKSSDPLDSGTRSPKRDPIDPVSAGASAAKAELPPDHEHKTLGHWVTISDQEKRTALQVFDPLAKT |
| HsSYNJ1 | MAFSKGFRIYHKLDPPPFSLIVETRHKEECLMFESGAVAVLSSAEKEAIKGTYSKVLDAYGLLGVLRLNLGDTMLHYLVLVTGCMSVGKIQESEVFRVTSTEFISLRIDSSDEDRISEVRKVLNSGNFYFAWSASGISLDLSLNAHRSMQEQTTDNRFFWNQSLHLHLKHYGVNCDDWLLRLMCGGVEIRTIYAAHKQAKACLISRLSCERAGTRFNVRGTNDDGHVANFVETEQVVYLDDSVSSFIQIRGSVPLFWEQPGLQVGSHRVRMSRGFEANAPAFDRHFRTLKNLYGKQIIVNLLGSKEGEHMLSKAFQSHLKASEHAADIQMVNFDYHQMVKGGKAEKLHSVLKPQVQKFLDYGFFYFNGSEVQRCQSGTVRTNCLDCLDRTNSVQAFLGLEMLAKQLEALGLAEKPQLVTRFQEVFRSMWSVNGDSISKIYAGTGALEGKAKLKDGARSVTRTIQNNFFDSSKQEAIDVLLLGNTLNSDLADKARALLTTGSLRVSEQTLQSASSKVLKSMCENFYKYSKPKKIRVCVGTWNVNGGKQFRSIAFKNQTLTDWLLDAPKLAGIQEFQDKRSKPTDIFAIGFEEMVELNAGNIVSASTTNQKLWAVELQKTISRDNKYVLLASEQLVGVCLFVFIRPQHAPFIRDVAVDTVKTGMGGATGNKGAVAIRMLFHTTSLCFVCSHFAAGQSQVKERNEDFIEIARKLSFPMGRMLFSHDYVFWCGDFNYRIDLPNEEVKELIRQQNWDSLIAGDQLINQKNAGQVFRGFLEGKVTFAPTYKYDLFSDDYDTSEKCRTPAWTDRVLWRRRKWPFDRSAEDLDLLNASFQDESKILYTWTPGTLLHYGRAELKTSDHRPVVALIDIDIFEVEAEERQNIYKEVIAVQGPPDGTVLVSIKSSLPENNFFDDALIDELLQQFASFGEVILIRFVEDKMWVTFLEGSSALNVLSLNGKELLNRTITIALKSPDWIKNLEEEMSLEKISIALPSSTSSTLLGEDAEVAADFDMEGDVDDYSAEVEELLPQHLQPSSSSGLGTSPSSSPRTSPCQSPTISEGPVPSLPIRPSRAPSRTPGPPSAQSSPIDAQPATPLPQKDPAQPLEPKRPPPPRPVAPPTRPAPPQRPPPPSGARSPAPTRKEFGGIGAPPSPGVARREMEAPKSPGTTRKDNIGRSQPSPQAGLAGPGPAGYSTARPTIPPRAGVISAPQSHARASAGRLTPESQSKTSETSKGSTFLPEPLKPQAAFPPQSSLPPPAQRLQEPLVPVAAPMPQSGPQPNLETPPQPPPRSRSSHSLPSEASSQPQVKTNGISDGKRESPLKIDPFEDLSFNLLAVSKAQLSVQTSPVPTPDPKRLIQLPSATQSNVLSSVSCMPTMPPIPARSQSQENMRSSPNPFITGLTRTNPFSDRTAAPGNPFRAKSEESEATSWFSKEEPVTISPFPSLQPLGHNKSRASSSLDGFKDSFDLQGQSTLKISNPKGWVTFEEEEDFGVKGKSKSACSDLLGNQPSSFSGSNLTLNDDWNKGTNVSFCVLPSRRPPPPPVPLLPPGTSPPVDPFTTLASKASPTLDFTER |
| HsINPP5J | MEGQSSRGSRRPGTRAGLGSLPMPQGVAQTGAPSKVDSSFQLPAKKNAALGPSEPRLALAPVGPRAAMSASSEGPRLALASPRPILAPLCTPEGQKTATAHRSSSLAPTSVGQLVMSASAGPKPPPATTGSVLAPTSLGLVMPASAGPRSPPVTLGPNLAPTSRDQKQEPPASVGPKPTLAASGLSLALASEEQPPELPSTPSPVPSPVLSPTQEQALAPASTASGAASVGQTSARKRDAPAPRPLPASEGHLQPPAQTSGPTGSPPCIQTSPDPRLSPSFRARPEALHSSPEDPVLPRPPQTLPLDVGQGPSEPGTHSPGLLSPTFRPGAPSGQTVPPPLPKPPRSPSRSPSHSPNRSPCVPPAPDMALPRLGTQSTGPGRCLSPNLQAQEAPAPVTTSSSTSTLSSSPWSAQPTWKSDPGFRITVVTWNVGTAMPPDDVTSLLHLGGGDDSDGADMIAIGLQEVNSMLNKRLKDALFTDQWSELFMDALGPFNFVLVSSVRMQGVILLLFAKYYHLPFLRDVQTDCTRTGLGGYWGNKGGVSVRLAAFGHMLCFLNCHLPAHMDKAEQRKDNFQTILSLQQFQGPGAQGILDHDLVFWFGDLNFRIESYDLHFVKFAIDSDQLHQLWEKDQLNMAKNTWPILKGFQEGPLNFAPTFKFDVGTNKYDTSAKKRKPAWTDRILWKVKAPGGGPSPSGRKSHRLQVTQHSYRSHMEYTVSDHKPVAAQFLLQFAFRDDMPLVRLEVADEWVRPEQAVVRYRMETVFARSSWDWIGLYRVGFRHCKDYVAYVWAKHEDVDGNTYQVTFSEESLPKGHGDFILGYYSHNHSILIGITEPFQISLPSSELASSSTDSSGTSSEGEDDSTLELLAPKSRSPSPGKSKRHRSRSPGLARFPGLALRPSSRERRGASRSPSPQSRRLSRVAPDRSSNGSSRGSSEEGPSGLPGPWAFPPAVPRSLGLLPALRLETVDPGGGGSWGPDREALAPNSLSPSPQGHRGLEEGGLGP |
| HsINPP5K | MSSRKLSGPKGRRLSIHVVTWNVASAAPPLDLSDLLQLNNRNLNLDIYVIGLQELNSGIISLLSDAAFNDSWSSFLMDVLSPLSFIKVSHVRMQGILLLVFAKYQHLPYIQILSTKSTPTGLFGYWGNKGGVNICLKLYGYYVSIINCHLPPHISNNYQRLEHFDRILEMQNCEGRDIPNILDHDLIIWFGDMNFRIEDFGLHFVRESIKNRCYGGLWEKDQLSIAKKHDPLLREFQEGRLLFPPTYKFDRNSNDYDTSEKKRKPAWTDRILWRLKRQPCAGPDTPIPPASHFSLSLRGYSSHMTYGISDHKPVSGTFDLELKPLVSAPLIVLMPEDLWTVENDMMVSYSSTSDFPSSPWDWIGLYKVGLRDVNDYVSYAWVGDSKVSCSDNLNQVYIDISNIPTTEDEFLLCYYSNSLRSVVGISRPFQIPPGSLREDPLGEAQPQI |
| HsINPP5B | MDQSVAIQETLAEGEYCVIAVQGVLCEGDSRQSRLLGLVRYRLEHGGQEHALFLYTHRRMAITGDDVSLDQIVPVSRDFTLEEVSPDGELYILGSDVTVQLDTAELSLVFQLPFGSQTRMFLHEVARACPGFDSATRDPEFLWLSRYRCAELELEMPTPRGCNSALVTWPGYATIGGGRYPSRKKRWGLEEARPQGAGSVLFWGGAMEKTGFRLMERAHGGGFVWGRSARDGRRDEELEEAGREMSAAAGSRERNTAGGSNFDGLRPNGKGVPMDQSSRGQDKPESLQPRQNKSKSEITDMVRSSTITVSDKAHILSMQKFGLRDTIVKSHLLQKEEDYTYIQNFRFFAGTYNVNGQSPKECLRLWLSNGIQAPDVYCVGFQELDLSKEAFFFHDTPKEEEWFKAVSEGLHPDAKYAKVKLIRLVGIMLLLYVKQEHAAYISEVEAETVGTGIMGRMGNKGGVAIRFQFHNTSICVVNSHLAAHIEEYERRNQDYKDICSRMQFCQPDPSLPPLTISNHDVILWLGDLNYRIEELDVEKVKKLIEEKDFQMLYAYDQLKIQVAAKTVFEGFTEGELTFQPTYKYDTGSDDWDTSEKCRAPAWCDRILWKGKNITQLSYQSHMALKTSDHKPVSSVFDIGVRVVNDELYRKTLEEIVRSLDKMENANIPSVSLSKREFCFQNVKYMQLKVESFTIHNGQVPCHFEFINKPDEESYCKQWLNANPSRGFLLPDSDVEIDLELFVNKMTATKLNSGEDKIEDILVLHLDRGKDYFLSVSGNYLPSCFGSPIHTLCYMREPILDLPLETISELTLMPVWTGDDGSQLDSPMEIPKELWMMVDYLYRNAVQQEDLFQQPGLRSEFEHIRDCLDTGMIDNLSASNHSVAEALLLFLESLPEPVICYSTYHNCLECSGNYTASKQVISTLPIFHKNVFHYLMAFLRELLKNSAKNHLDENILASIFGSLLLRNPAGHQKLDMTEKKKAQEFIHQFLCNPL |
| HsOCRL1 | MEPPLPVGAQPLATVEGMEMKGPLREPCALTLAQRNGQYELIIQLHEKEQHVQDIIPINSHFRCVQEAEETLLIDIASNSGCKIRVQGDWIRERRFEIPDEEHCLKFLSAVLAAQKAQSQLLVPEQKDSSSWYQKLDTKDKPSVFSGLLGFEDNFSSMNLDKKINSQNQPTGIHREPPPPPFSVNKMLPREKEASNKEQPKVTNTMRKLFVPNTQSGQREGLIKHILAKREKEYVNIQTFRFFVGTWNVNGQSPDSGLEPWLNCDPNPPDIYCIGFQELDLSTEAFFYFESVKEQEWSMAVERGLHSKAKYKKVQLVRLVGMMLLIFARKDQCRYIRDIATETVGTGIMGKMGNKGGVAVRFVFHNTTFCIVNSHLAAHVEDFERRNQDYKDICARMSFVVPNQTLPQLNIMKHEVVIWLGDLNYRLCMPDANEVKSLINKDLQRLLKFDQLNIQRTQKKAFVDFNEGEIKFIPTYKYDSKTDRWDSSGKCRVPAWCDRILWRGTNVNQLNYRSHMELKTSDHKPVSALFHIGVKVVDERRYRKVFEDSVRIMDRMENDFLPSLELSRREFVFENVKFRQLQKEKFQISNNGQVPCHFSFIPKLNDSQYCKPWLRAEPFEGYLEPNETVDISLDVYVSKDSVTILNSGEDKIEDILVLHLDRGKDYFLTISGNYLPSCFGTSLEALCRMKRPIREVPVTKLIDLEEDSFLEKEKSLLQMVPLDEGASERPLQVPKEIWLLVDHLFKYACHQEDLFQTPGMQEELQQIIDCLDTSIPETIPGSNHSVAEALLIFLEALPEPVICYELYQRCLDSAYDPRICRQVISQLPRCHRNVFRYLMAFLRELLKFSEYNSVNANMIATLFTSLLLRPPPNLMARQTPSDRQRAIQFLLGFLLGSEED |
| HsINPP5E | MPSKAENLRPSEPAPQPPEGRTLQGQLPGAPPAQRAGSPPDAPGSESPALACSTPATPSGEDPPARAAPIAPRPPARPRLERALSLDDKGWRRRRFRGSQEDLEARNGTSPSRGSVQSEGPGAPAHSCSPPCLSTSLQEIPKSRGVLSSERGSPSSGGNPLSGVASSSPNLPHRDAAVAGSSPRLPSLLPPRPPPALSLDIASDSLRTANKVDSDLADYKLRAQPLLVRAHSSLGPGRPRSPLACDDCSLRSAKSSFSLLAPIRSKDVRSRSYLEGSLLASGALLGADELARYFPDRNVALFVATWNMQGQKELPPSLDEFLLPAEADYAQDLYVIGVQEGCSDRREWETRLQETLGPHYVLLSSAAHGVLYMSLFIRRDLIWFCSEVECSTVTTRIVSQIKTKGALGISFTFFGTSFLFITSHFTSGDGKVAERLLDYTRTVQALVLPRNVPDTNPYRSSAADVTTRFDEVFWFGDFNFRLSGGRTVVDALLCQGLVVDVPALLQHDQLIREMRKGSIFKGFQEPDIHFLPSYKFDIGKDTYDSTSKQRTPSYTDRVLYRSRHKGDICPVSYSSCPGIKTSDHRPVYGLFRVKVRPGRDNIPLAAGKFDRELYLLGIKRRISKEIQRQQALQSQNSSTICSVS |
| HsINPPL1 | MASACGAPGPGGALGSQAPSWYHRDLSRAAAEELLARAGRDGSFLVRDSESVAGAFALCVLYQKHVHTYRILPDGEDFLAVQTSQGVPVRRFQTLGELIGLYAQPNQGLVCALLLPVEGEREPDPPDDRDASDGEDEKPPLPPRSGSTSISAPTGPSSPLPAPETPTAPAAESAPNGLSTVSHDYLKGSYGLDLEAVRGGASHLPHLTRTLATSCRRLHSEVDKVLSGLEILSKVFDQQSSPMVTRLLQQQNLPQTGEQELESLVLKLSVLKDFLSGIQKKALKALQDMSSTAPPAPQPSTRKAKTIPVQAFEVKLDVTLGDLTKIGKSQKFTLSVDVEGGRLVLLRRQRDSQEDWTTFTHDRIRQLIKSQRVQNKLGVVFEKEKDRTQRKDFIFVSARKREAFCQLLQLMKNKHSKQDEPDMISVFIGTWNMGSVPPPKNVTSWFTSKGLGKTLDEVTVTIPHDIYVFGTQENSVGDREWLDLLRGGLKELTDLDYRPIAMQSLWNIKVAVLVKPEHENRISHVSTSSVKTGIANTLGNKGAVGVSFMFNGTSFGFVNCHLTSGNEKTARRNQNYLDILRLLSLGDRQLNAFDISLRFTHLFWFGDLNYRLDMDIQEILNYISRKEFEPLLRVDQLNLEREKHKVFLRFSEEEISFPPTYRYERGSRDTYAWHKQKPTGVRTNVPSWCDRILWKSYPETHIICNSYGCTDDIVTSDHSPVFGTFEVGVTSQFISKKGLSKTSDQAYIEFESIEAIVKTASRTKFFIEFYSTCLEEYKKSFENDAQSSDNINFLKVQWSSRQLPTLKPILADIEYLQDQHLLLTVKSMDGYESYGECVVALKSMIGSTAQQFLTFLSHRGEETGNIRGSMKVRVPTERLGTRERLYEWISIDKDEAGAKSKAPSVSRGSQEPRSGSRKPAFTEASCPLSRLFEEPEKPPPTGRPPAPPRAAPREEPLTPRLKPEGAPEPEGVAAPPPKNSFNNPAYYVLEGVPHQLLPPEPPSPARAPVPSATKNKVAITVPAPQLGHHRHPRVGEGSSSDEESGGTLPPPDFPPPPLPDSAIFLPPSLDPLPGPVVRGRGGAEARGPPPPKAHPRPPLPPGPSPASTFLGEVASGDDRSCSVLQMAKTLSEVDYAPAGPARSALLPGPLELQPPRGLPSDYGRPLSFPPPRIRESIQEDLAEEAPCLQGGRASGLGEAGMSAWLRAIGLERYEEGLVHNGWDDLEFLSDITEEDLEEAGVQDPAHKRLLLDTLQLSK |
| HsHIP | MDRMASSMKQVPNPLPKVLSRRGVGAGLEAAERESFERTQTVSINKAINTQEVAVKEKHARTCILGTHHEKGAQTFWSVVNRLPLSSNAVLCWKFCHVFHKLLRDGHPNVLKDSLRYRNELSDMSRMWGHLSEGYGQLCSIYLKLLRTKMEYHTKNPRFPGNLQMSDRQLDEAGESDVNNFFQLTVEMFDYLECELNLFQTVFNSLDMSRSVSVTAAGQCRLAPLIQVILDCSHLYDYTVKLLFKLHSCLPADTLQGHRDRFMEQFTKLKDLFYRSSNLQYFKRLIQIPQLPENPPNFLRASALSEHISPVVVIPAEASSPDSEPVLEKDDLMDMDASQQNLFDNKFDDIFGSSFSSDPFNFNSQNGVNKDEKDHLIERLYREISGLKAQLENMKTESQRVVLQLKGHVSELEADLAEQQHLRQQAADDCEFLRAELDELRRQREDTEKAQRSLSEIERKAQANEQRYSKLKEKYSELVQNHADLLRKNAEVTKQVSMARQAQVDLEREKKELEDSLERISDQGQRKTQEQLEVLESLKQELATSQRELQVLQGSLETSAQSEANWAAEFAELEKERDSLVSGAAHREEELSALRKELQDTQLKLASTEESMCQLAKDQRKMLLVGSRKAAEQVIQDALNQLEEPPLISCAGSADHLLSTVTSISSCIEQLEKSWSQYLACPEDISGLLHSITLLAHLTSDAIAHGATTCLRAPPEPADSLTEACKQYGRETLAYLASLEEEGSLENADSTAMRNCLSKIKAIGEELLPRGLDIKQEELGDLVDKEMAATSAAIETATARIEEMLSKSRAGDTGVKLEVNERILGCCTSLMQAIQVLIVASKDLQREIVESGRGTASPKEFYAKNSRWTEGLISASKAVGWGATVMVDAADLVVQGRGKFEELMVCSHEIAASTAQLVAASKVKADKDSPNLAQLQQASRGVNQATAGVVASTISGKSQIEETDNMDFSSMTLTQIKRQEMDSQVRVLELENELQKERQKLGELRKKHYELAGVAEGWEEGTEASPPTLQEVVTEKE |
| HsINPP5A | MAGKAAAPGTAVLLVTANVGSLFDDPENLQKNWLREFYQVVHTHKPHFMALHCQEFGGKNYEASMSHVDKFVKELLSSDAMKEYNRARVYLDENYKSQEHFTALGSFYFLHESLKNIYQFDFKAKKYRKVAGKEIYSDTLESTPMLEKEKFPQDYFPECKWSRKGFIRTRWCIADCAFDLVNIHLFHDASNLVAWETSPSVYSGIRHKALGYVLDRIIDQRFEKVSYFVFGDFNFRLDSKSVVETLCTKATMQTVRAADTNEVVKLIFRESDNDRKVMLQLEKKLFDYFNQEVFRDNNGTALLEFDKELSVFKDRLYELDISFPPSYPYSEDARQGEQYMNTRCPAWCDRILMSPSAKELVLRSESEEKVVTYDHIGPNVCMGDHKPVFLAFRIMPGAGKPHAHVHKCCVVQ |
| DrINPP5B | MDQSVAIQETLAEGESCTIAVECVSLLNDVRDSKLIGLVRQNKEHALFIYSHRRMAITGEDVTLGQIIPISYDFSVVEVSSPDELAVVGADTRVRVTFLEEELELKLPFGSHTRLFLSEVNRAWSDVCQQYPKEEPKFDWLTKYRKVSKGARSLRKSIGATTVKMSQMKKAERAHTANSSALKKEDESARGDALQSQEKVKGEVKDDLIRNSQPVLSNKAQMLGMPQFGLRDNLIKCELLKNEDAYTYIENYSFFLGTYNVNGQTPKESLSPWLASTASPPDFYLIGFQELDLSKEAFLFNDTPKEPEWMLAVYKGLHPDAKYALVKLVRLVGIMLLFYVKAEHAPHISEVEAETVGTGVMGRMGNKGAVSIRFQFHNSDICVVNSHLAAHTEEFERRNQDFKDICRRIQFRQEDPTLPPLTILKHNIVLWLGDLNYRISDLEVDHVKDLISKKDFETLHTYDQLKRQMDEEVVFVGFTEGEIDFQPTYKYDTGSDQWDTSEKCRVPAWCDRILWRGKSIKQLHYQSHMTLKTSDHKPVSSLLEIGIKVVNEESYKRTFEEIVRQIDRLENDCIPSVSLSEREFHFQDVKFMQHQARTVTVHNDGQVPCQFEFIQKLDEPAYCKPWLTANPAKGFLAQGASVDIDLEVFVNRHTAPELNAGLQQLEDILVLHLERGKDYFISITGSYLPSCFGSSLSALCLLREPIQDMPLESIRELSVKSNSPVIDSADKPQEIPKEIWMMVDHLFRYAKKQEDLFQQPGLRSEFEEIRDCLDTGCLDTLPGSNHSVAEALLLFLDALPEPVIPFSFYQQCLDCCSDSSHCRQIISMLPQCHKNVFNYLTAFLQELLRHSAYNRLDVNVVAPIFAGLLLRSPDKQDINEKRKVKEFFQHFLVQTSSDRDIHEKSPE |
| DrSYNJ1 | MAFSKGYRIYHKLDPPPYSVIVETRNREECLMFESGAVAVLSAAEKETIKASYTKMLDAYGILGVLRLNLGDSMLHSLVVVTGCSSVGKVQDSEVFRVTGTDFVSLKNDPTDEDRIADVRKVLNSGNFYFAWSSTGVSLDLSLNAHRRIREDTSDNRFFWNQSLHLHLKHYGVNCDDWLLRLMCGGVEIRTIYAGHKQAKACVISRLSSERAGTRFNVRGTNDDGQVANFVETEQIIFLDDKVSSFIQIRGSIPLFWEQPGIQVGSHRVKLSRGFEANAPAFERHFSALKRLYGKQLIINLLGMKEGEHMLSKAFQSHLKASEHSNAVKMLNFDYHQMVKGGKTEKLQTVLKPQISKFVEDCDFFYYSGETGIQRCQSGTIRSNCLDCLDRTNSVQAFIALEMLPKQLEAMGLTEKPQLVARFQEVFRSMWSTNGDSISKIYAGTGALDGKAKGGKLKDGARSVTRTIQNNFFDSSKQEAIDILRLGSTLNSDLADKARALLTTSSLYVSEPILQSASPRVLLGMCQNHFKYTRPKKIRVCVGTWNVNGGKQFRSIAFRNHTLNDWLLDAPKKAGHPEFQDVKNNPVDIFAIGFEEMVELNAGNIVSASTTNQKLWAAELQKNISRDQRYVLLASEQLVGVCLFVFIRPQHAPFIRDVAVDTVKTGMGGATGNKGGVAIRMLFHTTSICFVCSHFAAGQSQVKERNDDYNEIARKLSFPMGRLLYSHDYVFWCGDFNYRINIPNEEVKELIRQQNWDALIGGDQLVEQKNAGQVFRGFIEGKLDFAPTYKYDLFSEDYDTSEKCRTPAWTDRVLWKRRKWNFDKTAEELELNVVGAPVNEEEQYPWSPGDLKYYGRAELKTSDHRPVVAIIDVDILEVDPEARHQVYKEVIALQGPPDGTILVSLCSSGPDDYFDDALIDDLLDKFANFGEVILIRFVEEKMWVTFLEGYSALAALSLSGSTVNGKTIDIRLRSPGWIKSLEEEMSVERICGSIPTSTSSTLLAENSDLGEEYDMEGDVDEEVEDILPQHLQPGAGMDLSASPATSPRTSPCPSPTHGEPAPPIRPSRAPPRTAGPPQGSPVDGQPAGAPFSQGLEPKRPPPPRPNAPPARPAPPQRPPPPSGRGQATGPAPGGIPRPIPPRAGVISVTPQARPPPPAHPGAPRPTAEVHPGAPRPSPDNHPGAPRPTAEPQSKPSELPLGPPLTLPGPVRPQMTSPMQPQSVSPVQPPVQPQLPPPIQSQLPPPMQPTLPAPLMPQQAPQTSAGAGAAAAPQPGLASPKPPPRSRSSHALPPESAPAPTTQQEQSSG |
| DrINPP5E | XGPKVLQDEHSLYQPRPPSLPKPMGLGMGGKNLSFDEKVRGRRLRNSQESLTDPGETGSSTDSLKDVASANGQTLASRRPLDLQHMEMPPFRIRTGSLSETDVSPHDLCDPNKERMKPSRIVLSPLQPTGTYPLLENSTASASLRTTNRIDRDCLDYGVVGRRGRSERLHRNLSDSRLLDTMVSDNTSVHSMKSTYSVLNPIRPRDVRNRSFLEGSVLGNGALLGAEELDRYFPERRLGIYIATWNMQGEKGLPYNLDDLLLPTDTDFAQDVYVIGVQEGCPDRREWEIRLQETLGPYYVMLYAAAHGVLYLTVFVRRDLIWFCSEVEHATVTTRIISQIKTKGAVGIGFTFFGTSFLFVTSHFTSGDSKVYERILDYNKIIEALALPRNLPDTNPYRSTTSDVTTRFDEVFWFGDFNFRLNKARGDVEAILNQGVGVDMSPLLQHDQLTREMKEGSIFKGFQEASIHFPPTYKFDIGCDVYDTTTKQRTPSYTDRILYRNRQADDIRVIKYTSCSSIKTSDHRPVIGMFQVKLRPGRDNIPLGAGLFDRSLYLEGIRRRITRELKKREAVMKNQNNSTVCSIS |
| ScINP52 | MKILLSKQQTRKIAIVSETHGLVFRPINSKNSRRSTCAVELVPKAELNGNGFRRLSNHEIYGFIGLIEIEGLMFIATITGKSKVAQPIPNKTVNKIYAVDFFCLNNSKWDFMDIDSSGYPIVTNDGDFAISSPPSISTHSSRSSLRSSSSRSLNAQEQAPKHPCHELRKLLSNGSFYYSTDFDLTCTLQKRGFTEHSLSFDDFDREFMWNSFLMDEIITYRDRLDVTAKELLDQRGFLTTVIRGFAETIFSYINRLKVGLTIISRQSWKRAGTRFNARGIDDDGHVANFVETEMIMYSSQYCYAFTQIRGSLPIFWEQDTSLISPKIQITRSVEATQPTFDEHFIRLFKKYGPVHIINLLSTKSSEIQLSRRYKEQLKNSEKMKIGRDVFLTSFDFHRETSQDGFAAASRIIPKIRNTILDAGYFSYDVKEGRLISEQDGVFRTNCLDCLDRTNLIQQTISLAVFKLFLEDFRLVKPSSFIDDNEFVQKVNALWADNGDQISQIYTGTNALKSSYSRKGKMSFSGALSDATKSVSRMYINNFVDKGKQQNIDTLLGKLPHQQVVELYDPICEYVNERLLESEEKFTTHSNINLFVGTFNVNGNSRRADLSKWLFPIGDKFKPDVVVLGLQEVIELTAGSILNADYTKSSFWETMVTDCLNQYEEKYLLLRVEQMSSLLILFFARSDRAYNIKEVGGSTKKTGFGGITGNKGAVAIRFDYGATSFCFVNTHLSAGASNIDERRNDYNNIYRNITFPRSKTIPHHDSLFWLGDLNYRITLTNDEVRRELRAQKDGYIDRLLQYDQLTQEINEGVVFQGFKEPTLQFRPTYKYDYGTDNYDTSEKARTPSWTDRIIYKGENLHPLAYSDAPLKISDHKPVYAAYRANVKFVDEKEKLNLVEKLYAEYKNTHPEALTTGPDELSHARMEKQKESIPLDATVQSAGIKLIDLDDTSSCVSPLLSGPSPQPSVVGPGGLSNVSPDKSKLNVLPPPPPTSRHNKEPSSKLLSPTKEISIVSVSPRKGESNLPALERHSTPKPLPPVPALSLSKPVSLQKSSSELQHAKETIDNGKIVPRPCPPIRRKSSTAPDEISTSTKNSGVSTTEDPEPAKASTKPEKPPVVKKPHYLSVAANKLNTSQEHSIKVSPSNSKSEEELPCKKKSKPKVPAKNPELEKLSVHPLKPCDPN |
| ScINP53 | MIIFVSEEPERRLAIVSNLYALVLKPVGKKPSDKPLCAIELLQKNDLKKYGFKRLTSHEIFGVIGLIEVNGLLFVGAITGKSKVAQPCPGETVNKIFAVDFFCLNDNSWDFIEIDSSGYPVLPETASTEYQDALPKHPCYELKKLLSNGSFYYSSDFDLTSTLQHRGYGQHSLSTDTYEEEYMWNSFLMQEMITYRDHLDTNLKQILDDEGFLTTVIRGFAETFVSYVKKLKVALTIISKQSWKRAGTRFNARGVDDEANVANFVETEFIMYSSQYCYAFTQIRGSIPVFWEQGTSLINPRVQITRSFEATQPVFDKHIMKSVEKYGPVHVVNLLSTKSSEIELSKRYKEHLTHSKKLNFNKDIFLTEFDFHKETSQEGFSGVRKLIPLILDSLLSSGYYSYDVREKKNISEQHGIFRTNCLDCLDRTNLAQQIISLAAFRTFLEDFRLISSNSFIDDDDFVSKHNTLWADHGDQISQIYTGTNALKSSFSRKGKMSLAGALSDATKSVSRIYINNFMDKEKQQNIDTLLGRLPYQKAVQLYDPVNEYVSTKLQSMSDKFTSTSNINLLIGSFNVNGATKKVDLSKWLFPIGEKFKPDIVVLGLQEVIELSAGSILNADYSKSSFWENLVGDCLNQYDDKYLLLRVEQMTSLLILFFVKADKAKYVKQVEGATKKTGFRGMAGNKGAVSIRFEYGATSFCFVNSHLAAGATNVEERRSDYESIVRGITFTRTKMIPHHDSIFWLGDMNYRINLPNEDVRRELLNQEEGYIDKLLHFDQLTLGINSGSVFEGFKEPTLKFRPTYKYDPGTGTYDSSEKERTPSWTDRIIYKGENLLPLSYSDAPIMISDHRPVYAAYRAKITFVDDKERLSLKKRLFTEYKQEHPEEPGSLISDLLSLDLDNKSTDGFKSSSESSLLDIDPIMAQPTASSVASSSPVSSASASLQPVRTQNSSQSRTPIKKPVLRPPPPPAHKSVSAPAPSTSKEKSPTPQTSTASLSSVTKNIQENKPLAQNRRIPPPGFSQNILTPKSTSNLASPMSSKVDLYNSASESTRSAQDARQQTPTAFAASRDVNGQPEALLGDENPIEPEEKAKLNHMTLDSWQPLTPK |
| ScINP51 | MRLFIGRRSRSIVISSNNYCLSFQRLRSIPGASSQQRQLSKTPSVTIKSYPDTDLSSDSNYLEVKSCIFNGLLGLVCLNGDIYVAVISGVQNVGFPRWKLIDHQVRPSESIYKVLDVDFYSLENDVFDYLLCERSEQNYDKLIHEHPCGPLKKLFSDGTFYYSRDFDISNIVKNHGLSHNLEYTVDNQDLSFIWNANLASEVINWRSKISNEEKQLFANAGFLTFVIRGYCKTALIEDGPNTASITIISRISTESKQDTLELEGISEDGRVSLFVETEIVVTTEKFIFSYTQVNGSIPLFWESVESQLLYGKKIKVTKDSIEAQGAFDRHFDNLTSKYGVVSIVNIIKPKSESQEKLALTYKDCAESKGIKITNIEYSSSVLTKSPHKLLYLLKQDIYEFGAFAYDISRGIYFAKQTGVLRISAFDSIEKPNTVERLVSKEVLELTTNEIDVFELTSPFLDAHDKLWSENYYWLDRTYTKHTKNSGKYTKVYSKLFGSRVRLYDPLHIYISQYLKQLRSKYTFEKDISIFAGTFNISGKIPKDDIKDWIFPKSMSKEDEMADLYVIGLEEVVELTPGHMLATDPYVRQFWEKKILTLLNGPGRKKKYIRLWSTQLGGILLLLFMNETEYSKVKHIEGDVKKTGFGGMASNKGAVAVSFKYSATRFCVLVSHLAAGLENVEQRHNDYKTIAKSIRFSKGLRIKDHDAIIWMGDFNYRILMSNEDVRRKIVSKEYASLFEKDQLNQQMIAGESFPYFHEMAIDFPPTYKFDPGTKNYDTSEKMRIPAWTDRILSRGEVLEQLEYKCCEDILFSDHRPVYAIFRARVTVVDEQKKTTLGTQIYEKIMERLEGLDDDEKIAVLSDDAFVIESFEGSDSIAGPTHSPTPIPEPKRGRKLPPPSSDLKKWWIGSGKQVKVVLDVDPAVYMINPKRDPNPFVENEDEPLFIER |
| ScINP54 | MNKTNWKVSVTTFNCGKEFPVENSKAIVKQLLFPYDDGISQLELQDLYVLGFQEVVPIWQGSFPAVNRDLIDRITTTAVNCLNEKVSATQGDEQYSCLGVNSLGAITIIVLYNNNALKVKDDILKRNGKCGWFGTHLKGGTLISFQMTRNGEENWERFSYICAHLNANEGVNNRNQRIDDYKRIMSEVCDSEVAKSDHFFFLGDLNFRVTSTYDPTTNYSSTTTLRRLLENHEELNLLRKGEDEPLCKGFQELKITFPPTYKFKLFEKETYNTKRIPSWCDRILYKSYAVPTFAQEGTYHSVPRSNALLFSDHQPVNLTVRLPRSTGTPVPLSLHIEKYPLSWSSGLIGQIGDAVIGYCGWLVTKNVHYWILGSLLLYLLLKIL |

Figure 5 The amino acids of 5PTase genes.

The 5PTase genes of Arabidopsis, Oryza sativa, Glycine max(see above table).

| Ppa022G032500 | MQDRQPEFWPRQLLKKWISFRETGDDFERDCETDADEFSESEEFEDTDGVGESGDEDTRRLLDSDFETDSAQHADVSNKNPLPRVQSETLREQFVVNNEYKIAVGTWNVGGLLPPEDINLDGFLDSSDPADIYVLGFQEIVPLNSNNVLCVEDDHPTVVWDGLIRQALNNGVKCCERPHRSCSAPTSPRWNVKEDISDVPEVSDVNGLLNTAVAEATLESTLLFQKFPNPSDNHWPLERTALKNVDQSLKRVTKNVKSSEAWLYEATVSDDVTRGSLLVNSSPTPTKHSCNRYVRIASKQMVGIFISVWVRTELRRYVNNVKVCVVGCGILNFLRNKGAVSVSMCLHQTSFCFVCTHLTSGHKEGDELRRNADVADVLRRTTFPRLVKLSGVKLPETIMGHDRIIWLGDLNYRIDLPDLETWILVNQSDWKSLLPKDQLKVERDAGRVFQGWHEDAISFPPTYKFVVESDEYFGEDTFKGDKRRTPAWCDRILSHGQGLAQLSYLMVDAKLSDHRPVIAKFMAEVEAVSGRKLREVCRHSNDAKVNVEELLPRILPVSRFHSLHNHEHIEHSRINSSQTSIIGANIC* |
| --- | --- |
| Ppa019G072600 | MRVQGCWPIQNQTQMRHHLQVNFNDIAVYIISVGTWNVGGLLPPDDINLDGFLDCPNPADIYVLGFQEVVPLSTNNVLCV  EDDAPTVIWDGLIRQALNNRVKCCERPHRSYSEPASPMWNENEAISDVPEISEHAGLVFTADPIESSLLPQKILNSSDSQ  WLLGKIRSSSMNRTVKSLTEHVKVAEDWLCETSMPGDNVTEESLPFNSSRTLIEHSCNRYSRLASKQMVGVFISVWIRSD  LRRYVNNVKVSVVGCGILNFLRNKGAVSVSFCLHQTSFCFVCTHLTSGHNDGDEFRRNADVDYVLRRTTFPRLEKSLGVQLPETILAHDRIIWLGDLNYRIDLPDMETWALVNQCDWKSLLARDQLKMERNAGRVFKGWHEDVITFPPTYKFVVESDQYFGEDTFKGDKRRTPAWCDRILSYGQGLTQLSYSMIEAGLSDHRPVIAKFIAGVEAVSCRKLRKACRHSNDAKVNVEELLPRTFPASRFHSSQNHEIVLFDITR* |
| Ppa017G061000 | MEGSKLSKIAEALPNYPAETIPKFEEGSQVGSFRVGIGKKTDHRRNSKVHKLSHSGGSNGRTFDLEDIASGSNGSHVTST  DRACRVTVTIVAAKDLKRSGTAPDARDPVFKVRVEQTMRKSKDVKTGVDQDGILNVNQSFTFDVRELKTAQITLQVVARG  VLGIEEALGHINPLCVLNLLEEQNDRKDVETKWYDLYSKGGNRLMPGKLQMQIVVGMAEPEQTISAFVGTWNVGNARPPADLSPWLPTDAFFEIIAIGAQECDYPPRAPFTECSKDWTHTLKSHFGDRYKLVHATSRGQMRLVVFVRDDAEKAISEVDSDSEATGVGNVIANKGAVCIAFKFWDTGLCFVNCHLAAHVGQCETRNSNFKQIAMSMKVGLDSMDLLSQFHHIFWLGDLNYRLDFGKLDPQGLTPDRSFWATIVKQIHQNRWKELLKYDELRKEKGASRVLAGFKEGEITFPPTFKMQRDYSDHYDQKRMPAWCDRVLWKTLQGCHSYLISYFSAPSILTSDHKPVGATYKLTSYALPSSTLIPDGSDEDDKRWHIRFTSLRAKKLRASDINGFSDPFVSFVGPNLLQEFCSKVKHQTLNPVWNPLQELPTLVLSTFPLQRVDKEYLLVRVLDHDSDEDALGYGVIPLGQAVACFKKGVTEVAHFKVNLSHHGLPAGTLEGGMKLTWEKNVIKSKGFAGEFVSRGTSIKDSLKKKIFVRRHSPR* |
| Ppa027G015300 | MWPEASRGRPEFWPRQLVKKWLNLRNTGNIKFDRDTDMDVEHTVEEESDFEELDYFDTHTPDFHKNVDLNDVSVAPLSNPLSLLRGQSETLRQQFVDYNEYRVAVHTWNVAGKPPPDHLDLEEWIDNSQPADIYVFGFQEIVPLNANNVVLVEDQEPAARWESKIREYLNKKVGVRKEDDPFQSRSAPLSPRNDDMGISVTDVSDVEKMLGRCVSGKNLETAFLATEGNLLNYEKNIPAETLERLGEKETLTDSSWKFERESLDFTTQDLHEAAMVDERENIVLPLMSSTGSYAGYNPAGNQYQYSRVASKQMVGIFITVWIRSQLWRHVHNVKVSAVGLGLMHYLGNKGSISVSMCLHHTSFCFVCSHLTSGHKQGDQFRRNADFMEILRRTKFPRLVKVFNVELPETILAHDRIVWLGDLNYRLALSDKETWQLVSCRDWESLLRKDQLKLEQGEGRVFKDWMEGPIHFPPTYKYKEGTDQFSGETSSTGEKRRSPAWCDRILWYGKGMRQVAYTRGDLKLSDHRSVCATFIAEVEVVSHRKLKKACIYPKNIKLIDLETEKGKINAPSSNALQRKRSHKFRNLGRASSFVYPESSVSSARSWKGDLSRREERFGGFPVPVSGYS* |
| Ppa021G072300 | MTFNRKYAATETALSCHKNLYSNCFDPCFPLPSELKLIDPKIVEKLGAERKFSKGAAVVPEVPKPLIGDRVIRDAKQTGVKQVISAKEKSFPRGGETAPSTAADLNVYVVTWNMNGKVPNNFADLFDVSGESHDFFVVGLQEAPNFDAKTCISEILGDKYCLVESSVLMSLQLFIFSKRSLKPFISGVKVDKVWEKGLSGIVGKQKGAAAVRLHFGDKSFLFITSHLAAHESNLKARNAQCAHICHSLFARSSVYSCFQPGTNTDDLNYRVPGVPSNVVEESDVVIWLGDLNYRLELPRRFVQNSIKNKNLQELWAKDQLSVSLRKGQAFKGFHEGPLLFAPTFKYDVGTDNYDTGPKERVPSWTDRILYKTTNVKAELRSYDAISSVKTSDHRPVEAHLTFKNLL* |
| Ppa021G021500 | MQGSLSELWPRKLLKKWLSFQETGDDFDRDSDSSGGECSESEDFEDSDTFKCKKQLMRVQSDILREQFVQNDEYKVAVGTWNVGGLLPPDDIDLTEFLDTSDPADIYVLGFQEIVPLNINNVLCVENDAPTVVWDGLIRRALNNTMKCCKKCLRSHSVPPSSMWHEKEVANQDLEDSDSLGLRNVVATEETLETSLLLQKFPNSSDFYPSKDKTEARKSDHTLKSFTKNVKLPEDWLCEVTTPDNGATEQTSLEKYSQVLRKHSCDQYFRVASKQMVGVFISVWVRSDLRRYVHNVKVSVVGCGILNFLRNKGAVSVSMSLHQTSFCFVCTHLTSGLKEGDEFRRNADVADVLRRTAFPRLVKLSGIQLPETIMAHDRIIWLGDLNYRIDLPDKETWILVNQCDWKSLLPRDQLRMERDAGRVFKGWHEDAISFPPTYKFVEESDQYFGEYTFKGDRRRTPAWCDRILSHGKGLRQLSYSMVQARLSDHRPVVAKFIAEVEAVNSRNFKKAFKLPISKVNVEEYLPKAFSVCKFCSLHNHEIVEFI* |
| Ppa001G125600 | MEESTLSNDGESSPTHAEGGVSKLEGISQTGSSRVGVWKKLEHRRSRIQKSSHAGGGNGRTFDLENIASGSNGSHVTNTDRACRVTVTIIAAKDLKRTTPGPDARDPVLKVRVEQTLKKSKDIRTGVHQDGTLDVNQSFTFDVRELKSAQITLQVVARGVLGIEEALGHINPLYVLNLLEPFCSRNQIGGLRGKTVARET* |
| Ppa001G133200 | LLSLIRLTHLNGEQILVLSEELQIDISEGKVDGSLKLSDSIVAADSKTKKTFVGDMATTPFQGTPRRLRGQSETLRQQFIDTHQYRIDVHTWNVAGKPPPDDLDLDGWINTSNPADIYVFGFQEVVPLNANNVLCVENDCPAALWEAKIRKTLNKICGQPKSSGLVKCLSVPIPSPSNPIDVIVTDVPEVSDVDRLLAEVASPRFQGNSQSSPTSEESFVHTNKNPTNCKHSVAKLSLERYPSQNGASIAASSSPWNGFSTEHPHQYLRVASKQMVGLFIIVWIRSSLWRQVHNVQVSTVGCGLMNHLGNKGAVSVSMFLHHTSFCFVCSHLTSGHKEGDALRRNADVAEILRRTRFPRLAKLLGLQLPETILAHDRVIWLGDLNYRLALPDKETWTLVKQGDWATLLRVDQLKLEQSEGRVFPDWQEGPIYFPPTYKYMNGSDNYSSGEGSTGSSSKRRSPAWCDRVLWYGQGLHQLNYSREDCKLSDHRSVSATFNVDVEVVSQNKLRRVVKHEALDDEEVVQSEEPNCGNRTSN* |
| Ppa018G042500 | MQGTQSEFWPRQLLKKWISFQETGDDFNRDSDSNSERFSESEDFEESDDINYSNPIPRVQSDTLHERFVQNNDYKVAVGTWNVGGLLPPDDIDLDGFLDSSDPADIYVLGFQEIVPLNASNVLCIEDDAPTVIWDGLIRQALNNRVKCFEEHVTSHSAPTSPTWDEQEFVSPSSEVSDFESLRTTVATEGTLESSLLLPKISNPSDSQWPSDRIEARKFVHKVHAPIKDVRVAEDWVYEASMPDNGVTRRMSSMKSSRNLRTHSCNKYLRVASKQMVGVFISIWVRSDLRRYVHNVKVSVVGCGILNYLRNKGAVSVSMSLHQTSFCFVCTHLTSGLKEGDEFRRNADVADVLRRTTFPRLDKLSGIHLPETIMAHDRIIWLGDLNYRIDLPDKETWILVNQCDWKSLLPRDQLSIERDAGRVFKGWHEEVINFPPTYKFVEESDKYFGENTFEGDKRRTPAWCDRILSYGKGLRQLSYSMVEARLSDHRPVIAKFFVEVEAVNCQKLTKACKLSKHAKVNVEELLLKDFSVNNIRSLFNNEML* |
| Ppa018G003200 | MGNCVGRPDDYVLHHGRSKWIVKYGQQVVRGLLDCEKSNLMAFNIKYAATDTALSCHRNLYRSCFDPCFPLPSDYKLIDPKIVEELGVERKPPKAALATPGVPKPPNGGKRMRREAEKSGTKQVISAKEIASWKSKTVASTAADLDVYVVTWNMNSKVPSNFSDLFDLSGDGHDFFVVGLQEAPMFDAKTSISEILGAKYCLVESSVLMSLQLFIFAKRSIKPFISGVKVDKVWEKGLGG  IVGKLKGAAAVRLQFGDKSLLFITSHLAAHESNLKTRNAQCARIHQSMFARSTSVYSCFQPGTNTEDLSCRVRGLPSNVV  EESDVVIWLGDLNYRVELPRSVVQASIKHNKLQELWPKDQLSVALSKNQAFKGFREGPLLFAPTFKYDVGTDMYDTSPKE  RVPSWTDRILYKTSTVKAELRSYDAIGSLKTSDHRPVKAHLTFKNLL* |
| Ppa016G008900 | MNESIKTALCAGSSLWAAFDHGLKVWDIEDASSGGSEGSNNSPGDEDAAAYIPLVVNSGATMCLAMDNANQIIWSGHRDGRLRAWPLNIRDGGSQGRDQILEWDGHQAPVTAITITSYEEGTVWGGFGNGHLVKWDSEGTRQQWEPLAAVAVKCLLVVGSRLWVGYANGKIEVLSRNLKSEGCWPAQTSSIVHMARGGNYVFTLSADGGIRGWHVASLSPLDSLIQDELSKRTDNFTQKRQLRIFAGTWNVSQEKATSGSLRAWLEKSAADASLVCIGLQEMEMGAGSIGLAAVKETMGVGLLDKGSANGQWWLDKIGSIIGEGKDFERVASRQLAGLLIGVWVKKSIRQFVGDFDAAAVACGFGRTFGNKGAVGVKLSAFRRTICLVNSHFAAHMEKVNSRNSDFEYCYNQMAFGPKPVPTAVGGNGLLARGGSRKLSSSTEIQEILREEGMPSLAEQDFHSEMPMPELSDTDILVWFGDFNYRIDTTYDQAIKWISEQRYDMLLIRDQLRVEMTSGRTFPGMREAGITFPPTYKFDRGSQVYDTSEKKRVPAYCDRVVFRDSFDGSVSATSTSLTNPAQATSIRYDACMEALDSDHKPVRCVLDVDLAVLDEAARRREFGDILKNDEKVLNFLEQTNVIPQTRIDKNLIVLGNSKSSLSVINESKQSWTTYTVLCEGLYVGEDCPCGNHRANKGANRENMLRSGNGFPLWLQVQPAAGIIRPNSSVTITMEYRVPDQQNNFSWKRSNSQPRWWNDVQKDDCLKLVLTMSSSALTKVNQHRICVRKLR* |
| Ppa002G039700 | MDGSNVSTIGDGLPADTESSISKLEDIPQGGPFKIGLGKKLESRRSRVQRLPHVGGGNGRTFDLEDIASGSNGSHVTSTDRACRVTVTIIAAKDLKRSTIGPEARDPVFKVRVEETVRKSKDLKTGVNQDGTLDVNQSFTFDVRELKSAQITLQVVARGVLGIEEALGHINPLCVLNLLEEQGGRKDVEAKWYDLYSKSANRIMPGKLHMQIIVGMADPEQTISAFVGTWNVGNARPPPDLSPWLPTDAFFEIVAIGTQECDYQPRAPFTECSKDWIHTLKSHFGERYKLVHATSRGQMRLVVLVRDDAEKAISEVDSDSEATGVGNVMANKGGVCIAFKFWDTGLCFVNCHLAAHVGQCETRNSNFRQIAMSMRVGLQSMDLLSQFHHIFWLGDLNYRLDFGNLEPQGLTPDRTFWATIVKQIHANRWKDLLKYDELRKERAASRILAGFKEGEITFPPTFKMQRDYLDHYDQKRMPAWCDRVLWKTLQGCHSYLISYFSASSIVTSDHKPVGATYKLTSYALPSSTFVPDGTDEDDKRWHIRFTSLRAKKLRASDINGFSDPFVSFVGPNLLQEFHSKVKHQTLNPVWNPFQELPTLVLSTFPLQRVDKEYLLVRILDHDSDEDALGYGVIPLGQAVACFKKGFLEVAQFKVNLTHHGLPAGTLEGGMKLTWEKNAIKKKDYSVDFSSRGTSIRDSLKKKIFVRRPSPK* |
| Lj02.CM0021.750 | MKARRGKRSEAFWPSMVMKKWLNIKPKVNDFSEDEVDTETESEDDACSPKASRMAACEDNPLRTQRTQSILQSQISSASCKGYKTRHRRGKSETLRAQYIKTKEVRVTIGTWNVAGKVPSLDLEIDDWLCTEEPADIYILGFQEVVPLNAGNVLGAEDNTPIHKWEEIIRRSLNKSSEPNSKKLSQSAPPSPVLRTSSADDVLADNMGAANPIDMMNDEYIGRTIDDFELKPREVNNIIGSNLDWPERPLDAISQIVDSNPKFRRVLSSSARIGFNLTENSLVYGGGLKRSHNSSGNLGLLWKQQQVIPEVVDSLADVCGVSPPEDDDTFLDLPDNQDDIELGGAMDTRPRYVRIVSKQMVGIYVSVWVQRRLRRHINNLKVSPVGVGIMGYIGNKGSVSVSMSLFQSRMCFVCSHLSSGQKDGAEQRRNSNVHEILRRTCFSSVFDSDQPLTIPSHDQIFWFGDLNYRISMLDAEVRKLVALKKWDELMNYDQLNKELRVGRVFDGWKEGLINFPPTYKYEINSDRYVGECPKEGEKMRAPAWCDRILWLGKGIKQLEYGRSEIRISDHRPVSSMFLVEVEVFDQRKLKIALNFTNTAAVHPAIFTDEDGDILSSY |
| Lj05.CM0052.130 | LQEIVPLNAGNIFGAEDTRPVPKWENIIRDTLNRVRSITPKMKSFSDPPSPSKFKPSEDVPNIEEEIFESDSDIGEEVHPLDEEHNNVYDGTSDKPITNSLQSSDAAVIADSEVQVKHVLQTQVSYPKSLSRLNCFRDEYLSENIETPPAQPQLSRMISGSERIGLCWPEPPLHLLPQRVLERPTSFKSFKSFNASKSFRTCQSFRQNMDDIGLLGEIDLGALMKRKRRSSYVKLVSKQMVGIFITVWVRRSLRKHIQNLKVSTVGVGVMGYIGNKGSISVSMSIYQTLFCFICTHLTAGEKEVDELKRNADIREIHQRTHFFSDIGLPRSILDHERIFWLGDLNYRINLSYEKTRDLISKKQWSKLIEKDQLIKEIHKGVFEGWSEGVLNFAPTYKYVTNSEKYYGEDPKVGRRTPAWCDRVLSYGNGMKLLCYGRNELNFSDHRPVTATYLAEVEVFSPRKLQRALTFTDAEIENEEIMTNFGTWN |
| Lj02.CM0081.1470 | MFCFQRHDSIVGGAIYSADGAEDESDHSRGNLSLQSLEFDPCISIDKLRIFVGTWNVAGRSPVGSLAVDMDEWLNLKNAADIYVLGFQETVPLKTLTVIGAEDPSVATNWNQLIGTTLNNKFNYHQKMESPSSIAGNNLYRVVASKKMVGVLMSVWIREEVMKKYRVSNVRVSSVACGVMGCLGNKGSVAVSMLIEGTSFCFVAAHLASGEKKGDEGRRNHQVAEIFRRSSFARTTTSHHHRYPLTILGHERIFWFGDLNYRLYLKDDFARNLIRKQDWKALQEFDQLQKELEEGGVFEGWKEGNIEFAPTYKYASSPSNLYCGGGLPTRSGGKQRTPAWCDRILWYGKGVEQLQYIRSESKFSDHRPVSALFSTQIEVKSSSNGLMEPENITPTMWQPDHVSFHCFSKSYERDV |
| Lj05.CM0200.370 | WTTMTRTRSCPASTPSHRTAKPIPTANNSAAPPRTNAITKSAITASTIAESLTPLSIPSTTLTPMTTTTFTLLPLQISLPMLMIISPSISRCRSLSAAAAEPAFSRRRLERLFIPDGRRVLSYALIR*GRLRWGSFSGTLLVRRRSCGLGRKLGLGYGSFRMCMITVAVSVGG*GAATRTLHRFMNRRIPRLPIV*QLTMGAG*SGVGIKMGRLGHGKWIKNFVLHSRKVCLGRLIEVLFLPWSLVPMAISGRVPKAVFLRSGPGNLWRNLFHCLQQKGTWQLYL*RGHSLTSEVKLLLMVSAVYLLKRLSVWCLIIFEAESGVLGHYPFRYGMPVRRNF*KYLTLMVRLRIE*TCHRCSRIKQWKMK*R*SLFRPQKRRNPRALAFCRGHVMLSWELQTLSGELRRKELGHLLKILKEQKLLFRQVME*FGVVVQTAYLCSGMEVGRVCKILIAIPALFNASALLEHGCM*AMSVVLSKYWT*KAI*LQDGLLIIVL*LNWLLAMAVFLAWLLMVAYVDGILRLQVQLTA**DQSWPQRNLIIQDDTISEF*LAHGMSAKVELPKIHFCLGWVPLYQM*ALL*LVCKKWRWVLVFLQCLQQKKL*VLKEVQWGSGGLIQLERP*KKEKLSSEWVLGSLLACLFLFG*ERILGNMLVTLMLVQSHVVLDVQLVIREGWV*ESESMTG*CAL*IVTWLHIWKQLIGEMLILITFIEIWSSADHPTYLITQPAMGNSSEEVKPELSDADMVVFFGDFNYRLFGISYDEARDFVSQRCFDWLREKDQLRAEMKSGKVFQGMREALIKFPPTYKFERHQPGLGGYDSGEKKRIPAWCDRIIYRDTRPAAVSDCNLDCPVVSSILHLSSLKPFNRYDACMDVTDSDHKPVRCKFNVNISHADRSIRRKEFGEIMTSNEKIRSMLEELAYVPEFTVIPDNLVLQNHEVSFLLITNRSSKDKAVYKITCEGQSIVKNDGQSPDYSPRGAFGFPRWLEVTPSSGIINPEQDVEVSIRHEDVHTSEELVDGIPQNWWSEDTRDKEVILVVHVQGSSSVETHSHKIHARHCFTAKPVRTDSKSNSARRNQVS |
| Lj05.CM0200.890 | MKLSWSKKMVRKFFNIKSKSEDSQADGVPCGGSDVDYRSRSSCSEREPRTIKKSKTEKFSRSADQVRRGRMNLDHPRIIDVQNYSIFVGSWNVAGRSPPSNLSLDDWLHSSPPADIYVLGFQEIVPLNAGNILGAEDNGPAKKWLSLIRKTLNNLPGTSGSSACYTPSPIPQPVAELNADFEGSARQKNSSFFHRRSFQTTSSGWGMDNDPSVMQPRLDRRYSVCDRVISGHRPSDFDPSLRWGYRSSDYSRASDYSRPSDYSRPSDYSRWGSSDDDNGLGDSPSTVLYSPMSYGGPAASNEDGYGMPGNSRYCLVASKQMVGIYLTIWVRSELKDHVQNMKVSCVGRGLMGYLGNKGSISISMSVHETSFCFICSHLTSGQKEGDELRRNSDVMEILKKTRFPRVHGVDNEKSPQTILEHDRIIWLGDLNYRIALSYRSAKALVEMQNWRALLENDQLRIEQKRGRAFVGWKEGKIYFPPTYKYSTNSDRYAGDDMHPKEKRRTPAWCDRILWYGEGLHQLSYVRGESRFSDHRPVYGRFGAEVVSTHGKMKKSMSCSRSRIEVEELLPYSGGYTELTFF |
| Lj04.CM0307.130 | MSSSTPSRYKSNATFDMTKANDIGLSPINTANSSDTSTKNEKKKKSILPKIFGSKKNGRGSSDEDALKPNAEGDGVSVSLDLEKKIESRRKAFLETSPVMRRSFSGRETSPGIESLNLSNFERPLAPETEIQSFRIFVATWNVGGKSPSYDLNLQDFLLVEGSADIYVLGFQEIVPLSAGNVLVIEDNEPAVKWLALISQALNRPKNEYSDSSDSGSKNQNSSKESKSPASLNFFQKPSLKVISKSFRAEGSSLLKACNCPMESPSRERRRIRKYSDPINKLDSEIRGGSPVEELLSIAEIPSSLSQTKYNLISSKQMVGIFLTIWSKKDLVPHIGHLRVDSVGRGIMGCLGNKGCISVSMSIHQTSFCFVCSHLASGEKEGDELKRNSDVAEILKGIQFPRICKTPCRRAPEKIFDHDRIIWLGDLNYRMALSYEETRVLLEDNDWDTLLEKDQLNIEREAGRVFSGFKEGRIFFAPTYKYSHNSDSYAGETVKSKKKRRTPAWCDRILWRGSNIEQLSYIRGESRFSDHRPVCSVFSVGVEVRSRNTRFRKGYSYTSSRLEYEDLIPQRHSFYDY |
| Lj02.CM0312.930 | MLDDYIFCLKIMQSFKLNESKILRKMLSIGNFKEGNQNSSDAKQETSSMNQAYANARRCFYHQTKRIFVGSWNIGGIEPPNNLDMEDWLDAGNNCADIYVLGFQEIVPLNAANILCPRNRRNSRKWNSLIGAALNNRRPIKGVEEDKIAEPHKIYPLQKQICAEGEHEQDFQCIRSKQMVGVFITIWVQSYLLQSIRHLSVSSVGCGIMGCLGNKGSISIRFCLHETSFCFICSHLASGGKEEDRRQRNVNATDILSRTRFSTDTLHDMPRKIIDHDRVVWLGDLNYRIYMPDSETQSLIKRREWETLLKQDQLKKELTEGHVFRGWHEGAIEFPPTYKYHPNSKDYIGCDQQHMSKKRRAPAWCDRIIWFGKGMKQTQYCRSESKLSDHRPVRAMFKADIKVAANCR |
| Lj01.CM0591.380 | MVRKFFNLKSKAEESHQAGDVAYGGGDVDYRSRSSLSEREQCTIKKSKTEKLSRNSSQARRGKMNLDHPRIIDVHNYSIFTATWNVAGRSPPSNLNLDDLLHASPPADIYVLGFQEIVPLNAGNILGAEDNGPAKKWLSLIGKTLNNLPGSSGCNGYYTPSPIPQPVVELNADFEGSARQKNSSFFQRRSFQTTSSNWGMDNDPSTALPRLDRRFSVCDRVIFGNRRSDFDPSFRWGYRPSDYSRASDYSRPSDYSRMGSSDDDNGLGDSPSTVLFSPMSYGGPASAEDVYGVQGRPRYCLFASKQMVGIFLTIWVSELKDHVRNLKVSCVGRGLMGYLGNKGSISISMSLHETSFCFICSHLTSGQKEGDELRRNSDVMEILKKTRFPRVHGADNEKSPETILEHDRIIWLGDLNYRIALSYRTAKALVEMQNWRALLENDQLRIEQKRGRAFVGWNEGKIYFPPTYKYSTNSDRYAGDDMHPKEKRRTPAWCDRILWYGEGLHQLSYVRGESRFSDHKPVYAIFWAEVESNHGRLKKSMSCSRSRIEVEELLPYSHGYTELSFFEGEEDDFFYS*FFNYFWRIFMMTE*S*SMDKIIIC |
| Lj01.CM2079.230 | MRTESRKSKSSWLAVRKWLNVKSNSDAKFHSDNCRVTERKNSCSDQDSNAFVPDHFSDWLKDSTNGMKGSAVGEKETSHVTTNAMNLRMFAGTWNVGGKSPNEGLNLKSWLTSHSPADIYVIGFQEIVPLNAGNVLGPEDSGPASKWLALIREALNTNDIIDTETTSPLTSYLTSDSDSRHYCQVTSKQMVGVFLSVWVRADLCNHLTNLKLSCVGRGIMGYLGNKGSISISMTLYNTTFCFVCTHLASGEKFGDELRRNLDVSEILKKTKFCHSFKSLLHPQTILEHDIIIWLGDLNYRLAAGYDDTHELLKKNDWEALLEKDQLRIEQRAGRVFKGWNEGSIYFAPTYKYLTNSDQYVAQTSKSKEKPRTPAWCDRILWKGEGLNQMWYVRGESKFSDHRPVSSLFSVQVDKSSKNLTPSAATVTRSCSLKPLTSAAFSSTCAAKVQAEEQLMLLTRAQSCIVSAPR |
| LjT39F13.100 | MWPILVANKILNKQIGSRNFIADYPSYAEPLLGITSHDHSSLSTKSILNNHQDSQKYKVFVSTWNVGGISPDEGLNMDDLLETCNKPFDIYVLGFQEIVPLKAKSVLGSENSKISTKWNSLIREALNKRTHDNKEGNPAQQCRAPQDFNCIISKQMVGLLVSVWVRKDLHPFIQHPCVSCVGCGIMGFLGNKGSVSVRFLLHGTSFCFVCSHLASGGREGDEKYRNSNVGEIFSRT |
| LjT39F13.120 | FPRGPLLDLPAKILDHDHVILLGDLNYRISLPEETTRLLIERRDWDSLLENDQLKMELVSGNLLRGWHEGAIEFAPTYKYCLNSDLYYGCCYHAKMKAAKKRSPAWCDRIIWFGKGLKQIEYARSESKLSDHRPVKALFTAQVRDSAAIKNFPSLFLSERFQKIKTHHFQVSPTHDEFVCQKQASFHR |
| LjSGA_073368.1 | LTVSLVCYAEWGRQSRFMVNRGGEAPLESNDFVPRLRRQKSSTYRSQYINTKDLRVCAGTWNVGGELPPDDLDIDEWLGVSEPADIYVL |
| LjSGA_017225.1 | I*YPYTAKEN*PYLYIFTIRTSSF*DNKLSLMQGSVSVSMSLFQSRMCFVCSHLSSGQKEGAEHRRNSDVHEILRRTRFSSVFDADQPQTIPSHDQIFWFGDLNYRINLLDVEVRKLVAQKKWDELMNYDQLSNELHSGHVFDGWKEGFVNFPPTYKYEFNSDKYVGENPKEGEKKRSPAWCDRILWLGKGIKQLQYGRSENKLSDHRPVSAIFSVDVEVFDHRKLQRALNFTNAAVHPGVFLKDAAVHPEVFLTDGDLS |
| LjSGA_015160.1 | IVVRFSTSGLKPT*RFHVGDVDVAAVPCGFGRAIGNKGAVGLRIRVYDRIMCFVNCHFAAHLDAVNRRNADFDHVYRTMSFGRPTNLLNATAAGTLSSIPIFRGTNSAEGMPELSEADMIIFLGDFNYRLDDISYDEARDFVSQRCFDWLREKDQLRAEMEAGNAFQGMREAIITFPPTYKFERHQAGLAGWLPLPTGMILVKRNVYLPGATEFCIVIIALLRWPNAVYSVL*SLQY |
| Mtr8g043480 | MDNEFEDGQAASDMMRSNQQRKKQSFIQKVLTMRERNGRTIERGSIDSHEEISDPSIQNQGSVPSMSSCEAVQNFNVFAATWNVGGQCPSGNLDLSDFLQVRNEPDMYVLGFQEIVPLNAGNVLVLEDNEPAAKWLALINQSLNGPSDFSSNKGLKPTASFGGSLYFQKPSLKKIKKTFKKLNGKRLKSCNCILEMERKAAKDFCFRCQESNVNLDDSSTEEEDDSYPISVALATNQMKYSLVTCKQMVGIFVSVWMKKELIQYVGHLRICCTSRGIMGCLGNKGCISVSMSFYQTSFCFICSHLASGEKEGDELRRNLDVIEILKNTQFPKICKNQYSRMPDKILDHDRIIWFGDLNYRISLSRDVAKRLVEMKDWPALFNKDQLKMEREAGRVFKGWKEGKIYFAPTYKYAFNSDTYYAEGVKVSKNKRRTPAWCDRILWHGRGIQQLSYVRKEFKFSDHRPVCATFLVEVEVMFRGQKKKVSTFNFQIHDLVPTRTSYYS* |
| Mtr8g091360 | MSSSSSSSRSKSNAANSEMTKANNNISLIPIHHSYNTTNTSPDNSVKNEKKKKSILPKIFGSKRNGRGSDEDALKSNTEGDSISISFDLERKIETRRKAFLEAAPIMRKSFSERETSPGIEGLNLCNFERPMAPENEIQSFRVFVGTWNVGGKSPSYDLNLQDFLLVEGSADIYVLGFQEIVPLSAGNVLVIEDNEPAAKWLALISQALNTPKSDLSDSSDSGAGSKTKESKSPASLHFFQKPSLKAVRRSFRAEGSSLLKACNCPVESPSRERRRVRKFSDPMNKLDSEIHGESSMEELLSIAEIPASPGQSKYSLVSSKQMVGIFLTIWTKKELVPHIGHLRVDSVGRGIMGCLGNKGCISMSMTLHQTSFCFVCSHLASGEKEGDEVRRNSDVAEILKGIQFPRICKNPYRRAPEKIVDHDRIIWLGDLNYRVALSYEETRVLLEDNDWDTLLEKDQLNIERDAGRVFSGFKEGKIVFAPTYKYSHNSDSYAGETVKSKKKRRTPAWCDRILWRGRRIEQLSYIRGESRFSDHRPVCAVFSVGVEVRSRNNRFRKGYSYTSPRVEYEDFIPQRHSFYDY* |
| Mtr7g100070 | MPLTHVLITSYLRFIHLYSYSHCFFCFGKLFLSDLESGGAMKIESKKSKSWPKLAVKNWLSTKNSSEKFTSDYSVTGSATETRKSCSDQDSYILVPDNFSEGWLKHSTNGVKRSVHGEIKPSTITNPLNLRMFVGTWNVGGKSPNENLDLKNWLISTSPADIYVIGFQEIVPLNAGNVLGSENSGPAAKWLALIHQALNTSNNEIPNQKKRFSLVASKQMVGIFLCVWVRADYRNHVGNLKVSRVGTGIMGYLGNKGSISISMRLYQTTFCFVCTHLASGEKCGDELRRNLDIAEIIKRTKFSHSLGILEHDNIIWLGDLNYRLAAGYDEIHELLKNNNLKALLEKDQLRMEQNAGRIFEGWNEGSIYFAPTYKYLMNSDQYVAQTCKSKEKRRTPAWCDRILWKGEGLNQKMYVRGESKFSDHRPVYSLFTAQVDMTNKNLTRSASTTISRSCPLKPFTNSAALPSTCCAAAKVQAEEQIMLLATRTQSCIDSVSRFL* |
| Mtr7g095260 | MRDENLKKTTKLSWPKTLVKKWFNIKTKSQDFQADDVICQVVDEEYGSNYSKMEACSIKKSITERSSRTYIDRMQRGKSYLGEAQVTDVCNYRIFVATWNVAGKSPPSYLSLEDWLHISPHADIYVLGFQEIVPLNAVNVLGTEDNGPAKKWLALIRKTLNNLPGTSGGYNTPSPIPAPIVELDADFEGSMRQKATSFFHRKSFQAMSRSMRMDNEMSLPQTGLGRRLSVCDRMISGHRKSDYDTNCRWGSSDDENGAGDSPTDYSPISYSDCFPTDDSDRPRGNSRYCLVASKQMVGVFLTVWVKSDIRDDVHNMKVSCVGRGLMGYLGNKGSISISMSLRQTSFCFVCSHLTSGQRDGDELRRNSDVMEILRKTRFPHILDTTDANSPQTILEHDRIIWLGDLNYRIALSYRGAKALVEMHDWKTLLKNDQLCIEKRQGRVFTGWSEGKIYFPPTYKYSNNSDSYAGDDRRSKQKRRTPAWCDRILWYGSGLQQLSYVRGESRFSDHRPVCSIFLAEVESINSNQIKKGSTCSSSRIEIEELLPHSHGYNFHFY* |
| Mtr5g014090 | MKGRRGKRSEAFWPSIVMKKWLNIKPKVNDFSEDEVDTETESEDDVCSPKQPRMQISDDSPFRTQGTQSIFSSQISDTSFKKGCKTRHRRGKSETLRAQYINTKEVRVAIGSWNVAGRHPSEDLDIDDWIYAEEPSDIYIFGFQEVVPLNAGNVLGAEDNTPIQKWEAIIRRSLNKSSEPDSKHKSHSAPPSPVLRTSSAADVLADNIDAANPIDMLNDELMENVDKYDLQQLEESNIISIGNDLHVRKVYGIDLDWPERPLDAISQIVDSNPKLRRVLSSSARIGFDLNENAFLYGGGGGGGLKRTHHSSGNLGSLLKEQQVIPKVVDSLDDVSEMLSDDGDDAFIELPENQDDDELGTTKSQARYVRIISKQMVGIYVSVWVQRRLRRHINNLKVSPVGVGLMGYMGNKGSVSISMSLFQSRMCFVCSHLTSGTKDGAEQRRNSDVNEILRRTCFSSVFATDQALTIPSHDQIFWFGDLNYRISMLDSEVRKLVAQKKWNELLNYDQLSNELRVGHVFDGWKEGLINFAPTYKYEINSDRYVGEIPKEGEKKRAPAWCDRILWLGKGIKQLNYERAEIKLSDHRPVSSIFLVEVEVFDHRKLRRALNFTNTAAVHPEIFPDEDGQFF* |
| Mtr5g006820 | MRDENTKKSKLSWSKKMVRKFFNIKCKTEDTTQQRDGFASGGGGLEYRSRSSLSEREPSTIKKSKTEKFSRNSSQVRRARMNLDHPRIIDVHNYSIFVGTWNVAGRSPPSNLSINDWLHASPPADIYVLGFQEIVPLNAGNILGAEDNGPAKKWLALIGNALNSLPGTSGGNGYYTPSPIPQPVVELNADFEGSARQKNSSFFHRRSFQTTSSSWGMDNDPSTVQPRLDRRFSVCDRVIFGNRKSDFDPSLRWGYRPSDYSRASDSRPSDYYTRPSDSRPSDYYSRASDYSRPSDYYSRASDYSRPSDYSRWGSSEDDNGLEDSPSTVLFSPMSYGGPAASGEDGYSMPGRSRYSLVASKQMVGIFLTVWVRGELKDHVKNMKVSCVGRGLMGYLGNKGSISISMSLHETSFCFICSHLTSGQKEGDELRRNSDVLEILKKTRFPRVHGSDSVKSPETILEHDRIIWLGDLNYRIALNYRSAKALIEMQNWRALLENDQLRIEQKRGRAFAGWKEGKIYFPPTYKYSTNSDRYAGDDMHPKEKRRTPAWCDRILWHGEGLRQISYVRGESRFSDHRPVYGLFWAEVESNHGKLKKSMSCSRSRIQVEELLPYSHGYTELNFF* |
| Mtr1g021730 | MGFVHHQTSPSQIGIRTVDVDNTCNFSTTSNLCISIFTWNMNGQVSFEDLAEMVGSNRDFDLLAVGLQEAPGNKIATMLSAALNESHTLIGKVTMQSLQLYLFGPKNAKSFIQELHVDKESFGGCGGIIGRKKGAVAIRINYKGIRLLFISCHLSAHGRNVQERNYECRHVSRSLFSKIWNPYSRPAHMTIWLGDLNYRLEGINAHPARNLIDQDLHHKLHGNDQLLQQAGEGQIFNGFCEGTLTFKPTYKYNKGSSDYDTSYKVRVPAWTDRILFKIEEDTDNVEATLHSYESMDEIYGSDHKPVKAHICLRLQT* |
| Mtr1g078100 | MDPFHQNDASSSFPLSSSTPPQQQQQQRTFLYSRSSSSSSNDNDDVSSSNSIHSTNRRLDCMLQFLDRKLTISDHLPLPEFIAKGGGAGIFKPPLRAAVHPSRPPFLELRPHPLRETQIGRFLRNIVATDSQLWSATERGLRFWNFKDLYASWYGEEGLVRSGDEESAPFRESLLTSPAICLVADEGNRLVWSGHKDGKIRCWQMDSQNSDFNHKLSWQAHRGPVLSITITSYGDLWSGSEGGVIKIWPWEAVEKSIHLTEEERHKAVIFIERSYVDLRSQLSTNGYNNMLASDIKYLVSDNSKAKVWSSGYFSYALWDARTRELLKVFNSDGQMENRSDLPSMQDFPVDLVSSSRKDRNQSSIGFFQRSRNALMGAADAVRRVAAKGAFGDENRRTEALVVTIDGMIWTGYSSGLLVQWDGNGNRIQDFIYHPFAVQCFCTYGMQIWVGYASGIVQVLDLKGNLIGGWVAHSCPIVKMTVGVGYVFTLANHGGIRGWNITSPGPLDSILCSELGGKEFLYTKIENIKILSGTWNVGQGKASQDSLTSWLGSVASDVGLVVVGLQEVEMGAGFLAMSAAKETVGLEGSSAGQWWLDMIDKTLDEGSTFKRIGSRQLAGLVIAVWVKTNITLHVGDVDAAAVPCGFGRAIGNKGAVALRVRVYDRIMCFVNCHFAAHLDAVGRRNSDFDYVYRTMSFSRPTNLLNTTPAGTSASIPMFRGTNPAEGIPELSEADMIVFLGDLNYRLDDISYDEARDFVSQRCFDWLRERDQLRAEMEAGNAFQGMREAVITFPPTYKFERHQAGLAGYDSGEKKRIPAWCDRILYRDSRSSSVAECNLEYPVVSSVLQYEACMDVTDSDHKPVRCIFSTDIARVDEPIRRQEFGEILESNEKIKCLLKELYKIPETIISTNNIILQNQDTLILRITNKSTEDNALFEIICEGETTVLEDQKATNHYLRGSFGLPRWLEVSPATGIIRPDQIIEVSVHHEEFQTQEEFVDGVVQNSWCEDSRDKEAILIVKVHGNYAIQTRNHRVRVHHCYSSKKNQLTQPNGSRHVQGSVLHRSDFQRLSNSFDVVDQLHKLHSP* |
| Mtr1g071680 | MRDDNLKKSKLSWPKTLVKKWFNIKSKNEDFQADDDVLYGGVDEEWRNNCSKREECTIKKSKIERTKRRHSERSRRCKVDHDAAQVTDMNHYRIFSATWNVAGRSPPSYLNLEDWLHTSPPADIYVLGFQEIVPLNAGNVLGTEDNAPARKWLALIRKTLNSLPGTSGECHTNSPLPDPVVELDSDFEGSMRQKATSFFHRRSFQSLSHSMRMDNDMVVPQACLDRRFSVCDRMIFGHSTGDYEQNYRWGSSDDENGDSPVVAQYSPMLYRGSVSMEDRDRQTENSRYCLVASKQMVGIFLTVWVKSNIRDDVRNMKVSCVGRGLMGYLGNKGSISISMSLHKTSFCFICSHLTSGQKEGDELRRNSDVMEILRKTRFPRVNGIGDESSPQTILDHDRIIWLGDLNYRIALTYRAAKALVEMHNWKVLLENDQLHIEREQGRVFEGWNEGQIYFPPTYKYSNNSDRYTGDDIHSKQKRRTPAWCDRILWHGRGLRQLSYVRGESRFSDHRPVCSVFLAEVESVSRNRIKKCSSCSSSRVEVEELLPHSNGYNYTDLTFY* |
| Mtr1g103330 | MGDENSKNTKLSWSKKMVRKFFNFKSKCEDIQADAVVYGGGEVEYGSRNSFSEREPCTIKKSKTEKFSRSTSQVRRGRMNLDHPRIIDVQNYSIFVATWNVAGRSPPSNLSVDDWLHSSPPADIYVLGFQEIVPLNAGNILGAEDNGPAKKWLALIRKTL  NNLPGTSGSSGCYTPSPIPQPVVELNADFEGSARQKNSSFFHRRSFQTTSSGWGMDNDPSLVQPQVDRRYSVCDRVIFGNRPSDFDPSLRWGYRPSDYSRASDYSRPSDYSRWGSSDDDNGLVDSPSTVLYSPMSTNGGSASNEDGYSMPGHSRYCLVASKQMVGIYLTVWVKGELKDHVRNMKVSCVGRGLMGYLGNKGSISISMSVHETSFCFICSHLTSGQKEGDELRRNSDVMEILKKTRFPRVHGVDNEKSPQTILEHDRIIWLGDLNYRIALSYRSAKALVEMQNWRALLENDQLRIEQKRGRAFVGWNEGKIYFPPTYKYSTNSDRYSGDDMHPKEKRRTPAWCDRILWYGEGLHQLSYVRGESRFSDHRPVYGIFWAEVESPHGKLKKSMSCSQSRIELDELLPYSGGYTELNFF* |
| Mtr1g097690 | MKQQQQKHTANKQQQNLWATMVMRKWLNIKRKESDDYSTDPDDDDDVDDPETDSDNEEWGSRSRIRDRREDEAPAESDEFLPGLRRQKSLTVRSQYINKKELRVCVGTWNVGGKLPPNDFDIDDWLDINHPADIYVLGLQEIVPLNTSNIFVAVDTRPVPKWENLIREALNRVQSKPSKIKSFSDPPSPSKFKPSDDAPDIEEEILLESDSDIGEEIHPLGEEHNVFDGVTDKQIIDEALNISLKDSNASDIAENDLQNQLSYQRKLNRLNHFREEDSSENNETTSSQQISKLSRMVSGTERIGLSWPEPPLHLLPQKVLERPTSFKPDAIDLLADIDLEALMKRKTRSSYVKIVSKQMVGIFITVWVRRSLRKHIQNMKVSTVGVGVMGYIGNKGSISVSMIYQTLFCFICTHLTAGEKEADEIKRNADVREIHQRTHFYSLSDFGLPKSILDHERIIWFGDLNYRISLPYDKTRDLISKKHWSKLVERDQLAKELEKGVFDGWSEGKLNFPPTYKYEINSDKYIGEDPKVARRTPAWCDRILSHGNGMRLLSYKRSELKFSDHRPVTATYMAEVEVFNPKKLQRALNYTDAEIENEEVTTSFGTWNLAT* |
| Mtr1g038810 | MTRKLAEVMWPALVANKILNRRLGSRNFIADYPSYTDPLLSTNNDDQLSLSTKSINDHSDTQKYKVFVSTWNIGGIAPDEGLNIEDLLETCSKSFDIYVFGFQEIVPLNASNVLGSEDSKISTKWNSLIRNALNKRTHHYCCKDIERDDDDNIEQDLKNICQGNIPAQQCKSAPQDFHCIISKQMVGILISVWVRSDLSPFIRHPCVSCVGCGIMGCLGNKGSVSVRFLLHETSFCFVCSHLASGGKEGDEKHRNSNVAEIFSRTSFPKGTILNLPRKILDHDHVILLGDLNYRISLPEETTRLLVEKRDWDSLLENDQLKMELESGQMLRGWHEGTIKFAPTYKYFLNSDEYYGCCYHGMKKAAKKRSPAWCDRIIWLGNGLKQIEYARSESKLSDHRPVKALFTAEVKVSSALKSFPSLFLSERFEQIKNVFEISPTNEFVCKKQSSFRL* |
| Mtr6g092670 | MDDRNIDDDEKESLAGLSSVPPRRKTHSYSQQLRDTSTHKRHHQVRKHSLDDSLISNNIVESSSFYEESDTDDDDFFANSNSVGAEDYIESGGISDDLSHYQPLQEFIGSGGGTGVFKAPIRMAVHPGRPPCLELRPHPLRETQVGKFLRNIACTETQLWAGQECGVRVWEFQKAYEHGCGLGGRVRRGDEDAAPFYESADTSPTFCLTVDNGNKMVWTGHKDGKIRSWKVDQQFSTPFKEGLSWQAHRGPVLAMIISSYGDLWSGSEGGVIKIWPWESIEKSLSMSPEEKHMAALLVERSFIDLRTQVTVNGVCSISSQEVKCFLSDHIRGRVWCATALSFSLWDARTKDLLKVFNIDGQAENRVDMSSVQQDQAVEDEMKVKFVSNSKKEKSQSTSFLQRSRNAIMGAADAVRRVATKGAGAFVDDTKRTEALVQTNDGIIWSGCTNGLLVQWDGSGNRLQDFNRHPCAVQCFCTFGTRIYVGYVSGIIQILDLEGNIIAGWVAHNSPVLKLAVGNGSVYSLATHGGIRGWNIASPGPVDNIIRTELASKELTYTRRHGIRILIGTWNVGQGRATQEALLSWLGSVVSDVGIVVVGLQEVEMGAGFLAMSAAKETVGLEGSAMGQWWLDTIGKALEEGKAFERMGSRQLAGLLISLWVRKNLRKHVGDIDAGAVPCGFGRAIGNKGGVGLRIRVYDRIMCFVNCHLAAHLEAVNRRNADFDHIYKNMVFSRSSTLLNTAAAGVSTSAHMLRSTNAMGSSSEDAKPELSDADMVVFFGDFNYRLFGISYDEARDFVSQRCFDWLREKDQLREEMKNGKVFQGMREAVIKFPPTYKFERHLPGLGGYDSGEKKRIPAWCDRIIYRDTRPAAVSDCNLDCPVVSSILQYDACMDVTESDHKPVRCKFNVRISHADRSIRRKEFGEIMTSNEKIRSMLEESRYVPECNVSPDNLVLENQEASFLLITNRSTNDKAVYKITCDGQSTVKNDGEAPDYTPRGAFGFPRWLEVSPSIGIIKPEQTLEVSVRHEDVHASEELVDGIPQTWWSEDTRDKEVILVVHVQGSSSVQTSSHKIHVRHCFSGKPTRTDSKSNSARRSHVS* |
| Mtr6g009260 | MELKNQKSRYKRLSNWFTTKQKENKPSLSLYEIEDIVEDESDEYGELDSCISTNKLRIFVGTWNVAGRSPVGSLAVDLDEWLNLKNSADIYVLGFQEIVPLKTSTVIGAEDPSVATNWNNLIGKTLNNKFDFPWLTPMLNSSLPNDQENYQNIGGNETNKNKYKMVASKKMVGVFISVWLKEQVLEKYCVSNVRVCSVACGVMGYLGNKGCVGVSMLIEGTSFCFVVAHLASGEKKGDEGRRNHQVEEIFRRTSFPRTTKHHQHYPLTILGHDRIFWFGDLNYRLYLKDHLARHLIRKQDWKALQEFDQLQKELAEGGVFEGWKEGNVEFAPTYKYSSSTSNIYCGGGLPTRSGEKQRTPAWCDRILWYGKGVEQLYYIRSESKFSDHRPVSALFSTHIEIKSSSKELVEMHNIPPTILQSNHGVNKGEEDENFTSLSMLMKNVKGC* |
| Mtr6g072010 | MHHECIFCPKIMQGIKLSESKILRKILSRNSSETKQETTSLNQASENSTRQFYHQTKKIFVGSWNIGGIAPPHNLDIEDWLDTQNDSADIYVLGFQEIVPLNAANVLGPQNKKISMKWNSLIGATLNNKRPMKVVEEDKKAEPQKIYPLKEQTCEEVENVDDFQCIISRQLVGMFITIWARCDLYQSIKHLNVSSVGCGVLGCLANKGSISIRFFLHETSFCFICSHLASGGKEEDKRQRNANAADILSQTNFPVGPLHDLPQKIIDHDRVVWLGDLNYRIDMSHSATQSLIKKREWETLLKHDQLKMELKEGRVFQGWHEGSIEFPPTYKYHPNSDDYIGCNQQHMSKKRRSPAWCDRIIWFGKGMRQIQYNRSESKLSDHRPVQAMFTADIKVAATNYK* |
| Mtr6g042240 | MKERSQNNQQRNWAELCCCSQIQLFWARVVMRKWLNIGSNESDYSADPEDDDEFDEDDDEDDDEHEVWGRKSRFMDNRGFEAPSESNDFVPKLRKQKSSTYRSQYINTKELRVCVGTWNVGGRLPPDDLDIDEWLGVNEPADIYVLGLQEIVPLNAGNIFGSEDTRPVPKWENIIREALNRVRPSVTKTKCFSDPPSPSKFKPSEDDPDIEEEILFESDSDIGEEVHPLDEEQIICDESTTGDNMNTSLLASDVADSASVNTSEPVKIDYRRQFSFPKRFDWPQSPPENMDASISQKAKTLTRMLSGSERIGLSWPEPPLHLLSQRVLDRPTSFKSFKSFKSLKSFKTYNSFKSIMDGMPGMGLLPEIDLEALMKRKRRSPYVRIVSKQMVGIFITVWVRRSLRKHIHNLKVSTVGVGIMGYIGNKGSVSVSMSIYQTLFCFICTHLTSGEKEGDELKRNSDVHEILRRTHFHSPSIIGLPKGILDHERIIWLGDLNYRINLSNVEAKALISKKQWSKLLEKDQLMRELKHGAFGGWSEGALNFPPTYKYEVNSDKYYGDDPKASKRTPAWCDRVLSYGKGMRLLNYRRTELKISDHRPVTATYIVEVEAFSPRKLQRALTFTNAEIENEEAISSLISWK* |
| Mtr4g098850 | MQGSVSVSMSVFQSRMCFVCSHLASGQKDGAEQRRNSDVHEILQRTRFSSVFDTDQPRTIPSHDQIFWFGDLNYRINMSDGEVRKRVALKKWDELMNYDQLSNELCRGHVFEGWKEGLINFPPTYKYEVNSDKYVGEDTQEGEKKRSPAWCDRILWLGKGIKQLKYQSAENQLSDHRPVSSIFLVNVEVIDHRKLQRAINFASAVVHPEIFLEEDRDLAWSYHFGFEVELIKKDSYAVCSDPIVDTTFCITDDVLTSTFCGCGESANEADVSFCITSLNTMKGKRSEVFWPSTVMKKWLNVKQKVYDFSEDEANTETDESEDDDTSCKDYEMKRHRRRKSETLRAQYINTKEVRVTIGTWNVAGKHPCNDLEIEGWLCTEEEPSDIYIIGFQEVVPLNAGNVFGAEDSKPIPKWDALIRRTLNKSSEPGTKKKSNSAPPSPIRRISSFNTQINPLDSALDKKEEIKTIISIEKNLQLSKI  YDIDLQTILDWPELRLDPIHHVDSSPKMRRVQSTSDSASLYGFEMKSLHQSSGNFSLLWSEKQQEIVPQVFDSHLDVSDM  LSDEDNDTFSELANNEDANGIISVKSHPKYVRIVSKQMVGIYVSVWVQRKLRRHVHHLKVSQVGVGLMGYMGNKGSVSVSMSVFQSRMCFVCSHLASGQKDGAEQRRNSDVHEILQRTRFSSVFDTDQPQKIPSHDKIFWFGDLNYRINMSDGEIRKLVDLKKWNELMKFDQLSNELCKGHVFEGWKEGLINFPPTYKYEFNSDKHVGGNTQEGEKRRAPAWCDRILWLGKGIKQLKYQSAENQLSDHRPVSSIFLVDVEVIDHRKLERAIYFASAVVHPDVFLKEDEDEDLSYQ |
