# Supplementary figures and images for: Expansion and Functional Divergence of Inositol Polyphosphate 5-Phosphatases in Angiosperms
Source: Genes (Basel). 2019 May 22;10(5):393. doi: 10.3390/genes10050393 (PMC6562803; doi:10.3390/genes10050393)

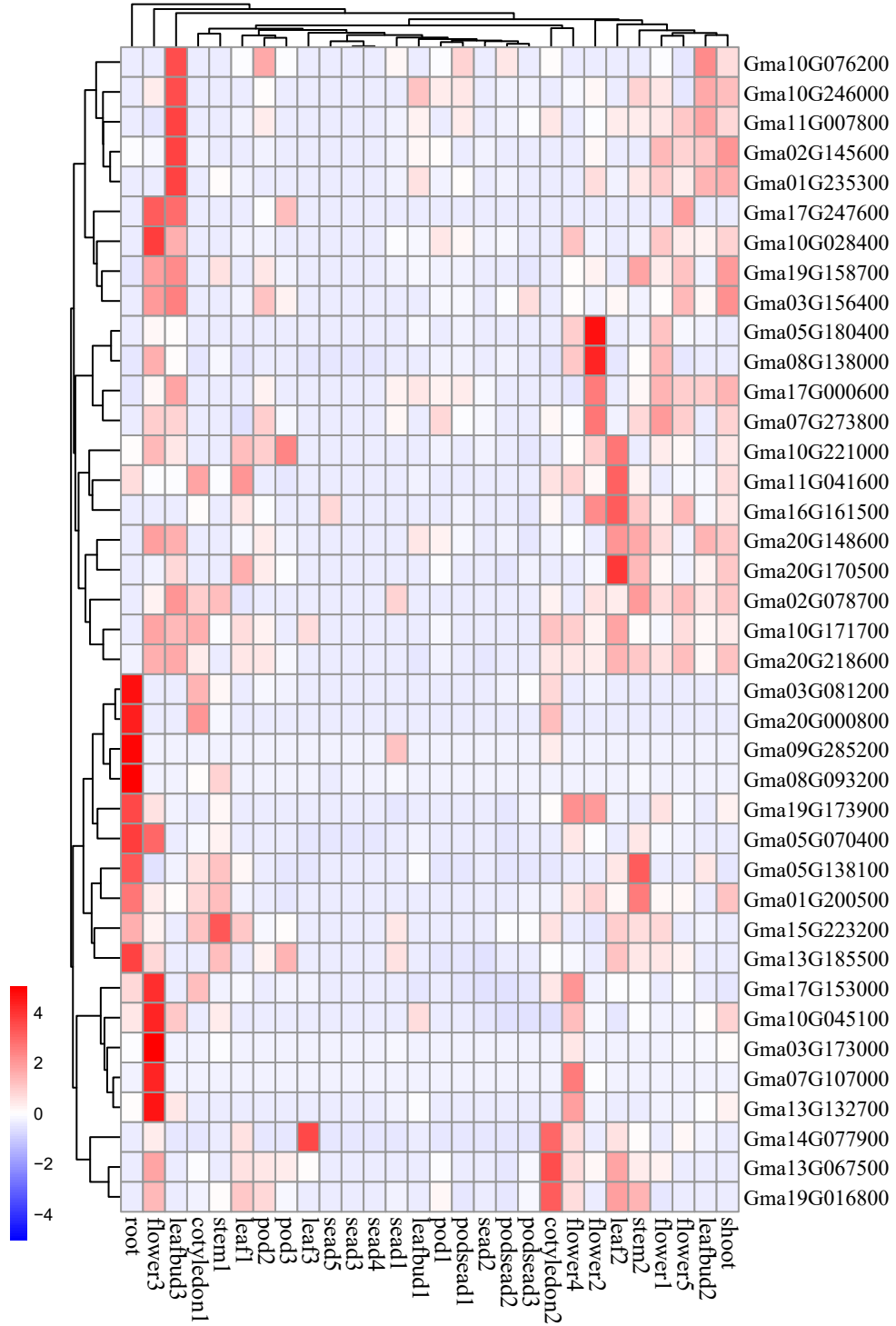

Supplement: Supplementary file 1 [file genes-10-00393-s001.zip › Figure s3.pdf]

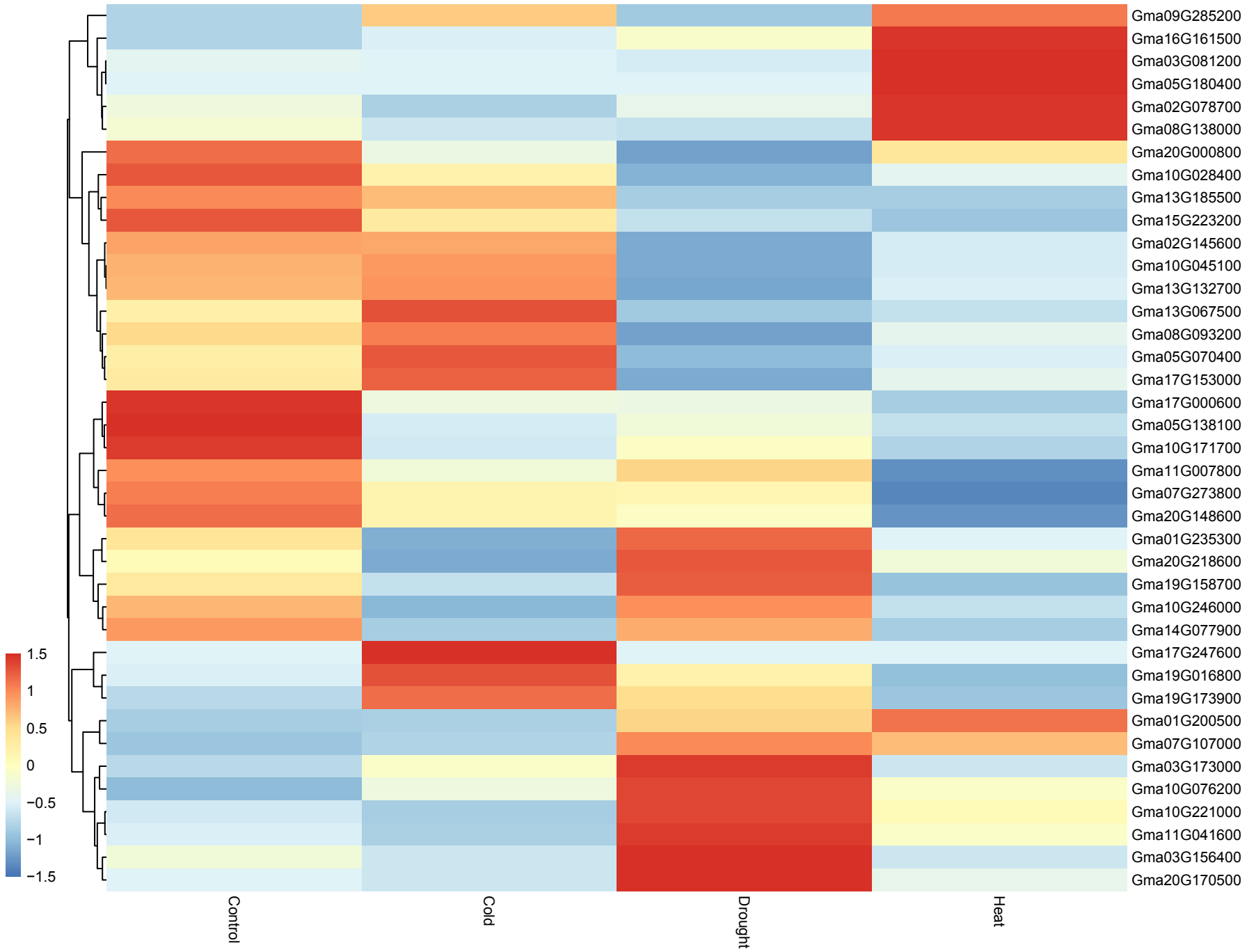

Supplement: Supplementary file 1 [file genes-10-00393-s001.zip › Figure s4.pdf]
