# Supplementary material for: Expansion and Functional Divergence of Inositol Polyphosphate 5-Phosphatases in Angiosperms
Source: Genes (Basel). 2019 May 22;10(5):393. doi: 10.3390/genes10050393 (PMC6562803; doi:10.3390/genes10050393)

figure 3

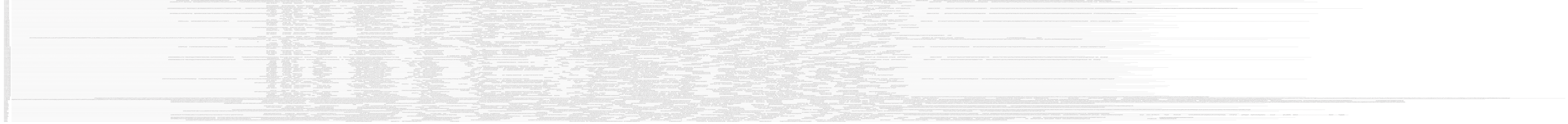

Supplement: Supplementary file 1 [file Figure s2.pdf › Figure s2.pdf]
